# Tuning Electron-Accepting Properties of Phthalocyanines for Charge Transfer Processes

**DOI:** 10.1021/acs.inorgchem.4c00527

**Published:** 2024-04-29

**Authors:** Stefan Bednarik, Jiri Demuth, Jakub Kernal, Miroslav Miletin, Petr Zimcik, Veronika Novakova

**Affiliations:** Faculty of Pharmacy in Hradec Kralove, Charles University, Ak. Heyrovskeho 1203, Hradec Kralove, 500 05 Czech Republic

## Abstract

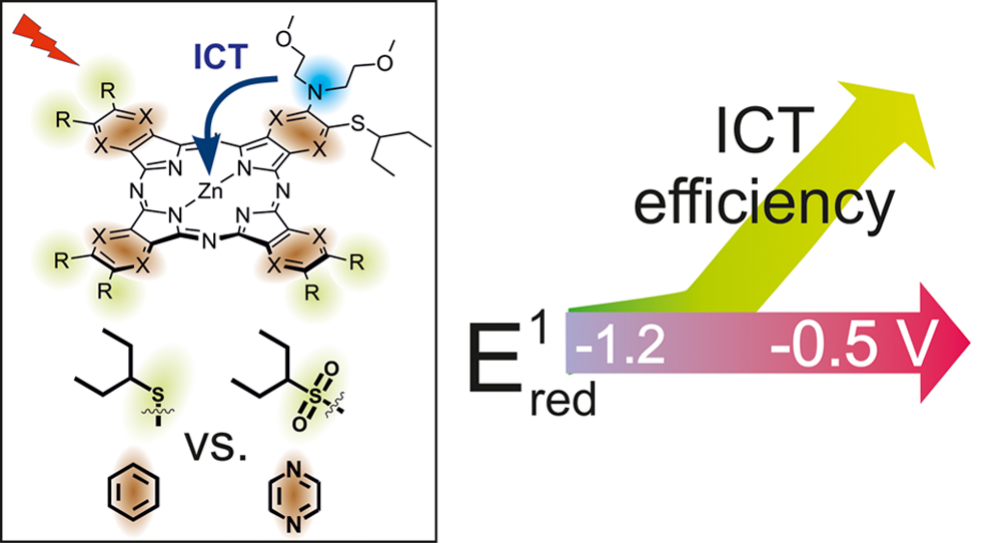

Phthalocyanines play fundamental roles as electron-acceptors in many different fields; thus, the study of structural features affecting electron-accepting properties of these macrocycles is highly desirable. A series of low-symmetry zinc(II) phthalocyanines, in which one, three, or four benzene rings were replaced for pyrazines, was prepared and decorated with electron-neutral (alkylsulfanyl) or strongly electron-withdrawing (alkylsulfonyl) groups to study the role of the macrocyclic core as well as the effect of peripheral substituents. Electrochemical studies revealed that the first reduction potential (*E*_red_^1^) is directly proportional to the number of pyrazine units in the macrocycle. Introduction of alkylsulfonyl groups had a very strong effect and resulted in a strongly electron-deficient macrocycle with *E*_red_^1^ = −0.48 V vs SCE (in THF). The efficiency of intramolecular-charge transfer (ICT) from the peripheral bis(2-methoxyethyl)amine group to the macrocycle was monitored as a decrease in the sum of Φ_Δ_ + Φ_F_ and correlated well with the determined *E*_red_^1^ values. The strongest quenching by ICT was observed for the most electron-deficient macrocycle. Importantly, an obvious threshold at −1.0 V vs SCE was observed over which no ICT occurs. Disclosed results may substantially help to improve the design of electron-donor systems based on phthalocyanines.

## Abstract 2

The efficiency of the charge transfer process substantially improves with increasing electron-deficiency of the macrocycle, which can be easily achieved by introduction of strongly electron-withdrawing groups and/or aza-substitution of the macrocycle. The value of reduction potential is a nice tool to predict the efficiency of the charge transfer process.

## Introduction

Intramolecular charge transfer (ICT) and photoinduced electron transfer (PET) are phenomena observed in certain molecules where an electron is transferred from one part of the molecule to another upon excitation by light. Understanding them and their control allows scientists to design materials with tailored optical, electronic, and chemical properties to suit specific applications such as development of smart photosensitizers,^1^ fluorescent sensing probes and molecular switches,^2−4^ dye-sensitized solar cells,^5^ OLEDs,^6^ or nonlinear optical materials.^7^ The basic prerequisite for ICT is a molecule containing an electron donor and acceptor connected via a conjugated π-system, allowing the electrons to be delocalized over the entire system. In contrast, in the PET process, electrons “jump” from a donor to acceptor separated by a covalent or a noncovalent spacer.^8^

Phthalocyanines (Pcs) have been shown to be excellent systems enabling electron transfer processes;^9−11^ however, most efforts have been devoted to investigation of PET.^10,12,13^ That is why we focused in the past decade more on the description of ICT at Pcs and their aza analogues (AzaPcs). We have shown that the number of donors, the donor type, and the distance between a donor and an acceptor may substantially affect the ICT efficiency.^14,15^ On the other hand, the properties of the acceptor in ICT have received, so far, only limited attention.^16^ In general, it is known that pyrazine analogues have been proven to possess lower reduction potential than corresponding Pcs.^17−19^ As the electron density of the core (the acceptor) may play a substantial role in electron transfer, we decided to focus on this parameter in the present work and evaluate its effect on the efficiency of quenching the excited states by ICT.

**Chart 1:**
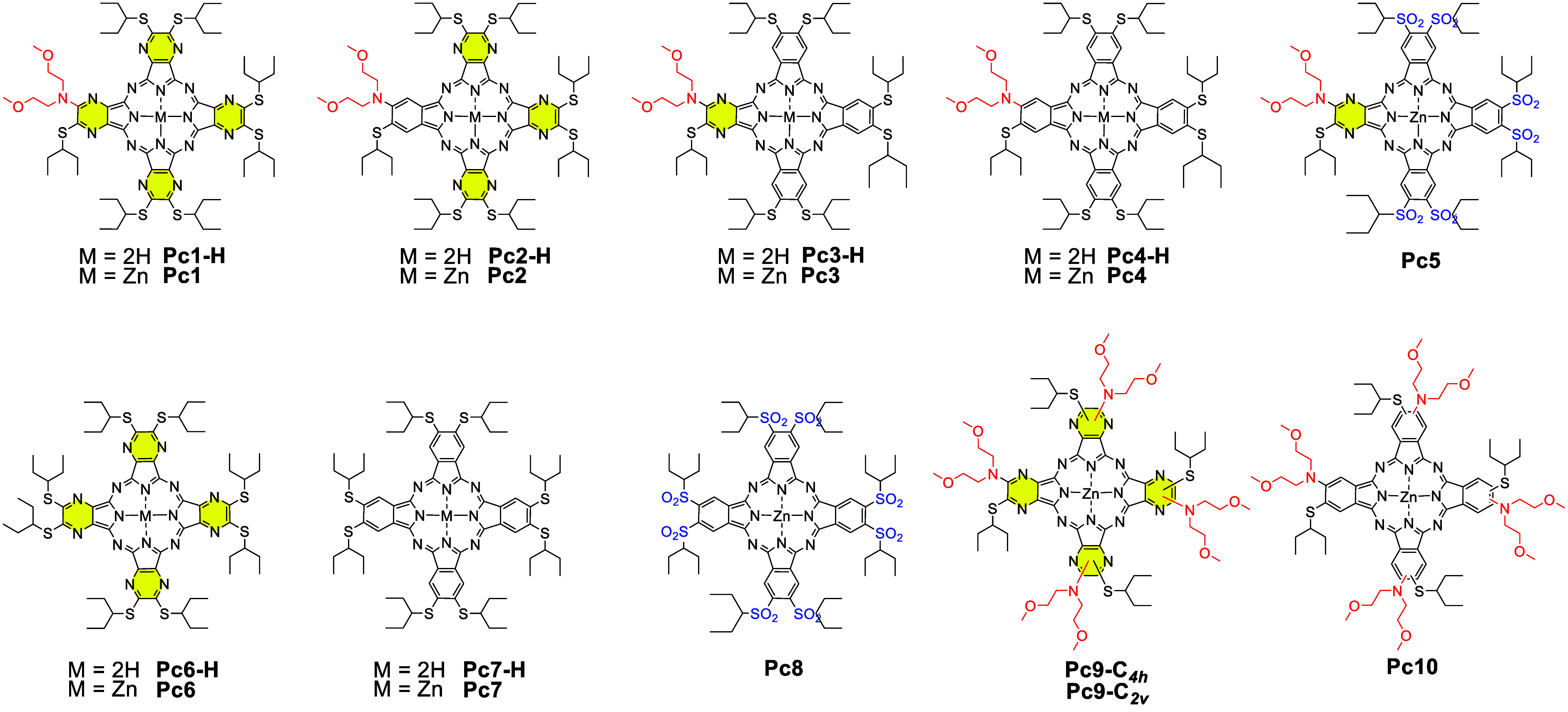
Target Macrocycles (**Pc1**–**Pc5**, **Pc9**, **Pc10**) and Controls (**Pc6**–**Pc8**) Involved in the Studya

All target compounds of the series were designed to have either one (**Pc1**–**Pc5**, Chart 1) or four (**Pc9**, **Pc10**) dialkylamino donor centers for ICT. Also control compounds **Pc6**–**Pc8** without any donor were introduced to the study to compare the results with not-quenched systems. One (**Pc3**, **Pc5**), three (**Pc2**), or four pyrazine (**Pc1**, **Pc6**, **Pc9**) units as well as introduction of electron neutral alkylsufanyl (Hammett substituent constant for the closest available substituent σ_p_(-SCHMe_2_) = 0.07)^20^ or strongly electron withdrawing alkylsulfonyl groups (σ_p_(−SO_2_Et) = 0.77)^20^ enabled the revelation of the impact of these structural features on the electron deficiency of the parent macrocycle and subsequently also the efficiency of ICT. All substituents were designed to be sufficiently bulky to inhibit potential aggregation in organic solvents.

a Pyrazine units are highlighted by yellow, donor substituents by red, and electron-withdrawing substituents by blue color.

## Results and Discussion

### Synthesis

**Scheme 1:**
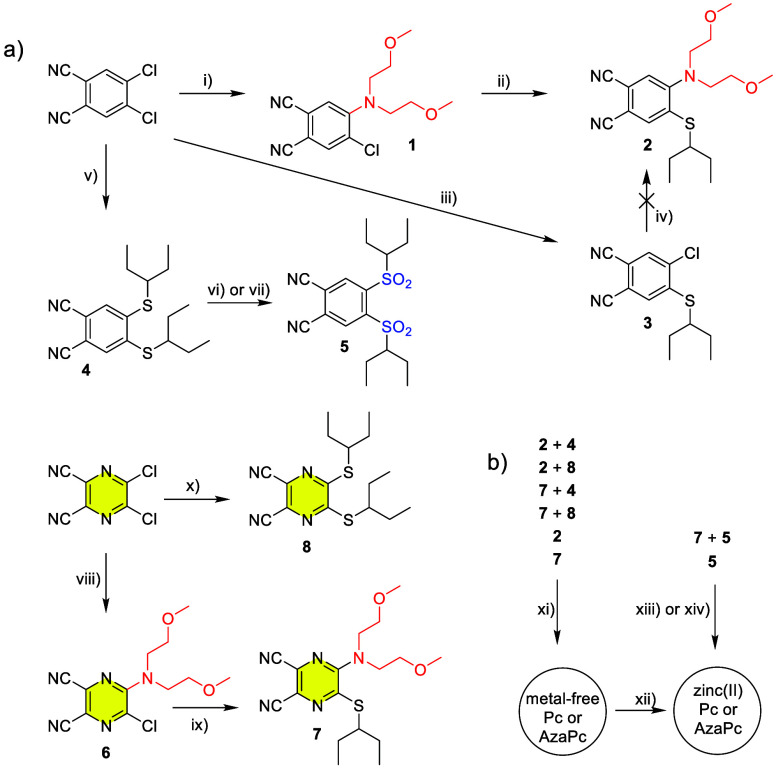
Synthesis of Precursors (a) and Target Macrocycles (b): (i) bis(2-methoxyethyl)amine, K_2_CO_3_, anh. DMSO, rt, 240 h, 9%; (ii) pentane-3-thiol, K_2_CO_3_, DMSO, 60 °C, 48 h, 87%; (iii) pentane-3-thiol (1.35 equiv), K_2_CO_3_, DMSO, 60 °C, overnight, 85%; (iv) bis(2-methoxyethyl)amine, K_2_CO_3_, DMSO, 110 °C, 24 h, 0%; (v) pentane-3-thiol (2.5 equiv), K_2_CO_3_, anh. DMSO, rt, 72 h, 77%; (vi) *m*-CPBA, DCM, rt, 18 h, 14-73%; (vii) H_2_O_2_, AcOH, reflux, 2 h, 52%; (viii) bis(2-methoxyethyl)amine, THF, −12°C, 2 h, 81%; (ix) pentane-3-thiol, NaOH, THF, rt, 2 h, 64%; (x) pentane-3-thiol, NaOH, THF, rt, 2 h, 72%; (xi) Mg(BuO)_2_, BuOH, reflux, 18 h followed by TsOH, THF, rt, 2 h, 13–24%; (xii) Zn(OAc)_2_, pyridine, reflux, 30 min, 18–84%; (xiii) Zn(OAc)_2_, anh. pyridine, reflux, 18 h, 5%; (xiv) Zn(OAc)_2_, *o*-dichlorobenzene/anh. DMF, 135 °C, 18 h, 84%

Pcs and AzaPcs are typically synthesized by cyclotetramerization of their precursors, i.e., 4,5-disubstituted phthalonitriles and 5,6-disubstituted pyrazine-2,3-dicarbonitriles, respectively.^21,22^ Aside from precursor **5**, nucleophilic substitution was employed for the synthesis of all required precursors and their intermediates (Scheme 1a). This reaction proceeded much better in pyrazine derivatives due to negative inductive effect of pyrazine nitrogens, which makes positions 5 and 6 more electron deficient. Thus, pyrazine analogue **6** was isolated in 81% yield while compound **1** was obtained in a yield of 9% only. The unwillingness of the benzene ring toward nucleophilic substitution is further documented by an unsuccessful attempt to prepare desired precursor **2** in reverse order, i.e., from intermediate **3**. Attempts to use Buchwald–Hartwig amination for the synthesis of **1** failed as well. Different reactivity was not, however, observed with thiolates as stronger nucleophiles since **2** (87%) and **4** (77%) and their pyrazine analogues **7** (64%) and **8** (72%) were obtained in comparable yields. Two different procedures were employed for the oxidation of sulfur in compound **4** to get precursor **5** bearing sulfonyl groups, i.e., *m*-chloroperoxybenzoic acid (*m*-CPBA) in DCM or H_2_O_2_ in AcOH. The latter method seems to be preferable because the oxidation by *m*-CPBA gave inconsistent yields when repeating the procedure (the yields in different batches were 14%, 15%, and 73%) while the oxidation with H_2_O_2_ gave constant yields over 50%. Unfortunately, all attempts to oxidize pyrazine analogue **7** failed.

Target macrocycles (**Pc1**–**Pc4**) were synthesized by cyclotetramerization starting from two different precursors: A (i.e., precursor **2** or **7** containing the dialkylamino donor responsible for ICT) and B (i.e., precursor **4** or **8**) in a ratio of 1:3 under Linstead conditions (Scheme 1b). The statistical condensation of precursors A and B led to the formation of a mixture of six congeners coordinating the magnesium(II) cation in the center (AAAA, AAAB, AABB, ABAB, BBBA, and BBBB), of which the ABBB and some BBBB types were of interest. Based on our experience, metal-free derivatives are less prone to tail on silica;^23^ therefore, the obtained mixture of magnesium(II) congeners was directly converted to the corresponding metal-free derivatives by treatment with an excess of TsOH in THF. Metal-free ABBB and symmetric BBBB congeners (for **Pc6** and **Pc7**) were then isolated by column chromatography on silica in reasonable yields of 13–24%. Finally, zinc(II) was introduced to the center of macrocycles by heating metal-free derivatives in pyridine with an excess of anhydrous zinc(II) acetate.

Macrocycles **Pc5** and **Pc8** containing sulfonyl groups at the periphery could not be prepared by the Linstead method due to undesirable replacement of the peripheral pentan-3-ylsulfonyl group for butoxy groups by magnesium butoxide employed as an initiator of the reaction. Therefore, they have to be synthesized via a template method. A mixture of *o*-DCB/anhydrous DMF (3:1) was used to prepare symmetric **Pc8** in a good yield of 84%. In the case of low-symmetry **Pc5**, pyridine and a mixture of *o*-DCB/anhydrous DMF (3:1) were used as solvents, both reactions giving **Pc5** in a yield of 5%. Of note, we also performed cyclotetramerization of precursor **2** with **5**; the reaction proceeded well. However, we were not able to separate the desired ABBB due to similar retention factors in all mobile phases tested.

**Figure 1:**
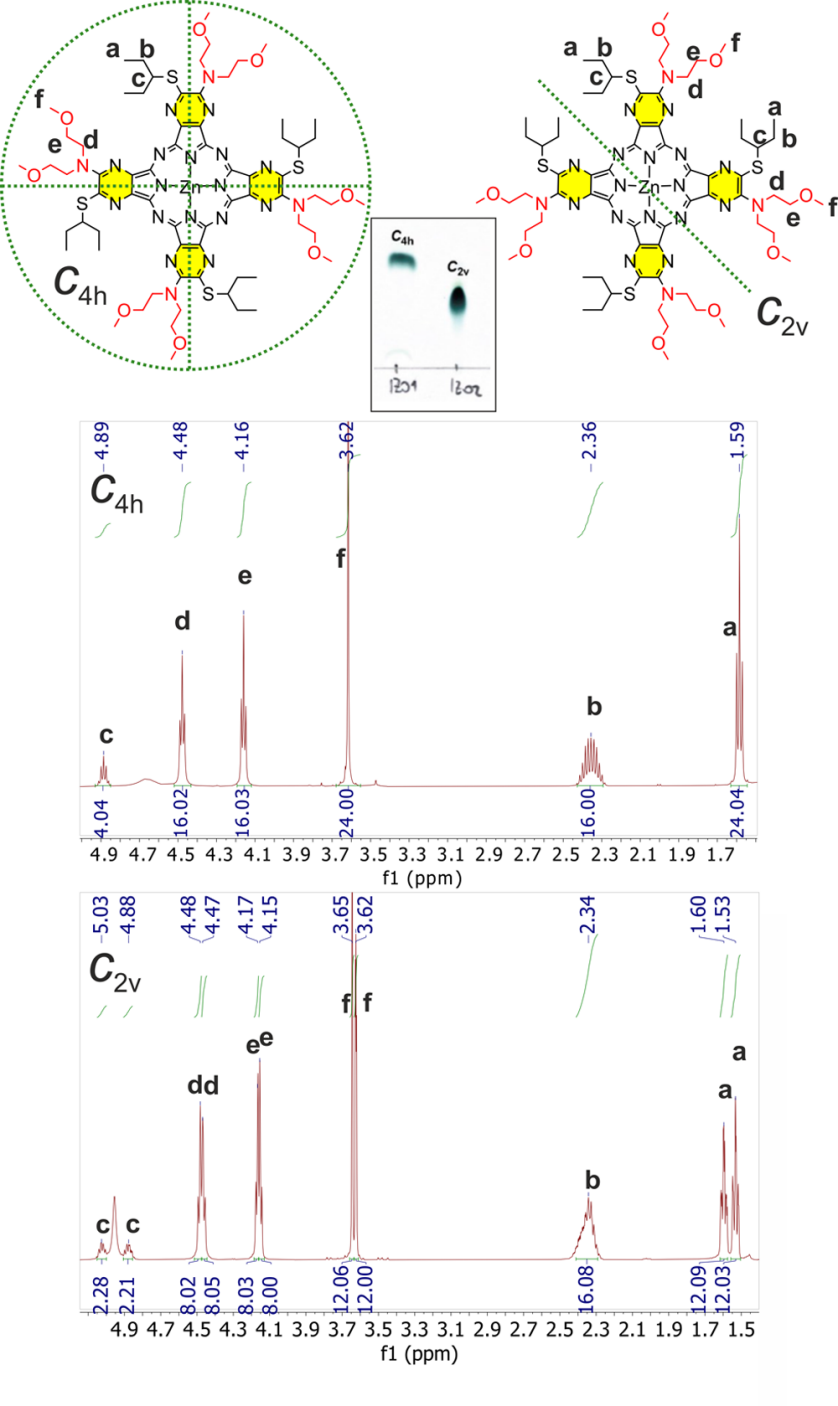
Assignment of isolated fractions of **Pc9** using ^1^H NMR spectra (500 MHz, CDCl_3_/pyridine-*d*_5_ 3:1) to positional isomers **Pc9-*****C***_**4*h***_ and **Pc9-*****C***_**2*v***_. The mobile phase used for the TLC was a 10:1 toluene/pyridine. Green dotted lines indicate the symmetry of the skeleton of the molecule.

Macrocycles **Pc9** and **Pc10** were prepared by the cyclotetramerization of **7** and **2**, respectively, under Linstead conditions. Due to the nonsymmetry character of these precursors, a mixture of positional isomers of **Pc9** and **Pc10** was formed (*C*_2*v*_, *C*_4*h*_, *C*_*s*_, *D*_2*h*_). In the case of pyrazine derivative **Pc9**, we were even able to separate two predominant isomers, which is quite rare in the literature and typically requires special techniques.^24−29^ The structure of particular isomers was assigned using NMR analysis (Figure 1). The fraction with an *R*_f_ factor of 0.40 (mobile phase toluene/pyridine 10:1) is most likely the *C*_4*h*_ isomer because just one set of signals in the ^1^H NMR spectrum was observed, which can be unambiguously assigned to bis(2-methoxyethyl)amino (i.e., signals at δ = 4.48, 4.16, and 3.62 ppm) and pentan-3-ylsulfanyl groups (i.e., signals at δ = 4.89, 2.36, and 1.59 ppm). Such a set of signals is in agreement with the high symmetry of the *C*_4*h*_ isomer. To note, a similar character of signals could be present also for the *D*_2*h*_ isomer.^27^ However, the *C*_4*h*_ isomer is usually obtained in higher yields than the *D*_2*h*_ and was shown to elute as the first fraction if the column chromatography is used for the congener’s separation.^24,28^ The presence of two sets of signals in an equivalent ratio of 1:1 clearly confirmed that the second fraction with *R*_f_ = 0.20 corresponds to the *C*_2*v*_ isomer, which has just one axis of symmetry (Figure 1). The other isomers were formed most likely as well but in very low yields and were barely detected on TLC as tiny inseparable spots only.

### Electrochemistry

**Figure 2:**
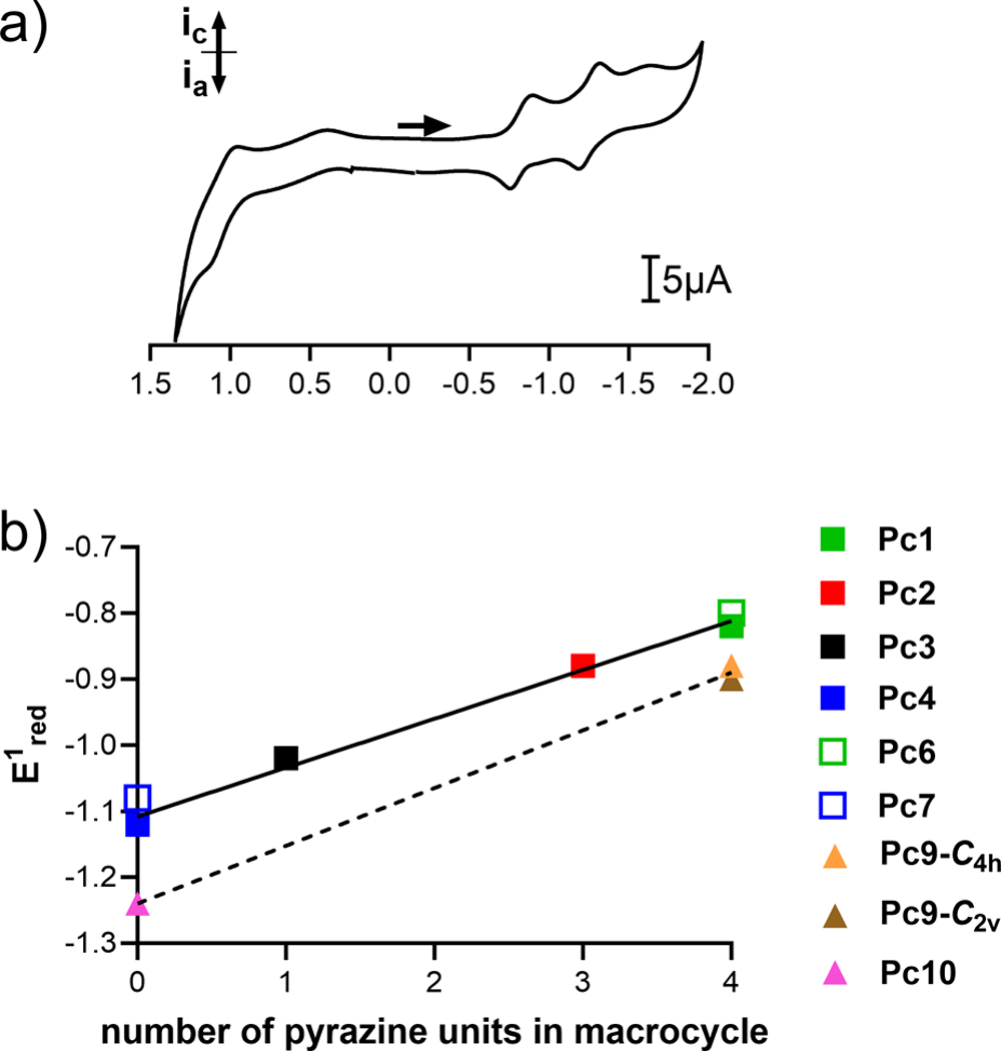
(a) Cyclic voltammogram of **Pc1** as a model compound of the series (in THF, rt, potential step 5 mV, scan rate 100 mV/s, tetrabutylammonium hexafluorophosphate as supporting electrolyte, potential vs SCE was determined according to oxidation of ferrocene used as internal standard (*E*_(Fc/Fc+)_ = 0.56 V vs SCE^31^)). (b) Dependance of *E*_red_^1^ values on the number of pyrazine units in the macrocycle. The lines represent linear regression of the data for compounds bearing one donor center (solid line) and four donor centers (dashed line). Empty square = no donor for ICT; full squares = one donor for ICT; full triangles = four donor centers for ICT.

Cyclic and square-wave voltammetry of **Pc1**–**Pc10** were performed in THF at room temperature. The relevant *E*_red_ and *E*_ox_ potentials were determined from square-wave voltammetry using ferrocene (Fc/Fc^+^) as an internal standard and tetrabutylammonium hexafluorophosphate as a supporting electrolyte. The data are summarized in Table 1, and voltammograms are shown in Figures 2a, S27, and S28. It should be noted that both isomers of **Pc9** had almost identical electrochemical behavior in both oxidation and reduction parts, and that is why it can be considered as not being affected by the different arrangement of substituents around the core in the below derived relationships.

**Table 1:** Spectral and Photophysical Properties of the Target Macrocycles^a^

		λ_A_ (log ε), nm	λ_F_, nm	Φ_F_	Φ_Δ_	Φ_Δ_ + Φ_F_	*E*_red_^2^, V	*E*_red_^1^, V	*E*_ox_^1^, V	*E*_ox_^2^, V
cpd.	structural features^b^	THF	THF	THF/DMF	THF/DMF	THF/DMF	THF	THF	THF	THF
**Pc1**	4 × Pz, 1 × N	650 (5.36)	664	0.043/0.023	0.23/0.20	0.27/0.22	–1.26	–0.82	1.12	1.42
**Pc2**	3 × Pz, 1 × N	659, 670 (5.23, 5.22)	679	0.037/0.011	0.26/0.11	0.30/0.12	–1.26	–0.88	-c	
**Pc3**	1 × Pz, 1 × N	692 (5.44)	702	0.16/0.13	0.55/0.67	0.71/0.80		–1.02	0.82	
**Pc4**	0 × Pz, 1 × N	706 (5.50)	714	0.17/0.12	0.52/0.66	0.69/0.78	–1.78	–1.12	0.68	1.10
**Pc5**	1 × Pz, 1 × N, SO_2_	672, 690 (5.04, 5.05)	695	0.0025/0.011	0.037/0.090	0.040/0.10	–0.86	–0.48	-c	
**Pc6**	4 × Pz, 0 × N	649 (5.47)	655	0.28/0.23	0.52/0.60	0.80/0.83	–1.26	–0.80	-c	
**Pc7**	0 × Pz, 0 × N	706 (5.45)	713	0.26/0.23	0.54/0.61	0.80/0.84	–1.70	–1.08	0.80	
**Pc8**	4 × Pz, 0 × N, SO_2_	684 (5.36)	692	0.23/0.16	0.43/0.63	0.66/0.79	–0.84	–0.36	-c	
**Pc9-C**_***4h***_	4 × Pz, 4 × N	654 (5.28)	664	0.068/0.030	0.36/0.21	0.43/0.24	–1.08	–0.88	0.90	1.06
**Pc9-*C***_**2*****v***_	4 × Pz, 4 × N	654 (5.30)	664	0.063/0.030	0.37/0.21	0.43/0.24	–1.12	–0.90	0.86	1.10
**Pc10**	0 × Pz, 4 × N	703 (5.29)	711	0.16/0.11	0.53/0.56	0.69/0.67	–1.60	–1.24	0.64	1.02

a Absorption maximum at the Q band (λ_A_), extinction coefficient (ε), fluorescence emission maximum (λ_F_), fluorescence quantum yield (Φ_F_), singlet oxygen quantum yield (Φ_Δ_), half-wave reduction potential (*E*_red_), and half-wave oxidation potential (*E*_ox_). Unsubstituted zinc(II) phthalocyanine (ZnPc) was used as the reference (Φ_ΔZnPc(THF)_ = 0.53;^32^ Φ_ΔZnPc(DMF)_ = 0.56;^33^ Φ_FZnPc(THF)_ = 0.32^34^); Φ_F_ and Φ_Δ_ are expressed as the mean of three independent measurements; estimated error ± 15%. Potentials *E*_red_ and *E*_ox_ were measured in THF and are expressed as *E*_1/2_ (in V vs SCE) with Fc/Fc^+^ as an internal standard.

b Pz = number of pyrazine units, *N* = number of dialkylamino donors, SO_2_ = contains alkylsulfonyl substituent(s).

c The values could not be precisely determined due to broad waves in SWV and CV.

All compounds of the series underwent one or two irreversible oxidations, which can be attributed to oxidation of the macrocyclic core and a peripheral amine. The obtained *E*_ox_ values are in agreement with the values published in the literature for similar macrocycles.^17,30^ The ability of a macrocyclic core to behave as an electron acceptor may be assessed from a comparison of *E*_red_ values within the series; the less negative the value is, the better the electron-deficient character of the compound can be expected. Interestingly, the *E*_red_ values were directly proportional to the number of pyrazine units in the macrocycle (Figure 2b). As an example, the *E*_red_^1^ values of **Pc1** (four pyrazine units), **Pc2** (three pyrazine units), **Pc3** (one pyrazine unit), and **Pc4** (no pyrazine units), which have identical peripheral substitutions but differ in the number of pyrazine units only, were −0.82, −0.88, −1.02, and −1.12 V vs SCE. This trend is further confirmed by reduction processes of controls **Pc6** and **Pc7** without any donor, since their *E*_red_ values corresponds to the parent type of macrocycle (*E*_red_^1^ = −0.80 and −1.08 V vs SCE for **Pc6** (four pyrazine units) and **Pc7** (no pyrazine unit), respectively). Reduction of **Pc9** and **Pc10** is also in agreement with this trend (obvious from the parallelism of both regression curves shown in Figure 2b), but *E*_red_^1^ values are shifted to more negative values (*E*_red_^1^ = −0.88, −0.90, and −1.24 V vs SCE for **Pc9-*****C***_**4*h***_ (four pyrazine units), **Pc9**-***C***_**2*v***_ (four pyrazine units), and **Pc10** (no pyrazine unit), respectively). This is clearly caused by the presence of four strongly electron-donating dialkylamino substituents (σ_p_(-NEt_2_) = −0.72)^20^ instead of one in the series **Pc1**–**Pc4** that affected the electron density of the whole core. Its donating effect can be clearly demonstrated in the series of compounds without any pyrazine, i.e., **Pc7**, **Pc4**, and **Pc10** with zero, one, and four dialkylamino donors, respectively, since their *E*_red_^1^ value drops in line −1.08, −1.12, and −1.24 V vs SCE, respectively. A similar trend can be observed also in the series with four pyrazines (i.e., **Pc6**, **Pc1**, and **Pc9**-***C***_**2*v***_/***C***_**4*h***_). On the other hand, the electron deficiency of Pcs can be significantly increased by the introduction of strongly electron-withdrawing alkylsulfonyl groups, which is nicely demonstrated by comparison of **Pc3** and its oxidized analogue **Pc5** (*E*_red_^1^ = −1.02 and −0.48 V vs SCE, respectively) or similarly **Pc7** and its oxidized analogue **Pc8** (*E*_red_^1^ = −1.08 and −0.36 V vs SCE, respectively). To note, the effect of introduction of such electron-withdrawing groups seems to be much stronger than isosteric replacement of benzenes for pyrazines, which is evident from the comparison of **Pc3** (*E*_red_^1^ = −1.02 V vs SCE) with either its pyrazine analogue **Pc1** (*E*_red_^1^ = −0.82 V vs SCE) or its sulfonyl-analogue **Pc5** (*E*_red_^1^ = −0.48 V vs SCE). These results clearly proved that the degree of electron deficiency of the Pc macrocycle may be easily tuned by introduction of a specific number of pyrazine units into the Pc core or by introduction of strongly electron-withdrawing groups.

### Spectral Properties

**Figure 3:**
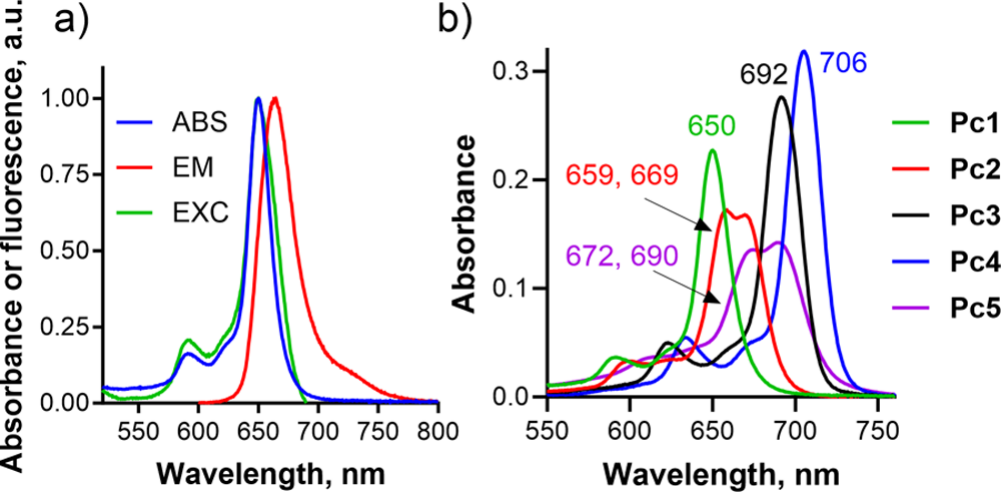
(a) Normalized absorption (blue), fluorescence emission (red), and fluorescence excitation (green) spectra of **Pc1** as a model compound of the series in THF (*c* = 1 μM). (b) Comparison of absorption spectra of **Pc1**–**Pc5** in THF (*c* = 1 μM).

First, the UV–vis spectra of the studied compounds were measured in THF. Data are shown in Figures 3 and S26 and summarized in Table 1. All compounds of the series exhibited a characteristic absorption band, i.e., a low-energy Q band in the range of 650–706 nm and a high energy B band around 370 nm. In most of the zinc(II) derivatives (except **Pc2** and **Pc5**), the Q band remained unsplit, which is indicative of their effective 4-fold symmetry. Thus, peripheral substitution by alkylsulfanyl or dialkylamino groups has limited effect on macrocycles’ symmetry. On the other hand, isosteric replacement of more benzenes for pyrazines (**Pc2**) or the presence of strongly electron-withdrawing alkylsulfonyl groups (**Pc5**) results in significant disruption of the macrocycle’s electron-density distribution, leading to a split Q band. Further, isosteric replacement of benzenes for pyrazines caused a hypsochromic shift in Q band maxima by 14 nm per one pyrazine unit (Figure 3b), which is consistent with the literature.^15,17^ Thus, absorption maxima of the Q band (λ_A_) were proportional to the number of pyrazine units as follows: **Pc1** < **Pc2** < **Pc3** < **Pc4**. Alkylsulfonyl groups led to a slight hypsochromic and hypochromic shift of the Q-band maxima (compare λ_A_ = 713 and 692 nm for **Pc7** and **Pc8**, respectively) as the missing lone pairs of the oxidized sulfur cannot contribute to the conjugated system. Absorption spectra of both isomers of **Pc9** were identical. The fluorescence emission maxima in THF were mirror images of particular absorption spectra. The monomeric character of all compounds of the series in THF was obvious from the sharp and intensive Q band, which perfectly matched the shape of the corresponding fluorescence excitation spectrum. Monomeric character and the same dependences were observed also in DMF that was later used as a solvent in photophysical measurements.

### Photophysical Properties

Fluorescence emission and intersystem crossing followed by energy transfer to molecular oxygen forming highly reactive singlet oxygen are the two main relaxation pathways of the excited states of Pcs and related macrocycles. The probability by which the pathway molecule relaxes can be studied by the determination of particular quantum yields, i.e., fluorescence quantum yield (Φ_F_) and quantum yield of singlet oxygen production (Φ_Δ_). The sum of these quantum yields (Φ_F_ + Φ_Δ_) should be close to 1.0 if molecules are in the monomeric state and other relaxation pathways are not involved. However, the presence of a donor of electrons (e.g., dialkylamine) may lead to ultrafast relaxation via ICT. This has been demonstrated in our previous study by several methods including transient absorption spectroscopy that unequivocally confirmed the presence of the ICT state. In a compound bearing one donor (structurally almost identical to **Pc1**), the ICT state was populated from the S_1_ state with a time constant of 10 ps and recovered to the ground state with a time constant of 115 ps.^23^

To characterize consequences of ICT on the photophysical properties of studied derivatives, we first focused on the fluorescence lifetimes, fluorescence quantum yields, and Stokes shifts dependent on the orientation polarizability of the solvent (Δ*f*) of **Pc6**, **Pc1**, and **Pc9-*C***_**2*****v***_ as examples of Pcs bearing the same core but different numbers of donor units (zero, one, four, respectively), Table 2. Pyridine as a coordinating ligand (0.1% v/v) had to be added to some noncoordinating solvents to eliminate the formation of J dimers^35^ and was believed not to influence properties of the solvent. The presence of J dimers would add another level of complexity that would not allow for clear conclusions.

**Table 2:** Photophysical Properties of **Pc6**, **Pc1**, and **Pc9-*C***_**2*****v***_ (No, One, or Four Donors, Respectively) in Different Solvents

		τ_F_, ns			Φ_F_			ν_A_–ν_F_, cm^–1^		
solvent	Δ*f*^a^	**Pc6**	**Pc1**	**Pc9-*C***_**2*****v***_	**Pc6**	**Pc1**	**Pc9-*C***_**2*****v***_	**Pc6**	**Pc1**	**Pc9-*C***_**2*****v***_
CCl_4_^b^	0.011	2.41	1.86	1.71	0.25	0.162	0.139	89	175	204
toluene^b^	0.013	2.42	1.79	1.61	0.26	0.148	0.127	116	174	193
dioxane	0.025	2.51	2.15 (24%). 0.91 (76%)	1.13	0.27	0.067	0.090	130	241	235
anisole^b^	0.111	2.37	1.71 (28%), 0.80 (72%)	0.95	0.26	0.085	0.065	118	206	208
chlorobenzene^b^	0.143	2.37	1.87 (23%), 0.89 (77%)	1.01	0.31	0.079	0.066	103	204	203
ethyl acetate	0.200	2.68^c^	2.13 (27%), 0.67 (73%)	1.02	0.29	0.053	0.072	102	218	203
THF	0.210	2.28	2.13 (49%), 0.43 (51%)	0.92	0.31	0.034	0.062	122	304	227
pyridine	0.212	2.53^c^	1.89 (76%), 0.17 (24%)	1.03 (14%), 0.32 (86%)	0.24	0.019	0.016	125	380	238
DMSO	0.263	2.30	1.66 (80%), 0.13 (20%)	0.59 (25%), 0.20 (75%)	0.28	0.013	0.007	153	398	224
DMF	0.274	2.29	1.76 (61%), 0.23 (39%)	0.66 (62%), 0.31 (38%)	0.23	0.018	0.019	129	347	206
acetonitrile	0.305	2.50	2.02 (73%), 0.20 (27%)	0.90 (14%), 0.27 (86%)	0.22	0.020	0.011	116	385	200

a Δ*f* = [(ε – 1)/(2ε + 1)] – [(*n*^2^ – 1)/(2*n*^2^ + 1)] where ε is dielectric constant and *n* is refractive index of the solvent.

b Pyridine (0.1% v/v) was added to eliminate formation of J-dimers.

c These decays were biexponential with a very small contribution (1–2%) of the second very fast component (τ_F_, <0.05 ns).

**Figure 4:**
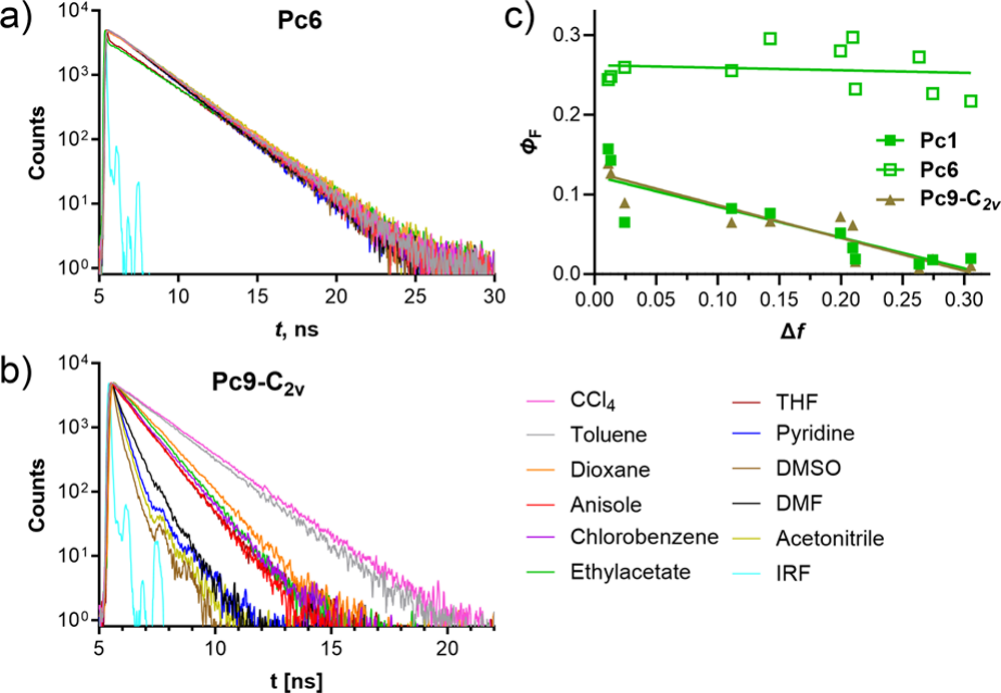
(a, b) Fluorescence intensity decay curves of compounds **Pc6** and **Pc9-*C***_**2*****v***_ in different solvents. IRF = instrument response function. (c) Fluorescence quantum yield of **Pc1**, **Pc6**, and **Pc9-*C***_**2*****v***_ dependent on the orientation polarizability of the solvents (Δ*f*).

Fluorescence lifetimes of **Pc6** without any donor were characterized by monoexponential decays with τ_F_ ∼ 2.4 ns, irrespective of the used solvent. On the other hand, significantly shorter fluorescence lifetimes were observed for both donor(s)-bearing macrocycles **Pc1** and **Pc9-*C***_**2*****v***_ where the decays become faster with increasing polarizability of the solvent (Table 2, Figures 4a,b and S29) as a consequence of competitive ICT whose efficiency also increases with increasing Δ*f*. In more polar solvents, the decays of these two derivatives became even biexponential with a second faster component. In accordance with these results, fluorescence quantum yields of **Pc6** did not change with the change of the solvent while a significant decrease in Φ_F_ was observed for both **Pc1** and **Pc9-*C***_**2*****v***_ (Table 2, Figure 4c) in a more polar solvent. Both of these parameters (Φ_F_ and τ_F_) therefore perfectly correlate with increasing feasibility of ICT in more polar solvents.

**Figure 5:**
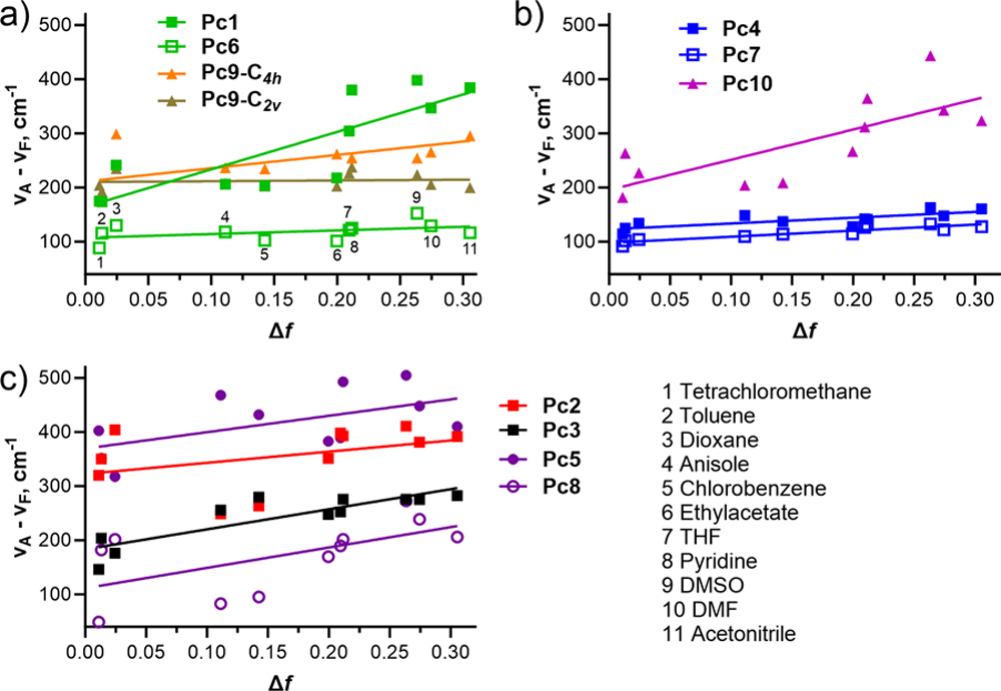
Lippert–Mataga plots of the studied derivatives in the different solvents (solvents are assigned to numbers in box “a” for **Pc6**). (a) Compounds bearing only pyrazine rings in the macrocycle. (b) Compounds bearing only benzene rings in the macrocycle. (c) Compounds with mixed macrocycle and/or bearing strongly electron withdrawing alkylsulfonyls.

Determination of Stokes shifts in different solvents and subsequent Lippert–Mataga plots may give information about different dipole moments in the molecule in both ground and excited states.^36^ As the ICT state leads to redistribution of electron density in the excited state, the Lippert–Mataga plot may potentially also provide useful information. As seen from Figure 5a, the slope of the plot for **Pc6** is almost zero, indicating limited differences between dipole moments in the ground and excited states while a very steep slope was determined for **Pc1** with one donor. However, **Pc9-*C***_**2*****v***_ bearing four donor centers was also almost parallel with axis *x* (Figure 5a) despite exerting strong ICT as determined above. For this reason, we extended these experiments to the whole series of compounds. As seen from the slopes, the induced dipole moment in the excited state in the Pc macrocycle did not correspond to the ICT efficiency (see also the data from quantum yields below). For example, **Pc8** with no donor and no ICT but with strongly electron withdrawing alkylsulfonyls had a rather steep slope of the plot (Figure 5c). As another example, the isomers of **Pc9**, despite having the same photophysical properties and number of donors (Table 2 and discussion below), strongly differed in the Lippert–Mataga plots. For this reason, these experiments, although reflecting changes in dipole moments in the ground and excited states, do not correlate with ICT and rather reflect local electron distribution due to different geometry and/or electronic effect of substituents.

Besides the polarity of the medium and type of the donor of electrons,^37^ feasibility of ICT depends also on the electron-accepting ability of the macrocycle—the factor that we focused on in this work in more detail. As a competitive pathway to fluorescence and singlet oxygen, the sum of Φ_F_ + Φ_Δ_ is an excellent tool to monitor the efficiency of ICT; the lower the value, the higher the ICT efficiency. We focused on two rather polar solvents (THF, DMF) in which all of the studied derivatives are in monomeric form to have two independent sets of measurements.

Φ_Δ_ values were measured by the chemical reference method using 1,3-diphenylisobenzofuran as a singlet-oxygen quencher and unsubstituted zinc(II) phthalocyanine as a reference (Φ_Δ(THF)_ = 0.53,^32^ Φ_Δ(DMF)_ = 0.56^33^). Φ_F_ values were determined by the reference method after excitation in the Q-band region with unsubstituted zinc(II) phthalocyanine as a reference (Φ_F(THF)_ = 0.32^34^). Data are listed in Table 1.

Generally, zinc(II) Pcs and their aza analogues possess Φ_Δ_ ∼ 0.50–0.70 and Φ_F_ ∼ 0.20–0.30 in various organic solvents, which is also true for controls **Pc6**–**Pc8** without any donor center. This indicates that studied compounds are in monomeric form in DMF and THF and that other relaxation pathways do not take a part. Introduction of the bis(2-methoxyethyl)amine group as a donor for ICT in **Pc1**–**Pc5** and **Pc9** and **Pc10** led to a decrease in Φ_Δ_ and Φ_F_; however, big differences in the sum of Φ_F_ + Φ_Δ_ were observed within the series. Concerning **Pc1**–**Pc4** having one such donor, the presence of more pyrazine units in the macrocycle was necessary to observe any decrease in Φ_Δ_ + Φ_F_. **Pc3** (with one pyrazine) and **Pc4** (with no pyrazine) had quantum yields nearly reaching controls **Pc6** and **Pc7** without the possibility of ICT (Φ_Δ_ + Φ_F_ ∼ 0.7 and ∼0.8 for donor-containing and controls, respectively), but the presence of four pyrazines in **Pc1** resulted in significantly reduced Φ_Δ_ + Φ_F_ = ∼0.2. Importantly, a distinctive drop in Φ_Δ_ + Φ_F_ with the lowest value within the series was reached in **Pc5** (Φ_Δ_ + Φ_F_ was 0.04 and 0.1 in THF and DMF, respectively), which has only one pyrazine unit, but its periphery is decorated by strongly electron withdrawing alkylsulfonyl groups.

**Figure 6:**
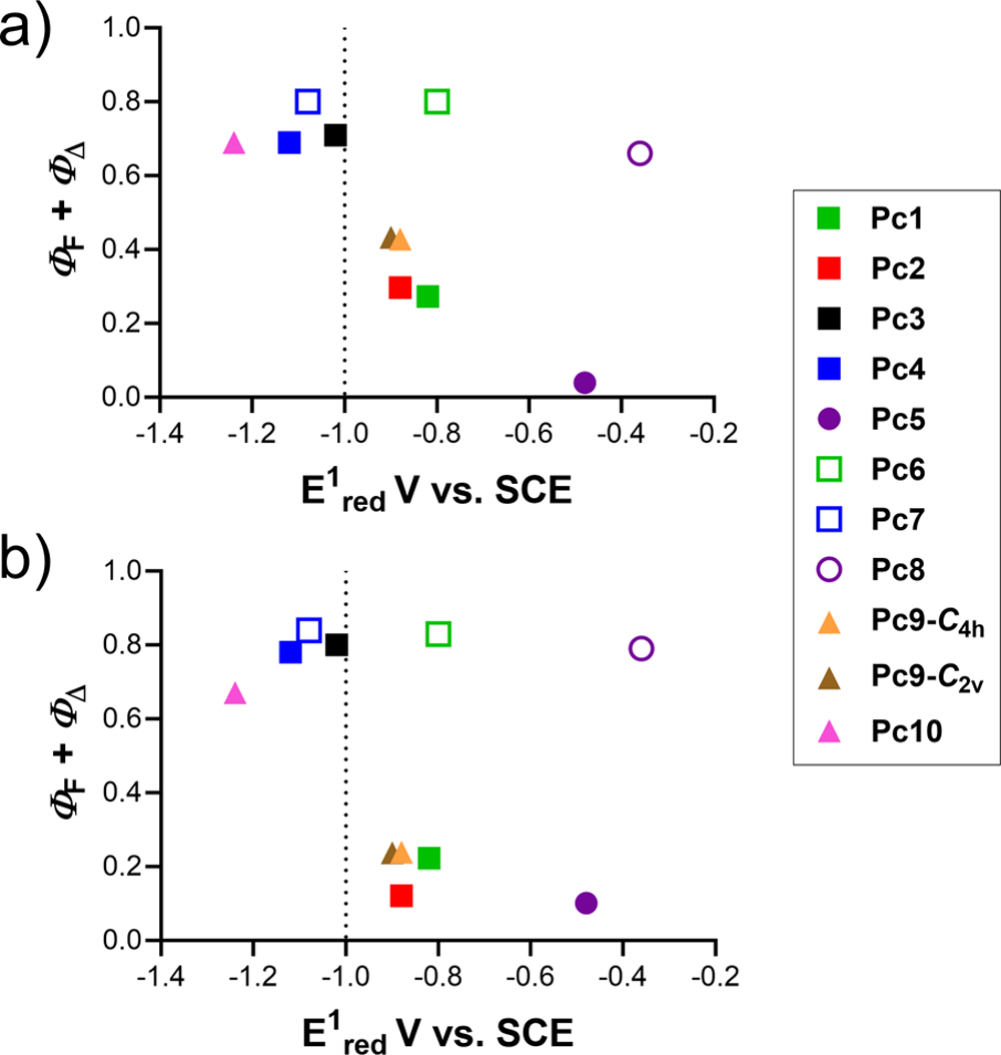
Relationships between ICT efficiency (monitored as a decrease of sum of Φ_F_ + Φ_Δ_ in THF (a) and DMF (b)) and the first reduction potential (*E*_red_^1^, in THF). Empty squares = controls without a donor for ICT; full squares = one donor for ICT; full triangles = four donor centers for ICT. The dotted line at −1.0 V vs SCE indicates the threshold where more negative values of *E*_red_^1^ lead to ICT inefficiency.

Correlation of the photophysical data with *E*_red_^1^ values, which illustrates the ability of a macrocycle to accept electrons, revealed that ICT efficiency is clearly proportional to the reduction potential of a given compound with an obvious threshold up to approximately −1.0 V vs SCE (Figure 6). Thus, weak or almost no ICT proceeds for derivatives having *E*_red_^1^ more negative than −1.0 V vs SCE (i.e., **Pc3**, **Pc4**, and **Pc10**), whereas ICT efficiency strengthens substantially in the series of **Pc9**-***C***_**2v**_ ∼ **Pc9**-***C***_**4*h***_ < **Pc2** < **Pc1** < **Pc5** in accordance with the increase in their *E*_red_^1^ values from −0.90 V vs SCE for **Pc9-*****C***_**2v**_ up to −0.48 V vs SCE for **Pc5**. The disclosed dependence showed that the first reduction potential is a helpful tool in prediction of the efficiency of ICT.

## Conclusion

Series of zinc(II) Pcs and their aza analogues with benzenes replaced by pyrazines have been prepared and studied from spectral, electrochemical, and photophysical points of view to reveal the effect of a macrocyclic core on electron-accepting properties and ICT efficiency. Interestingly, in the case of **Pc9** prepared by cyclotetramerization of 5-(bis(2-methoxyethyl)amino)-6-(pentan-3-ylsulfanyl)pyrazine-2,3-dicarbonitrile, we succeeded in rarely seen separation of constitutional isomers of *C*_4*h*_ and *C*_2*v*_ types. Electrochemical analysis revealed a linear dependence between *E*_red_^1^ values and a number of pyrazine units in the macrocycle, indicating that aza analogues of Pcs have stronger electron-accepting properties than parent Pcs. The electron-deficient character of a macrocycle can be even more strongly induced by introduction of electron-withdrawing groups such as alkylsulfonyl. Electron-accepting properties of given macrocycles correlated well with obtained photophysical data since the sum of Φ_Δ_ + Φ_F_ (i.e., sum of competitive relaxation pathways to ICT) decreased substantially with less negative *E*_red_^1^ values, irrespective of the number of ICT donors on the periphery. In terms of ICT efficiency, the threshold *E*_red_^1^ = −1.0 V vs SCE seems to be decisive, because macrocycles having more negative *E*_red_^1^ values failed to induce efficient ICT. Importantly, ICT efficiency was shown to correlate well with the decrease in Φ_F_ and τ_F_ in more polar solvents; however, the Lippert–Mataga plot indicated that differences in dipole moments in the ground and excited states do not correlate with ICT efficiency and rather reflect local electron distribution due to different geometry and/or electronic effect of substituents.

The results of this project unequivocally proved that the electron-accepting properties of the core can be easily tuned by the suitable design of the macrocycle and that the *E*_red_^1^ value can be used as a tool to assess the potential of a given macrocycle in applications based on donor–acceptor systems in Pcs and related compounds. This may be especially important in development of novel dye-sensitized solar cells, fluorescence sensors, catalysts, or molecular electronic devices, where Pcs serve as key components as electron acceptors.

## Supporting Information Available

Details on the synthesis of precursors and target macrocycles, electrochemical measurements, and determination of Φ_Δ_ and Φ_F_; NMR spectra of prepared compounds; UV–vis and fluorescence spectra of target macrocycles; electrochemical data (PDF)
