# Supplementary material for: Tuning Electron-Accepting Properties of Phthalocyanines for Charge Transfer Processes
Source: Inorg Chem. 2024 Apr 29;63(19):8799–806. doi: 10.1021/acs.inorgchem.4c00527 (PMC11094797; doi:10.1021/acs.inorgchem.4c00527)
Supplement: Supplementary file 1 — ic4c00527_si_001.pdf [file ic4c00527_si_001.pdf]

Supporting Information

Tuning electron-accepting properties of phthalocyanines for charge transfer processes

Stefan Bednarik, Jiri Demuth, Jakub Kernal, Miroslav Miletin, Petr Zimcik and Veronika Novakova

Faculty of Pharmacy in Hradec Kralove, Charles University, Ak. Heyrovskeho 1203, Hradec Kralove, 500 05 Czech Republic

Content

Experimental section **S2**
General S2
Synthesis of precursors S2
Synthesis of macrocycles via Linstead method S4
Synthesis of zinc macrocycles by template method S7
Electrochemical measurements S7
Determination of fluorescence quantum yields S7
Determination of fluorescence lifetimes S8
Determination of quantum yields of singlet oxygen production S8
NMR spectra **S9**
Spectral properties of studied macrocycles **S34**
Cyclic voltammograms and square wave voltammograms **S36**
Fluorescence intensity decay curves of Pc1 .... **S38**
References **S38**

Experimental section

General

All chemical reagents were purchased from certified suppliers (Sigma-Aldrich, TCI, Acros Organic, Fluorochem) and used as received. All the organic solvents used in the synthesis were of analytical grade. Thin-layer chromatography was performed on Merck aluminum sheets coated with silica gel 60 F254. Merck Kieselgel 60 (0.040–0.063 mm) was used for column chromatography. The infrared spectra were measured on a Nicolet 6700 spectrometer in the ATR mode. The ^{1}H and ^{13}C NMR spectra were recorded on a Varian Mercury Vx BB 300, VNMR S500 NMR spectrometer or Jeol JNM-ECZ600R. The chemical shifts are reported as δ values in ppm and are indirectly referenced to $Si(CH_3)_4$ via the signal from the solvent. J values are given in Hz. The UV–vis spectra were recorded using a Shimadzu UV-2600 spectrophotometer (Shimadzu, Kyoto, Japan). Fluorescence spectra were recorded using FS5 spectrofluorometer (Edinburgh Instruments, Edinburgh, UK). UHPLC system Acquity UPLC I-class (Waters, Millford, USA) coupled to a high-resolution mass spectrometer (HRMS) Synapt G2Si (Waters, Manchester, UK) based on Q-TOF were used for HRMS spectra measurement. Chromatography was carried out using Acquity UPLC BEH C18 (2.1 x 50 mm, 1.7 µm) or Acquity UPLC BEH C4 (2.1 x 50 mm, 1.7 µm, 300 Å) columns for molecules up to or above 1200 m/z, respectively, using gradient elution with acetonitrile and 0.1% formic acid at flow-rate 0.4 ml/min. Electrospray ionization was operated in positive mode. The ESI spectra were recorded using leucine-enkefaline as a lock mass reference and sodium formate (in the range 50 - 1200 m/z) or sodium iodide (in the range 50 – 2000 m/z) for mass calibration.

Synthesis of precursors

Synthesis of 4-(bis(2-methoxyethyl)amino)-5-chlorophthalonitrile (1): 4,5-dichlorophthalonitrile (5 g, 25.38 mmol) and K_2CO_3 (21 g, 152.24 mmol) were added to dried flask under argon atmosphere. Then, anhydrous DMSO (40 mL) and bis(2-methoxylethyl)amine (16.90 g, 18.74 ml, 126.88 mmol) were added and entire reaction mixture was stirred at room temperature for 10 days under argon atmosphere. The reaction was monitored by TLC (hexane/ethyl acetate 1:1, R_f= 0.40). Then, the reaction mixture was diluted by water (250 mL) and product was extracted by ethyl acetate (3 × 100 mL). The organic phase was collected, dried over anhydrous Na_2SO_4, and the solvent was evaporated to dryness. Crude product was purified by column chromatography on silica using hexane/ethyl acetate 1:1 and after elution of non-reacted starting compound mobile phase was changed to hexane/ethyl acetate 2:1. Yield: 689 mg (9%) of yellow oil that solidified in time. ^{1}H NMR (500 MHz, $CDCl_3$) δ 7.66 (s, 1H), 7.51 (s, 1H), 3.59 (t, *J* = 5.3 Hz, 4H), 3.52 (t, *J* = 5.5 Hz, 4H), 3.29 (s, 6H). ^{13}C NMR (126 MHz, $CDCl_3$) δ 152.55, 135.98, 130.95, 126.72, 115.25, 115.02, 114.56, 106.84, 70.52, 58.90, 51.66. HR-MS (ESI): (m/z) calc. for $[M+H]^+$ 294.1004, found 294.1005.

Synthesis of 4-(bis(2-methoxyethyl)amino)-5-(pentan-3-ylsulfanyl)phthalonitrile (2): Pentane-3-thiol (611 mg, 736.14 µl; 5.86 mmol) and K_2CO_3 (1134 mg, 8.21 mmol) were added to the solution of compound **1** (689 mg, 2.35 mmol) in DMSO (50 mL) at 60 °C. The reaction mixture was stirred at 60 °C for 48 h. Then, it was diluted with water (300 mL). Product was extracted with ethyl acetate (3 × 100 mL), organic layers were collected, dried over anhydrous Na_2SO_4, and the solvents were evaporated to dryness. Crude product was isolated by column chromatography on silica using toluene/acetone 10:1 (R_f= 0.55) as the mobile phase. Yield: 738 mg (87%) as a yellow oil. ^{1}H NMR (500 MHz, $CDCl_3$) δ 7.51 (s, 1H), 7.42 (s, 1H), 3.50 (t, *J* = 4.5 Hz, 4H), 3.45 (t, *J* = 5.2 Hz, 4H), 3.27 (s, 6H), 3.21 (p, *J* = 6.3 Hz, 1H), 1.75 – 1.57 (m, 4H), 1.01 (t, *J* = 7.4 Hz, 6H). ^{13}C NMR (126 MHz, $CDCl_3$) δ 153.16, 140.82, 132.57, 126.46, 115.97, 115.94, 111.41, 108.53, 70.48, 58.73, 51.70, 51.62, 49.21, 26.21, 10.96. HR-MS (ESI): (m/z) calc. for $[M+H]^+$ 362.1897, found 362.1903.

Synthesis of 4-chloro-5-(pentan-3-ylsulfanyl)phthalonitrile (3): Pentane-3-thiol (1.09 g, 10.5 mmol) was added to 4,5-dichlorophthalonitrile (1.52 g, 7.7 mmol) in DMSO (10 mL). Then, anhydrous K_2CO_3 (2.00 g, 15 mmol) was added in 5 portions upon stirring and simultaneous sonicating at room temperature. After that, the reaction mixture was heated at 60 °C overnight. Water (150 mL) was added and product was extracted by ethyl acetate (3×50 mL), dried over anhydrous Na_2SO_4, and purified by column chromatography on silica with toluene as mobile phase. Product was recrystallized from methanol. Yield: 1.73 g (85%) as a pale yellow solid, mp = 125.7 – 126.8 °C; ^{1}H NMR (500 MHz, $CDCl_3$) δ 7.72 (s, 1H), 7.52 (s, 1H), 3.26 (p, *J* = 6.2 Hz, 1H), 1.86 – 1.70 (m, 4H), 1.07 (t, *J* =

7.4 Hz, 6H); ^{13}C NMR (126 MHz, $CDCl_3$) δ 146.95, 137.00, 133.40, 129.92, 114.85, 114.51, 114.25, 110.99, 49.96, 26.19, 11.01. IR (ATR): ν = 3085, 3069, 2968, 2928, 2877, 2230, 1570, 1525, 1459, 1380, 1350, 1306, 1281, 1265, 1222 cm^{-1}.

Synthesis of 4,5-bis(pentan-3-ylsulfanyl)phthalonitrile (4): Pentane-3-thiol (1.6 mL, 1.32 g, 12.74 mmol) and K_2CO_3 (2.81 g, 20.33 mmol) were added to 4,5-dichlorophthalonitrile (1 g, 5.08 mmol) in DMSO (25 mL) and the reaction mixture was stirred for 72 hours at room temperature. Reaction was monitored by TLC (hexane/chloroform 2:1, R_f= 0.25). Then, the reaction mixture was poured into water (200 mL) and product was extracted with chloroform (3 × 50 mL). The organic phase was dried over anhydrous Na_2SO_4, and the solvent was evaporated to dryness. Finally, the product was purified by recrystallization from methanol. Yield: 1.29 g (77%) of white solid, mp = 137 – 142°C; ^{1}H NMR (600 MHz, Acetone-d_6) δ 7.87 (s, 2H), 3,62 (p, *J* = 6.2 Hz, 2H), 1.82 – 1.65 (m, 8H), 1.01 (t, *J* = 0.7 Hz, 12H). ^{13}C NMR (151 MHz, Acetone-d_6) δ 144.70, 130.49, 115.68, 111.13, 50.28, 26.34, 10.39. IR (ATR): ν = 2968, 2933, 2875, 2855, 2230, 1562, 1489, 1380, 1342, 1271, 1226, 1109 cm^{-1}. HR-MS (ESI): (m/z) calc. for $[M+2MeOH+H]^+$ 397.1978; found 397.1985.

Synthesis of 4,5-bis(pentan-3-ylsulfonyl)phthalonitrile (5):

Method A: Method was adopted from literature.[1] Compound **4** (500 mg, 1.5 mmol) was dissolved in acetic acid (4.5 mL) at increased temperature. Then, 30% H_2O_2 (8.3 mL) was added slowly and the reaction mixture was refluxed for 2 hours. The reaction was monitored by TLC (dichloromethane, R_f= 0.70). Then, the reaction mixture was poured into distilled water (250 mL), precipitate was collected. Yield: 311 mg (52%) of white solid, mp = 209 – 215°C. ^{1}H NMR (500 MHz, $CDCl_3$) δ 8.63 (s, 2H), 4.07 – 4.01 (m, 2H), 1.89 – 1.77 (m, 8H), 1.05 (t, *J* = 7.5 Hz, 12H). ^{13}C NMR (126 MHz, $CDCl_3$) δ 144.37, 138.24, 138.01, 120.51, 113.19, 67.70, 20.06, 10.68. HR-MS (ESI): (m/z) calc. for $[M+2MeOH+H]^+$ 461.1774, found 461.1780.

Method B: Method was adopted from literature.[2] Compound **4** (500 mg, 1.5 mmol) was dissolved in dichloromethane (20 mL), *m*-CPBA (1.3 g, 7.53 mmol) was added, and the reaction mixture was stirred at room temperature for 18 hours. The reaction was monitored by TLC (dichloromethane, R_f= 0.70). Then, the reaction mixture was poured into distilled water (250 mL), precipitate was collected and purified by column chromatography on silica using hexane/diethyl ether 1:1 as the eluent. Yield: 437 mg (14 - 73%, see discussion) of a white solid. NMR analysis showed the identical data as listed at the Method A.

Synthesis of 5-(bis(2-methoxyethyl)amino)-6-chloropyrazine-2,3-dicarbonitrile (6): Solution of 5,6-dichloropyrazine-2,3-dicarbonitrile (2.08 g, 10.5 mmol) in THF (50 mL) was cooled down using ice/NaCl 1:3. Then, solution of bis(2-methoxylethyl)amine (2.67 g, 2.96 mL, 20.04 mmol) in THF (50 mL) was added dropwise and stirring continued for next 2h. Reaction was monitored by TLC (ethyl acetate/ hexane 1:1, R_f= 0.50). The reaction mixture was evaporated to dryness, deep brown oil was suspended in ethyl acetate (150 mL) and washed with water (3 × 50 mL) and brine (1 × 50 mL). The organic layer was collected, dried over anhydrous Na_2SO_4, and the solvent was evaporated to dryness. Crude product was purified by column chromatography on silica using ethyl acetate/hexane 1:1 as the mobile phase. Yield: 2.50 g (81%) of a yellow oil. ^{1}H NMR (500 MHz, $CDCl_3$) δ 4.08 (t, *J* = 5.3 Hz, 4H), 3.64 (t, *J* = 5.2 Hz, 4H), 3.33 (br s, 6H). ^{13}C NMR (126 MHz, $CDCl_3$) δ 152.00, 135.73, 129.02, 118.06, 113.06, 112.86, 70.63, 59.05, 51.80. HR-MS (ESI): (m/z) calc. for $[M+H]^+$ 296.0909, found 296.0912.

Synthesis of 5-(bis(2-methoxyethyl)amino)-6-(pentan-3-ylsulfanyl)pyrazine-2,3-dicarbonitrile (7): Solution of compound **6** (2.22 g, 7.51 mmol) in THF (50 mL) was added dropwise to a solution of pentane-3-thiol (858 mg, 0.95 mL, 8.25 mmol) in THF (50 mL) with 1M NaOH (9 mL). The reaction mixture was stirred for 2 hours at room temperature and monitored by TLC (hexane/ethyl acetate 2:1, R_f = 0.50). Organic solvent was evaporated, ethyl acetate (150 mL) was added and crude mixture was washed with water (3 × 50 mL). The organic layer was separated, dried over anhydrous Na_2SO_4, and the solvent was evaporated to dryness. Crude product was purified by column chromatography on silica using hexane/ethyl acetate 2:1 as the mobile phase. Yield: 1.75 g (64%) as a yellow oil. ^{1}H NMR (500 MHz, $CDCl_3$) δ 3.98 (t, *J* = 5.4 Hz, 4H), 3.85 (p, *J* = 6.4 Hz, 1H), 3.62 (t, *J* = 5.4 Hz, 4H), 3.33 (s, 6H), 1.84 – 1.64 (m, 4H). 1.01 (t, *J* = 7.4 Hz, 6H). ^{13}C NMR (126 MHz, $CDCl_3$) δ 152.55, 149.76, 124.96, 119.53, 114.53, 114.10, 70.92, 58.98, 51.00, 50.55, 26.49, 11.29. HR-MS (ESI): (m/z) calc. for $[M+H]^+$ 364.1802, found 364.1807.

Synthesis of 5,6-bis(pentan-3-ylsulfanyl)pyrazine-2,3-dicarbonitrile (8): 5,6-dichloropyrazine-2,3-dicarbonitrile (6.02 g, 30.25 mmol) in THF (100 mL) was added into solution of pentane-3-thiol (6.39 g, 8.39 mL, 66.9 mmol) in 1M NaOH (66 mL). The reaction mixture was stirred for 2 hours at room temperature. Reaction was monitored by TLC (hexane/toluene 2:1, R_f= 0.26). Organic solvent was evaporated, product was diluted by water (100 mL) and product was extracted with ethyl acetate (3 × 50 mL). The organic phase was collected, dried over anhydrous Na_2SO_4, and the solvent was evaporated to dryness. The crude product was purified by column chromatography on silica using hexane/toluene 2:1 as the mobile phase. Finally, product was recrystallized from methanol. Yield: 7.25 g (72%) of light-yellow solid, mp = 81.5 – 84.0°C. ^{1}H NMR (500 MHz, $CDCl_3$) δ 3.96 (p, *J* = 6.4 Hz, 2H), 1.89 – 1.70 (m, 8H), 1.03 (t, *J* = 7.4 Hz,12H). ^{13}C NMR (126 MHz, $CDCl_3$) δ 160.97, 125.68, 113.86, 50.53, 26.61, 11.19. IR (ATR): ν = 2967, 2938, 2876, 2233, 1480, 1383, 1289, 1158, 1141, 985 cm^{-1}. HR-MS (ESI): (m/z) calc. for $[M+H]^+$ 335.1359, found 335.1364 $[M+H]^+$.

Synthesis of macrocycles via Linstead method

General procedure for synthesis of metal-free macrocycles: Magnesium turnings (28 eq.) with few crystals of iodine were refluxed in freshly distilled anhydrous butanol (approx. 1 mL per 1 mmol of magnesium) until all magnesium was converted to magnesium butoxide (typically 3 h). The particular precursors **2** or **7** (1 eq.) and **4** or **8** (3 eq.) (particular amounts mentioned at each compound below) were added, and the reflux continued overnight. After that, the reaction mixture was concentrated, the methanol/water/acetic acid 10:10:1.5 (v/v) was added (approx. 3 mL per 1 mmol of magnesium) and the mixture was stirred at room temperature until residual magnesium butoxide was consumed. The dark green precipitate was collected by filtration, washed thoroughly with methanol/water/acetic acid 10:10:1.5 (v/v), then with water and air-dried. The crude mixture of magnesium congeners was directly converted to metal-free analogues by their dissolving in THF and adding of *p*-toluenesulfonic acid (10 eq. per 1 eq. of macrocycles). The mixture was stirred for 2 hours at room temperature. Then, the solution was concentrated, water was added, arisen precipitation was collected by filtration and air-dried to obtain mixture of metal-free congeners. The desired congener (of ABBB type) was isolated by column chromatography on silica. Mobile phases and yields are mentioned at each compound below.

Pc1-H: prepared by general procedure for metal-free TPyzPzs starting from compound **8** (502 mg, 1.50 mmol) and **7** (185 mg, 0.51 mmol); mobile phase: toluene/chloroform/THF 30:10:1 (product purified twice, R_f = 0.50). Yield: 122 mg (18%) of dark green solid. ^{1}H NMR (500 MHz, $CDCl_3$/pyridine-d_5 3:1) δ 5.19 – 5.09 (m, 5H), 5.08 – 5.01 (m, 2H), 4.63 (t, *J* = 5.7 Hz, 4H), 4.24 (t, *J* = 5.7 Hz, 4H), 3.68 (s, 6H), 2.50 – 2.32 (m, 28H), 1.67 – 1.55 (m, 42H), -1.06 (s, 2H). ^{13}C NMR (126 MHz, $CDCl_3$/pyridine-d_5 3:1) δ 160.59, 159.43, 157.04, 154.83, 145.20, 142.66, 142.41, 72.07, 59.52, 51.91, 50.46, 50.37, 50.17, 50.15, 50.10, 27.88, 27.84, 27.80, 12.37, 12.34, 12.25, 12.21. HR-MS (ESI): (m/z) calc. for $[M+H]^+$ 1368.5816, found 1368.5779 $[M+H]^+$.

Pc2-H: prepared by general procedure for metal-free TPyzPzs starting from compounds **8** (628 mg, 1.88 mmol) and **2** (226 mg, 0.63 mmol); purified twice - mobile phases: chloroform/toluene 5:1 and subsequently chloroform/THF 100:1 (R_f = 0.55). Yield: 131 mg (15%) of dark green solid. ^{1}H NMR (500 MHz, $CDCl_3$) δ 9.44 (s, 1H), 9.36 (s, 1H), 4.93 – 4.82 (m, 4H), 4.74 (dp, *J* = 19.0, 6.4 Hz, 2H), 3.85 (t, *J* = 6.1 Hz, 4H). 3.83 – 3.78 (m, 1H), 3.74 (t, *J* = 6.0 Hz, 4H), 3.39 (s, 6H), 2.24 – 2.12 (m, 24H), 2.07 – 1.99 (m, 4H), 1.46 – 1.33 (m, 36H), 1.28 (t, *J* = 7.4 Hz, 6H), -0.71 (s, 2H). ^{13}C NMR (126 MHz, $CDCl_3$) δ 159.71, 158.92, 158.79, 158.56, 158.39, 152.36, 144.97, 144.16, 142.85, 141.14, 121.81, 117.81, 71.07, 58.92, 53.73, 50.05, 49.95, 49.82, 49.74, 49.59, 48.99, 27.30, 27.27, 27.24, 26.80, 11.95, 11.93, 11.74, 11.57. HR-MS (ESI): (m/z) calc. for $[M+H]^+$ 1366.5911, found 1366.5902.

Pc3-H: prepared by general procedure for metal-free TPyzPzs starting from compound **4** (500 mg, 1.50 mmol) and **7** (182 mg, 0.50 mmol); mobile phase: toluene/chloroform/THF 40:10:1 (R_f = 0.40). Yield: 92 mg (13%). ^{1}H NMR (500 MHz, pyridine-d_5) δ 9.48 – 9.36 (m, 6H), 4.80 (p, *J* = 6.4 Hz, 1H), 4.52 (t, *J* = 5.8 Hz, 4H), 4.05 (t, *J* = 5.8 Hz, 4H), 3.91 (p, *J* = 6.2 Hz, 1H), 3.88 – 3.79 (m, 4H), 3.74 (p, *J* = 6.2 Hz, 1H), 3.41 (s, 6H), 2.27 – 2.14 (m, 4H), 2.06 – 1.91 (m, 24H), 1.44 (t, *J* = 7.4 Hz, 6H), 1.30 – 1.21 (m, 36H), -0.84 (s, 2H). ^{13}C NMR (126 MHz, pyridine-d_5) δ 156.68, 153.62, 142.48, 142.28, 141.98, 141.50, 141.33, 140.90, 71.56, 58.71, 51.35, 51.22, 51.17, 51.11, 51.08, 27.56, 27.03, 26.92, 12.20, 11.43.

Pc4-H: prepared by general procedure for metal-free TPyzPzs starting from compounds **4** (638 mg, 1.92 mmol) and **2** (231 mg, 0.64 mmol); mobile phase: chloroform/toluene/THF 50:50:1 (R_f = 0.40). Yield: 186 mg (21%) of dark green solid. ^{1}H NMR (600 MHz, $CDCl_3$) δ 9.26 (s, 1H), 9.24 (s, 1H), 9.21 (s, 1H), 9.20 (s, 1H), 9.19 (s, 2H), 9.17 (s, 1H), 9.15 (s, 1H), 3.86 (t, 4H), 3.82 – 3.72 (m, 11H), 3.42 (s, 6H), 2.06 – 2.00 (m, 28H), 1.33 – 1.27 (m, 42H), -0.29 (s, 2H). ^{13}C NMR (151 MHz, $CDCl_3$) δ 151.59, 141.59, 141.17, 141.09, 140.94, 140.84, 140.72, 140.28, 134.85, 133.31, 123.73, 122.84, 122.77, 122.61, 122.53, 122.31, 121.44, 117.10, 71.22, 58.89, 53.95, 51.53, 51.39, 51.37, 51.17, 48.92, 26.89, 26.86, 26.83, 26.81, 11.65, 11.57, 11.54, 11.48. HR-MS (ESI): (m/z) calc. for $[M+H]^+$ 1360.6196, found 1360.6193.

Pc6-H: symmetrical compound **Pc6-H** was isolated during the purification of asymmetrical macrocycle **Pc1-H** by column chromatography on silica (chloroform/toluene/THF, R_f = 0.60) 10:30:1. Yield: 161 mg (24%) of dark green solid. ^{1}H NMR (500 MHz, $CDCl_3$) δ 4.90 (p, *J* = 6.4 Hz, 8H), 2.22 – 2.15 (m, 32H), 1.38 (t, *J* = 7.3 Hz, 48H), -1.28 (s, 2H). ^{13}C NMR (126 MHz, $CDCl_3$) δ 159.71, 143.39, 129.03, 49.78, 27.27, 11.73.

Pc7-H: this symmetrical compound was isolated during synthesis of **Pc3-H** as the first eluted fraction during column chromatography on silica (mobile phase toluene/THF 50:1). Subsequently, product was purified by another column chromatography on silica using toluene/hexane 2:1 as a mobile phase (R_f = 0.55). Yield: 93 mg (14%) of dark green solid. ^{1}H NMR (500 MHz, $CDCl_3$/pyridine-*d*$_5$ 3:1) δ 9.26 – 8.73 (bs, 8H), 3.81 (p, *J* = 6.1 Hz, 8H), 2.12 (p, *J* = 7.3 Hz, 32H), 1.39 (t, *J* = 7.3 Hz, 48H), -1.59 (s, 2H). ^{13}C NMR (126 MHz, $CDCl_3$) δ 141.13, 51.32, 26.91, 11.70 (some of the aromatic signals were not shown in the spectrum).

General procedure for coordination of zinc(II) into the center of macrocycles: Metal-free tetrapyrazinoporphyrazine (1 eq.) and anhydrous zinc acetate (10 eq.) were refluxed in pyridine (approx. 1 mL per 4 µmol of tetrapyrazinoporphyrazine) for 1 h. Then solvent was evaporated, water was added, precipitate was collected by filtration and air-dried. The crude product was purified by column chromatography on silica. The mobile phases and obtained yields are mentioned at each compound below.

Pc1: prepared by general procedure for coordination of zinc(II) into the center of macrocycles starting from **Pc1-H** (57 mg, 41.64 µmol); mobile phase toluene/chloroform/THF 30:10:2.5 (R_f = 0.25). Yield: 24 mg (40%) of dark green solid. ^{1}H NMR (500 MHz, $CDCl_3$/pyridine-*d*$_5$ 3 : 1) δ 5.11 – 5.01 (m, 5H), 4.94 (qt, *J* = 6.5, 3.4 Hz, 2H), 4.46 (t, *J* = 5.8 Hz, 4H), 4.11 (t, *J* = 5.8 Hz, 4H), 3.56 (s, 6H), 2.40 – 2.25 (m, 28H), 1.57 – 1.46 (m, 42H). ^{13}C NMR (126 MHz, $CDCl_3$/pyridine-*d*$_5$ 3 : 1) δ 157.40, 157.33, 157.29, 155.49, 152.24, 151.33, 151.27, 150.70, 150.30, 150.15, 149.98, 149.88, 149.79, 144.59, 144.52, 144.47, 144.42, 144.39, 144.03, 142.32, 70.95, 58.27, 50.63, 49.24, 48.87, 48.85, 48.81, 48.67, 26.72, 26.68, 26.65, 11.21, 11.16, 11.08, 11.04. HR-MS (ESI): (m/z) calc. for $[M+H]^+$ 1430.4951, found 1430.4907. UV-vis (THF, 1 µM): λ_{max} (ε): 650 (227 160), 592 (36 590), 378 nm (147 410 $mol^{-1}dm^3cm^{-1}$).

Pc2: prepared by general procedure for coordination of zinc(II) into the center of macrocycles starting from **Pc2-H** (83 mg, 60.7 µmol); mobile phase: chloroform/THF 100:1 (R_f = 0.25). Yield: 62 mg (71%) of green solid. ^{1}H NMR (600 MHz, $CDCl_3$/pyridine-*d*$_5$ 3 : 1) δ 9.50 (s, 1H), 9.43 (s, 1H), 5.03 – 4.96 (m, 4H), 4.88 (p, *J* = 6.3 Hz, 1H), 4.83 (p, *J* = 6.4 Hz, 1H), 3.94 – 3.89 (m, 4H+1H), 3.82 (t, *J* = 6.1 Hz, 4H), 3.44 (s, 6H), 2.33 – 2.20 (m, 24H), 2.13 – 2.06 (m, 4H), 1.51 (t, *J* = 7.3 Hz, 6H), 1.48 (t, *J* = 7.3 Hz, 6H), 1.43 (td, *J* = 7.3, 2.4 Hz, 24H), 1.36 (t, *J* = 7.4 Hz, 6H). ^{13}C NMR (151 MHz, $CDCl_3$/pyridine-*d*$_5$ 3 : 1) δ 157.93, 157.84, 157.64, 157.49, 157.37, 157.13, 151.51, 151.40, 151.32, 145.11, 144.34, 144.27, 140.68, 136.42, 123.52, 121.57, 117.49, 70.97, 58.59, 53.71, 49.83, 49.58, 49.31, 49.25, 48.66, 27.15, 26.71, 11.75, 11.53, 11.37. HR-MS (ESI): (m/z) calc. for $[M+H]^+$ 1428.5046, found 1428.5044. UV-vis (THF, 1 µM) λ_{max} (ε): 670 (167 950), 659 (172 350), 599 (33 200), 374 nm (133 920 $mol^{-1}dm^3cm^{-1}$).

Pc3: prepared by general procedure for coordination of zinc(II) into the center of macrocycles starting from **Pc3-H** (92 mg, 67.50 µmol); mobile phase toluene/chloroform/THF 40:10:1 (R_f = 0.40). Yield: 17 mg (18%) of green solid. ^{1}H NMR (500 MHz, pyridine-*d*$_5$) δ 9.75 (s, 1H), 9.74 (s, 1H), 9.73 (s, 1H), 9.72 (s, 1H), 9.71 (s, 1H), 9.70 (s, 1H), 4.84 (p, *J* = 6.5 Hz, 1H), 4.40 (t, *J* = 5.8 Hz, 4H), 3.94 (t, *J* = 5.8 Hz, 4H), 3.93 – 3.90 (m, 1H), 3.88 – 3.81 (m, 4H), 3.71 (p, *J* = 6.1 Hz, 1H), 3.30 (s, 6H), 2.22 – 2.09 (m, 4H), 2.00 (p, *J* = 7.0 Hz, 24H), 1.97 – 1.86 (m, 20 H), 1.38

(t, J = 7.3 Hz, 6H), 1.25 (t, J = 7.3 Hz, 6H) 1.22 – 1.16 (m, 30H). ^{13}C NMR (126 MHz, pyridine-d_5) δ 156.38, 155.73, 155.53, 155.45, 155.14, 153.75, 153.53, 152.49, 143.96, 142.79, 141.43, 141.16, 141.03, 140.84, 140.71, 140.18, 137.38, 137.08, 136.88, 136.83, 136.60, 136.41, 71.49, 58.59, 51.42, 51.30, 51.26, 51.18, 49.54, 27.65, 27.06, 27.00, 12.10, 11.47, 11.40. HR-MS (ESI): (m/z) calc. for $[M+H]^+$ 1424,5236; found 1424,5219. UV-vis (THF, 1 µM): λ_{max} (ε) 692 (276 330), 623 (49 920), 369 nm (101 260 mol^{-1}dm^{3}cm^{-1}).

Pc4: prepared by general procedure for coordination of zinc(II) into the center of macrocycles starting from **Pc4-H** (141 mg, 100 mmol), mobile phase: chloroform/THF100:1 (R_f = 0.40). Yield: 94 mg (64%) of green solid. ^{1}H NMR (600 MHz, $CDCl_3$/pyridine-d_5 3:1) δ 9.54 (s, 1H), 9.51 – 9.48 (m, 5H), 9.43 (s, 1H), 9.39 (s, 1H), 3.92 (t, J = 5.9 Hz, 4H), 3.89 – 3.85 (m, 7H), 3.83 (t, J = 7.2 Hz, 4H), 3.45 (s, 6H), 2.13 – 2.06 (m, 28H), 1.39 – 1.34 (m, 42H). ^{13}C NMR (151 MHz, $CDCl_3$/pyridine-d_5 3 : 1) δ 154.39, 154.07, 153.62, 153.22, 153.13, 150.75, 149.54, 140.56, 139.82, 139.34, 136.35, 136.09, 135.90, 123.72, 121.25, 117.01, 71.03, 58.57, 53.88, 51.31, 51.23, 51.04, 48.62, 26.77, 26.74, 11.45, 11.35, 11.27. HR-MS (ESI): (m/z) calc. for $[M+H]^+$ 1422.5331, found 1422.5306. UV-vis (THF, 1 µM) λ_{max} (ε): 706 (318 520), 634 (54 700), 363 nm (93 250 mol^{-1}dm^{3}cm^{-1}).

Pc6: prepared by general procedure for zinc macrocycles starting from **Pc6-H** (156 mg, 116 µmol), mobile phase: toluene/chloroform/THF 20:10:1.5 (R_f = 0.90). Yield: 138 mg (84%) of dark green solid. ^{1}H NMR (500 MHz, $CDCl_3$/pyridine-d_5 3:1) δ 5.01 (p, J = 6.4 Hz, 8H), 2.27 (p, J = 7.3 Hz, 32H), 1.45 (t, J = 7.3 Hz, 48H), ^{13}C NMR (126 MHz, $CDCl_3$/pyridine-d_5 3:1) δ 158.02, 150.95, 145.03, 49.40, 27.17, 11.56. HR-MS (ESI): (m/z) calc. for $[M+H]^+$ 1401.4508, found 1401.4459. UV-vis (THF, 1 µM) λ_{max} (ε): 649 (299 580), 589 (38 920), 378 nm (154 770 mol^{-1}dm^{3}cm^{-1}).

Pc7: prepared by general procedure for coordination of zinc(II) into the center of macrocycles starting from **Pc7-H** (60 mg, 45 µmol), mobile phase toluene/chloroform/THF 40:5:2 (R_f = 0.50). Yield: 27 mg (43%) of dark green solid. ^{1}H NMR (300 MHz, $CDCl_3$/pyridine-d_5 3:1) δ 9.49 (s, 8H), 3.88 (p, J = 6.1 Hz, 8H), 2.11 (d, J = 6.5 Hz, 32H), 1.38 (t, J = 7.3 Hz, 48H). ^{13}C NMR (75 MHz, $CDCl_3$/pyridine-d_5 3:1) δ 153.86, 140.26, 136.41, 123.15, 51.55, 27.08, 11.72. IR (ATR): ν = 2964, 2932, 2874, 2577, 2360, 2342, 1597, 1458, 1405, 1371, 1331, 1299, 1279, 1209, 1114, 1086, 1067, 946 cm^{-1}. UV-vis (THF, 1 µM) λ_{max} (ε): 706 (280 110), 635 (46 530), 380 (95 980 mol^{-1}dm^{3}cm^{-1}).

Pc9: General procedure for metal-free macrocycles was employed using compound **7** (281 mg, 0.77 mmol) as the only precursor. Arisen mixture of metal-free constitutional isomers (258 mg) was subsequently refluxed with anhydrous zinc acetate (311 mg, 1.70 mmol mmol) in pyridine for 30 minutes. The mixture of isomers was purified by column chromatography on silica with toluene/pyridine 10:1 as the mobile phase, that led to successful separation of constitutional isomers c_{4h} (R_f = 0.40), c_{2v} (R_f = 0.20) and a fraction containing mixture of congeners. Yield: 14 mg of **Pc9-C$_{4h}$** (5%), 78 mg of **Pc9-C$_{2v}$** (29%) and 20 mg of their mixture (7%).

Pc9-C$_{4h}$: ^{1}H NMR (500 MHz, $CDCl_3$/pyridine-d_5 3:1) δ 4.76 (p, J = 6.4 Hz, 4H), 4.35 (t, J = 5.8 Hz, 16H), 4.03 (t, J = 5.8 Hz, 16H), 3.49 (s, 24H), 7.2 (hept, J = 7.3 Hz, 16H), 1.45 (t, J = 7.3 Hz, 24H). ^{13}C NMR (126 MHz, $CDCl_3$/pyridine-d_5 3 : 1) δ 155.85, 152.76, 150.94, 150.73, 144.13, 142.78, 71.46, 58.82, 51.14, 49.36, 27.22, 11.78. HR-MS (ESI): (m/z) calc. for $[M+H]^+$ 1517.6280, found 1517.6250. UV-vis (THF, 1 µM) λ_{max} (ε): 654 (188 610), 596 (31 310), 482 (28 920), 376 nm (115 340 mol^{-1}dm^{3}cm^{-1}).

Pc9-C$_{2v}$: ^{1}H NMR (500 MHz, $CDCl_3$/pyridine-d_5 3 : 1) δ 4.91 – 4.85 (m, 2H), 4.76 – 4.71 (m, 2H), 4.35 – 4.33 (m, 8H), 4.33 – 4.30 (m, 8H), 4.04 – 4.01 (m, 8H), 4.01 – 3.99 (m, 8H), 3.51 – 3.49 (m, 12H), 3.49 – 3.47 (m, 12H), 2.26 – 2.14 (m, 16H), 1.47 – 1.43 (m, 12H), 1.41 – 1.37 (m, 12H). ^{13}C NMR (126 MHz, $CDCl_3$/pyridine-d_5 3 : 1) δ 155.90, 153.01, 152.98, 152.93, 152.83, 152.78, 152.69, 151.31, 151.25, 151.08, 151.00, 150.87, 150.58, 150.54, 150.51, 150.45, 150.39, 144.29, 144.23, 144.07, 144.00, 143.95, 143.00, 142.96, 142.85, 142.80, 142.75, 71.47, 71.32, 58.83, 51.17, 51.13, 51.04, 50.99, 49.40, 49.35, 48.92, 48.87, 27.24, 27.21, 11.81, 11.61. HR-MS (ESI): (m/z) calc. for $[M+H]^+$ 1517.6280, found 1517.6288. UV-vis (THF, 1 µM) λ_{max} (ε): 654 (201 030), 596 (34 570), 483 (31 400), 375 nm (125 780 mol^{-1}dm^{3}cm^{-1}).

Pc10: This compound was prepared by general procedure for metal-free macrocycles starting from compound **2** (145 mg, 0.40 mmol) as the only precursor. The mixture of metal-free isomers (115 mg) was without purification treated with anhydrous zinc acetate (145 mg, 0.79 mmol) according to general procedure for zinc TPyzPzs; mobile phase toluene/pyridine 10:1 (R_f = 0.50). Yield: 114 mg (75%) as a mixture of constitutional isomers. ^{1}H NMR (600

MHz; pyridine-d_5) δ 9.78 – 9.70 (m, 8H), 3.90 – 3.83 (m, 12H), 3.80 – 3.74 (m, 4H), 3.73 – 3.64 (m, 16H), 3.26 – 3.18 (m, 16H), 3.12 (br s, 12H), 1.96 – 1.88 (8H), 1.84 – 1.74 (m, 8H), 1.79 (br s, 8H), 1.24 – 1.17 (m, 12H), 1.14 – 1.07 (m, 12H). ^{13}C NMR (151 MHz, pyridine-d_5) δ 154.48, 154.18, 154.14, 151.97, 151.79, 150.07, 139.11, 139.07, 138.77, 136.878, 136.83, 136.72, 136.66, 135.35, 123.34, 122.30, 122.23, 117.85, 117.74, 117.68, 117.56, 71.23, 70.98, 58.41, 58.30, 53.86, 53.79, 53.62, 53.54, 48.75, 48.69, 48.64, 48.58, 27.02, 26.96, 11.50, 11.29. HR-MS (ESI): (m/z) calc. for $[M+H]^+$ 1509.6660, found 1509.6614.UV-vis (THF, 1 µM) λ_{max} (ε): 703 (196 360), 633 (33 620), 359 nm (57 890 mol^{-1}dm^{3}cm^{-1}).

Synthesis of zinc macrocycles by template method

Pc5: Compounds **5** (196 mg, 494 µmol) and **7** (59 mg, 162 µmol) and anhydrous zinc acetate (120 mg, 654 µmol) were placed into Schlenk flask and filled with argon atmosphere. Then, anhydrous pyridine (10 mL) was added, and entire reaction mixture was stirred at 115 °C for 18 hours protected from sunlight by aluminum foil. The solvent was evaporated, water (20 mL) was added, dark green precipitate was collected by filtration and purified twice by column chromatography using chloroform/THF 40:1 (R_f= 0.70) followed by diethyl-ether/THF 40:1 (R_f= 0.80) as mobile phases and then another column chromatography with diethyl-ether/THF 40:1 as the mobile phase. Yield: 13 mg (5%) of dark blue solid. ^{1}H NMR (600 MHz, $CDCl_3$/pyridine-d_5 3:1) δ 10.58 (s, 6H), 4.68 – 4.65 (m, 1H), 4.55 – 4.44 (m, 10H), 4.09 – 4.06 (m, 4H), 3.59 (s, 6H), 2.26 – 2.11 (m, 28H), 1.55 (t, J = 7.5 Hz, 6H), 1.20 (s, 36H). ^{13}C NMR (151 MHz, $CDCl_3$/pyridine-d_5 3:1) δ155.96, 155.65, 155.47, 155.37,155.09, 153.70, 153.44, 152.74, 148.63, 148.60, 143.16, 142.33, 141.04, 140.86, 140.76, 140.49, 139.95, 137.12, 136.82, 136.68, 136.63, 136.41, 136.20, 127.92, 123.77, 122.88, 122.83, 114.18, 71.75, 59.10, 51.67, 51.64, 51.59, 51.55, 51.47, 51.44, 51.42, 49.51, 27.54, 27.14, 27.08, 27.06, 12.20, 11.70, 11.67, 11.64. HR-MS (ESI): (m/z) calc. for $[M+H]^+$ 1616.4626, found 1616.4612. UV-vis (THF, 1 µM): λ_{max} (ε) 690 (112 470), 672 (110 010), 610 (28 310), 379 nm (62 300 mol^{-1}dm^{3}cm^{-1}).

Pc8: Compound **5** (245 mg, 0.62 mmol) and anhydrous zinc acetate (28 mg, 0.15 mmol) were placed into Schlenk flask and filled with argon atmosphere. Then, the mixture of *o-DCB/*anhydrous DMF 3:1 (4 mL) was added, and entire reaction mixture was stirred at 135 °C for 18 hours protected from sunlight by aluminum foil. The solvent was evaporated, extracted with DCM (3 × 100 mL) and purified by column chromatography on silica using toluene/pyridine 10:1 (R_f= 0.30). Yield: 214 mg (84%) of teal solid. ^{1}H NMR (600 MHz, $CDCl_3$/pyridine-d_5 3:1) δ 10.60 (br s, 8H), 4.53 (br s; 8H), 2.18 (br s, 32H), 1.21 (br s, 48H).^{13}C NMR (151 MHz; $CDCl_3$/pyridine-d_5 3:1) δ 154.22, 140.62, 129.75, 67.16, 20.29, 10.83. IR (ATR): ν =2971; 2939; 2881; 2360; 2342; 1489; 1458; 1290; 1137; 1099; 1079 cm^{-1}. HR-MS (ESI): (m/z) calc. for $[M+H]^+$ 1649,4074; found 1649.4026. UV-vis (THF, 1 µM): λ_{max} (ε) 684 (228 750), 617 (35 040), 378 nm (46 190 mol^{-1}dm^{3}cm^{-1}).

Electrochemical measurements

The electrochemical measurements (cyclic voltammetry, square wave voltammetry) were performed at room temperature using an Autolab PGSTAT101 potentiostat. Measurements were carried out with a three-electrode setup consisting of a Pt working electrode, a Pt counter electrode, and an Ag/AgCl reference electrode separated from the bulk solution by an integrated salt bridge. Detailed procedure was as follows: 0.1 M solution of tetrabutylammonium hexafluorophosphate in dry THF (5 mL) as a supporting electrolyte was added to the cell and bubbled with nitrogen for 5 minutes to remove residue of oxygen. Afterwards the appropriate compound (5-10 mg) was added, and bubbling continued for next 5 minutes. Halfwave potentials ($E_{1/2}$) were recorded from square wave voltammetry with potential step 5 mV and scan rate 100 mV/s. The obtained data listed in Table 1 were referenced to SCE with ferrocene as the internal standard ($E_{1/2}$ (Fc/Fc^+) = 0.56 V/SCE[3]).

Determination of fluorescence quantum yields

Fluorescence quantum yields (Φ_F) were determined in THF and DMF by a comparative method using unsubstituted zinc phthalocyanine (ZnPc) as a reference (Φ_F = 0.32 in THF[4]). In the case of **Pc1**, **Pc6** and **Pc9-C*2v*,** Φ_F were determined also in dioxane, pyridine, DMSO, and acetonitrile, tetrachloromethane, toluene, anisole, chlorobenzene, ethyl acetate employing the same method. Pyridine (0.1%v/v) was added to non-coordinating solvents (tetrachloromethane, toluene, anisole, chlorobenzene, ethyl acetate) to hinder aggregation (*i.e.*, formation of J-dimers). Both reference and sample were excited at 601 nm (for **Pc1**, **Pc2**, **Pc6**), 623 nm (for **Pc5**,

Pc9) and 638 nm (for **Pc3**, **Pc4**, **Pc7**, **Pc8**, **Pc10**). Absorbance at Q band maximum was kept below 0.1 to preclude inner filter effect. Φ_F was calculated using following Equation:

$$\Phi_F^S = \Phi_F^R \left(\frac{F^S}{F^R}\right)\left(\frac{1-10^{-A^R}}{1-10^{-A^S}}\right)\left(\frac{n^S}{n^R}\right)^2$$

, where *F* is the integrated area under the emission spectrum, *A* is the absorbance at the excitation wavelength, and *n* is the refractive index of the solvent. Superscripts *R* and *S* correspond to the reference and sample, respectively. All experiments were performed three times, and the data represent a mean of these three experiments. Estimated error ± 15%.

Determination of fluorescence lifetimes

Fluorescence lifetimes were determined for **Pc1**, **Pc6** and **Pc9-C_{2v}** in dioxane, THF, pyridine, DMSO, DMF and acetonitrile, tetrachloromethane, toluene, anisole, chlorobenzene, ethyl acetate using FLS-1000 (Edinburgh Instruments) with picosecond diode laser HPL-655 (λ_{ex} = 653.9 nm; 25 ns pulse period) as excitation source. Pyridine (0.1%v/v) was added to non-coordinating solvents (tetrachloromethane, toluene, anisole, chlorobenzene, ethyl acetate) to hinder aggregation (*i.e.*, formation of J-dimers).

Determination of quantum yields of singlet oxygen production

Singlet oxygen quantum yields (Φ_Δ) were determined in THF and DMF according to a previously described method[5] using the decomposition of 1,3-diphenylisobenzofuran (DPBF) and unsubstituted zinc phthalocyanine (ZnPc) as the reference ($\Phi_{\Delta(THF)}$ = 0.53[6], $\Phi_{\Delta(DMF)}$ = 0.56[7]). In detail, the procedure was as follows: 2.5 mL of a stock solution of DPBF in THF (or DMF) (5×10^{-5} M) was transferred into a 10 × 10 mm quartz optical cell and bubbled with oxygen for 1 min. Defined amount of concentrated stock solution of the sample in THF (usually 20 µL) was added. Absorbance of the final solution in Q-band maximum was always about 0.1. The solution was stirred and irradiated for defined times using a xenon lamp (100 W, ozone free XE DC short arc lamp, Newport). Incident light was filtered through a water filter (6 cm) and cutoff filter OG530 to remove heat and light under 523 nm, respectively. Decrease of DPBF in solution with irradiation time was monitored at 413 or 414 nm for THF or DMF, respectively. The Φ_Δ of samples was calculated using following Equation:

$$\Phi_\Delta^S = \Phi_\Delta^R \frac{k^S I_{aT}^R}{k^R I_{aT}^S}$$

, where *k* is a slope of the plot of the dependence of $ln(A_0/A_t)$ on irradiation time *t*, with A_0 and A_t being the absorbances of the DPBF at 413 nm (or 414 nm) before irradiation and after irradiation time *t*, respectively. I_aT is a total amount of light absorbed by the sample or reference. Superscripts *R* and *S* indicate reference and sample, respectively. I_aT is calculated as a sum of intensities of the absorbed light I_a at wavelengths from 523 to 850 nm (step 0.5 nm). Light under 523 nm is completely filtered off by OG530 filter and light above 850 nm is not absorbed by the studied compounds. I_a at given wavelength is calculated using Beer's law by following Equation:

$$I_a = I_0(1 - e^{-2.3A})$$

, where I_0 is a transmittance of the filter at the given wavelength and *A* absorbance of the sample or reference at this wavelength. All experiments were performed three times and data presented in the paper represent a mean of these three experiments. Estimated error ±10%.

NMR spectra

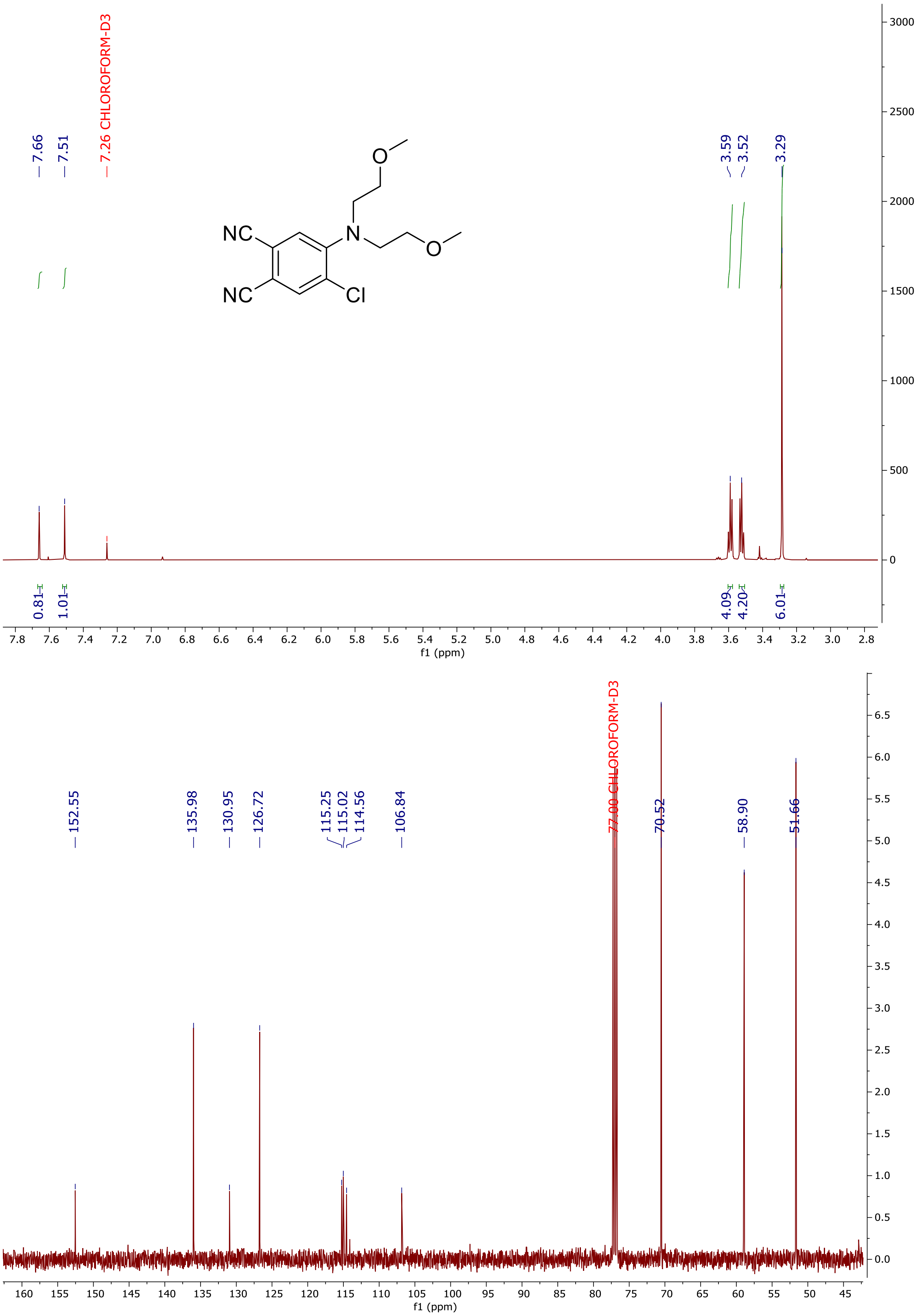

Figure S1: ^{1}H NMR (500 MHz, $CDCl_3$) and ^{13}C NMR (126 MHz, $CDCl_3$) of compound **1**.

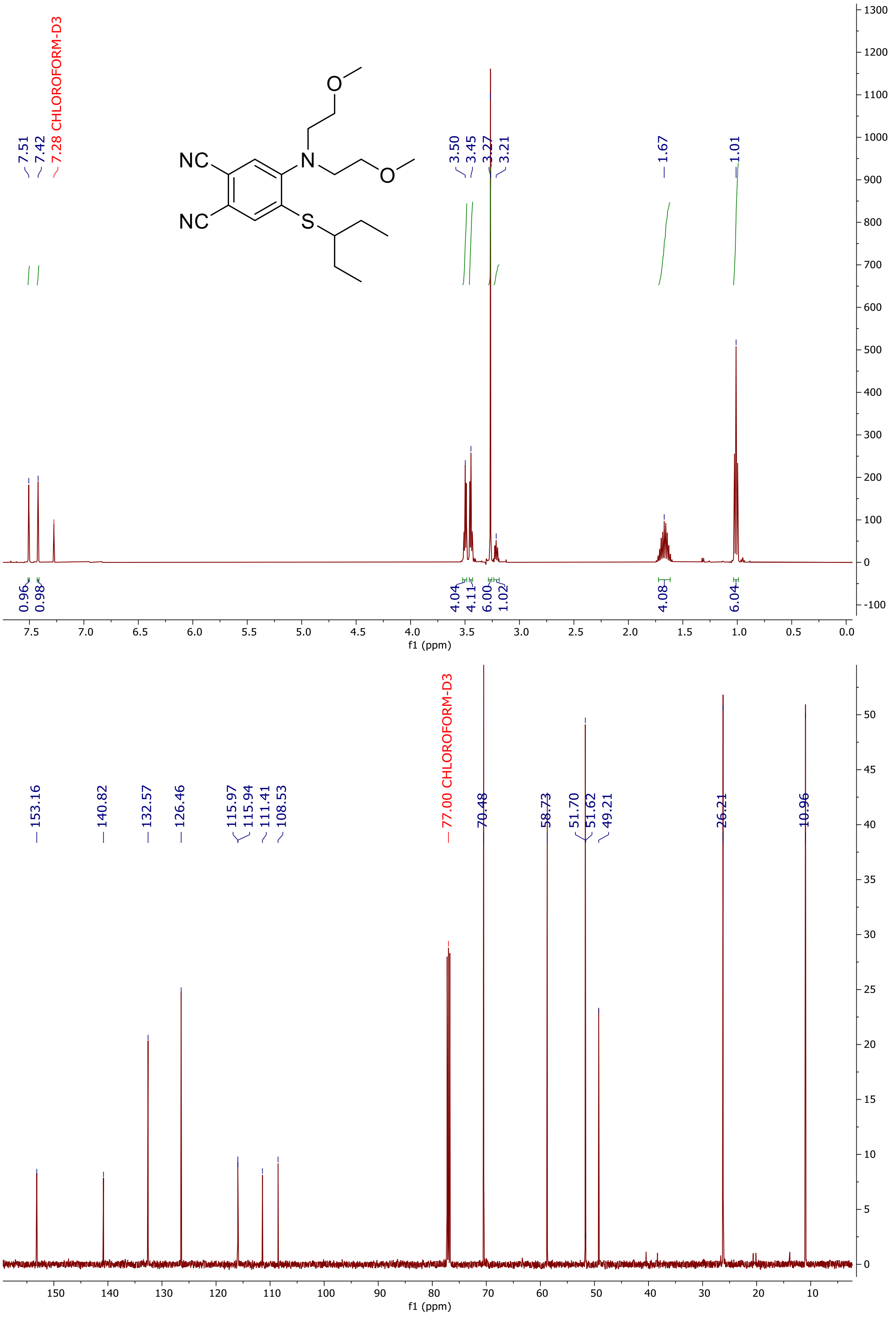

Figure S2: ^{1}H NMR (500 MHz, $CDCl_3$) and ^{13}C NMR (126 MHz, $CDCl_3$) of compound **2**.

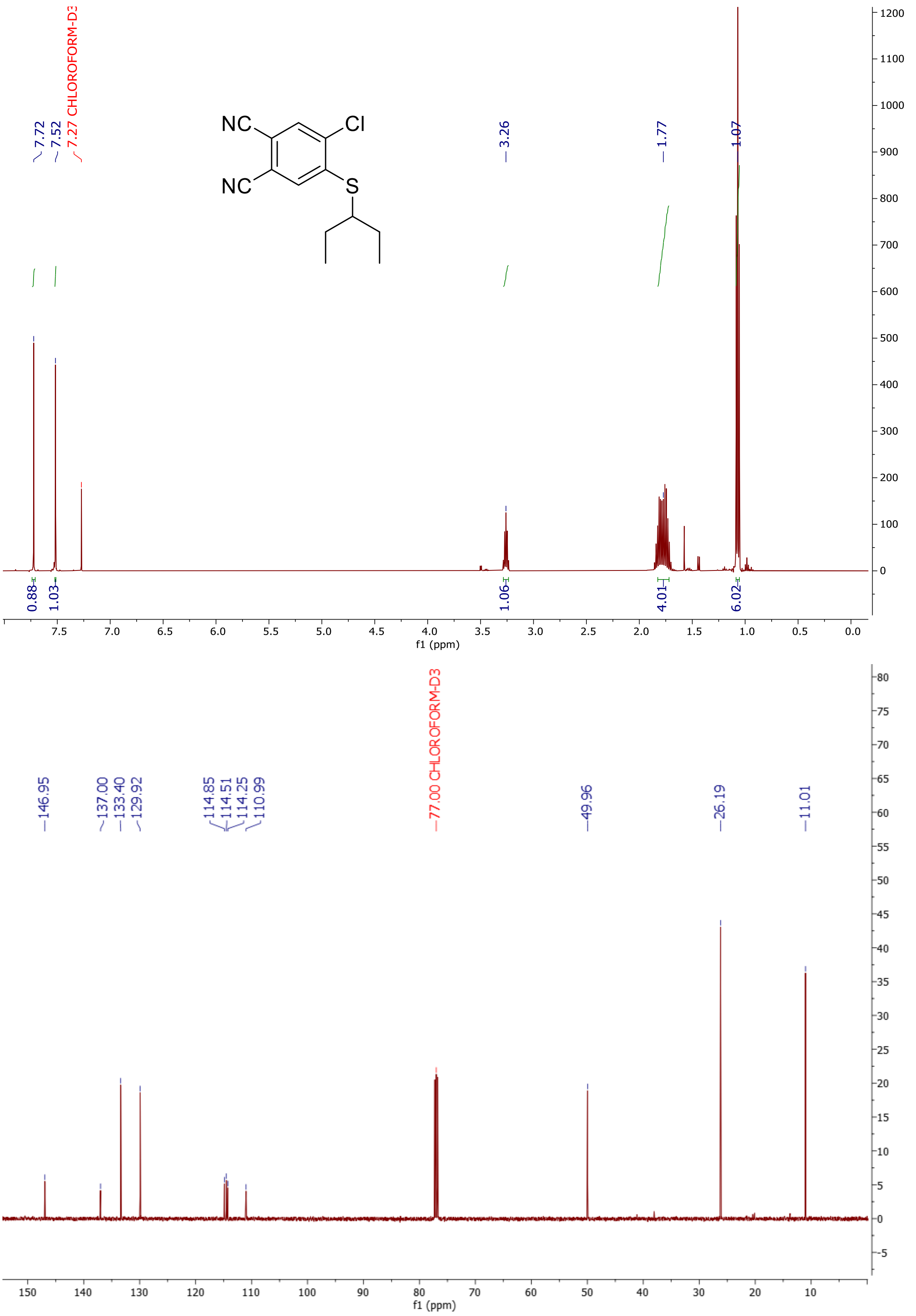

Figure S3: ^{1}H NMR (500 MHz, $CDCl_3$) and ^{13}C NMR (126 MHz, $CDCl_3$) of compound **3**.

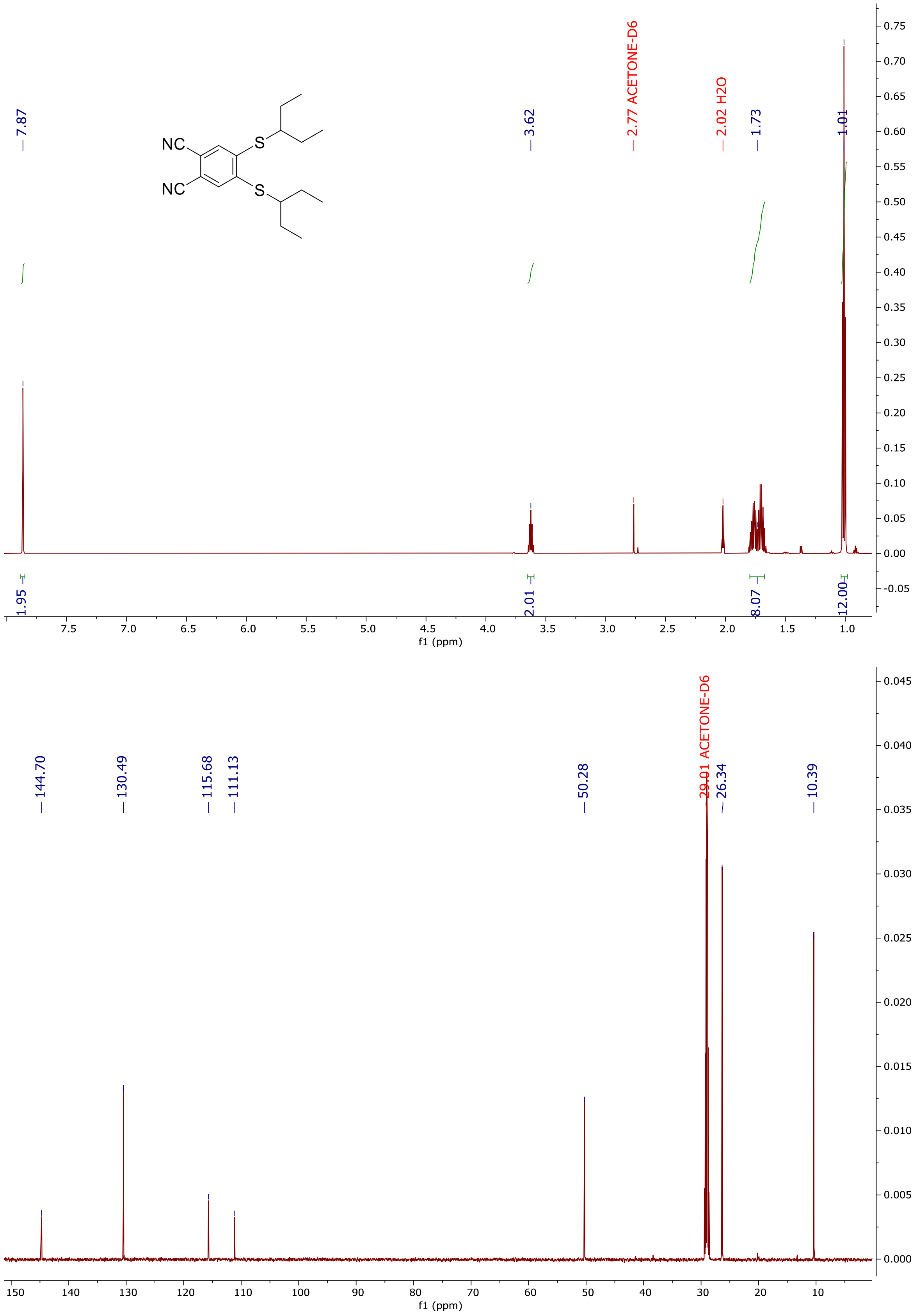

Figure S4: ^{1}H NMR (600 MHz, acetone-*d*6) and ^{13}C NMR (151 MHz, acetone-*d*6) of compound **4**.

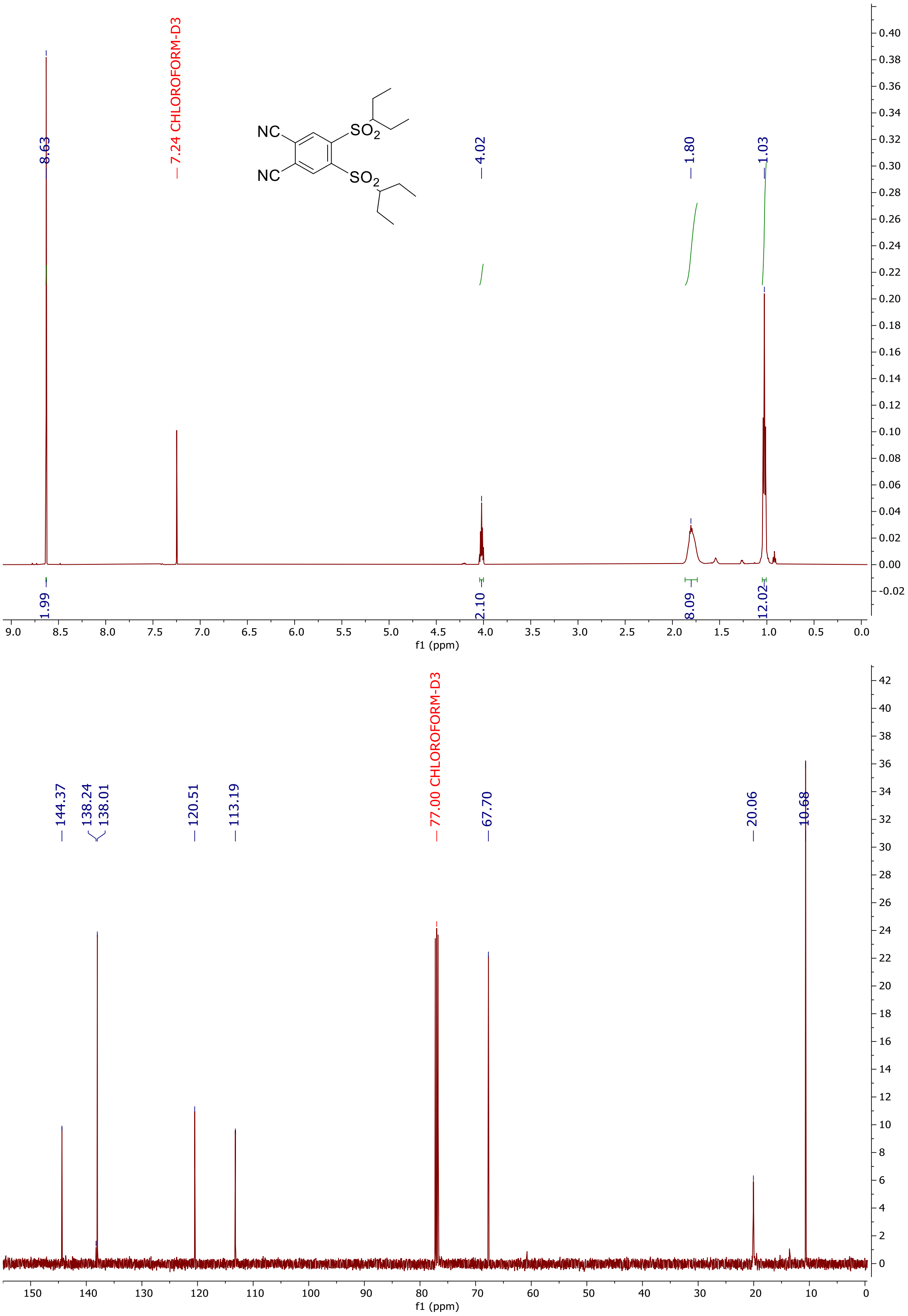

Figure S5: ^{1}H NMR (500 MHz, $CDCl_3$) and ^{13}C NMR (126 MHz, $CDCl_3$) of compound **5**.

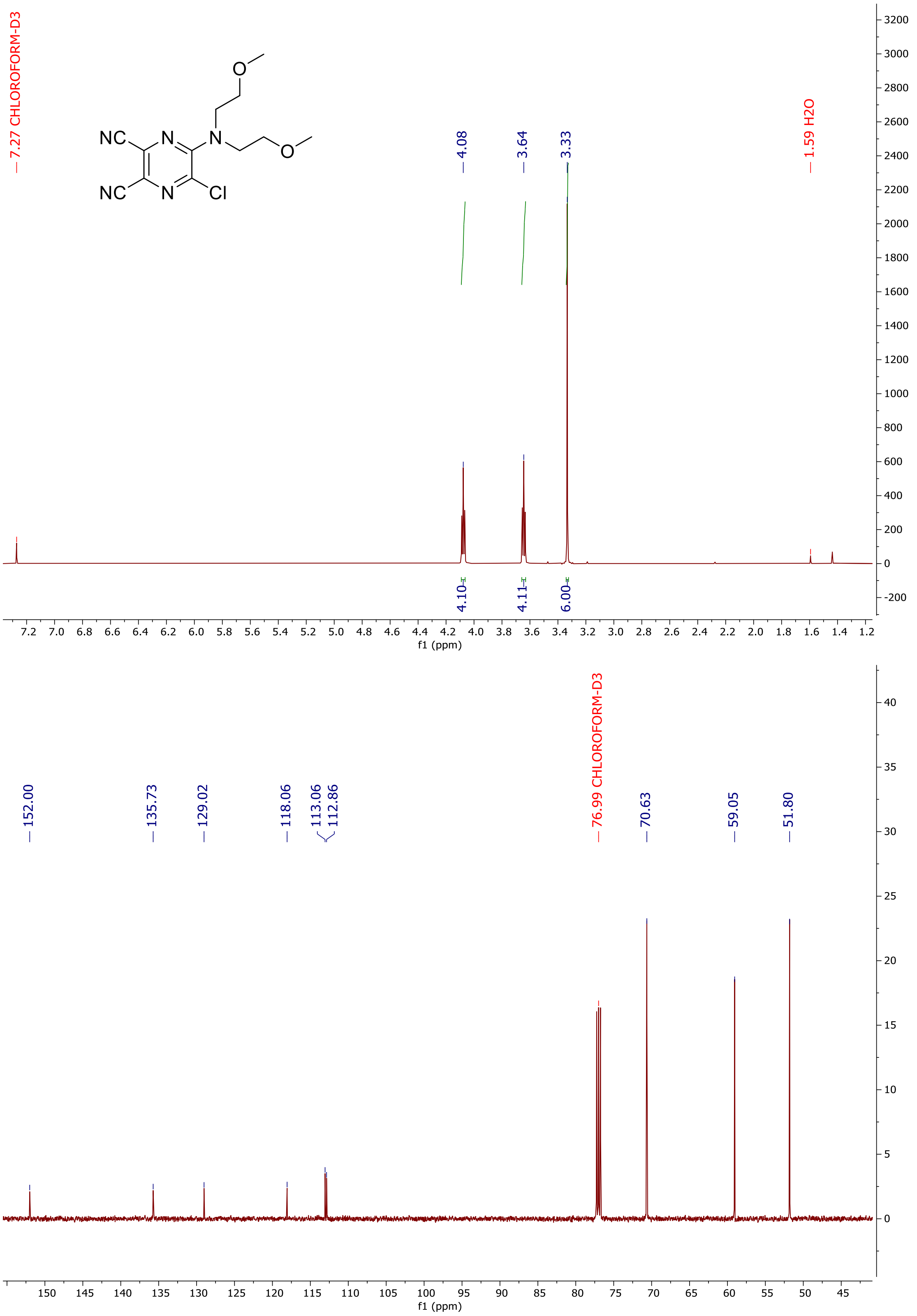

Figure S6: ^{1}H NMR (500 MHz, $CDCl_3$) and ^{13}C NMR (126 MHz, $CDCl_3$) of compound **6**.

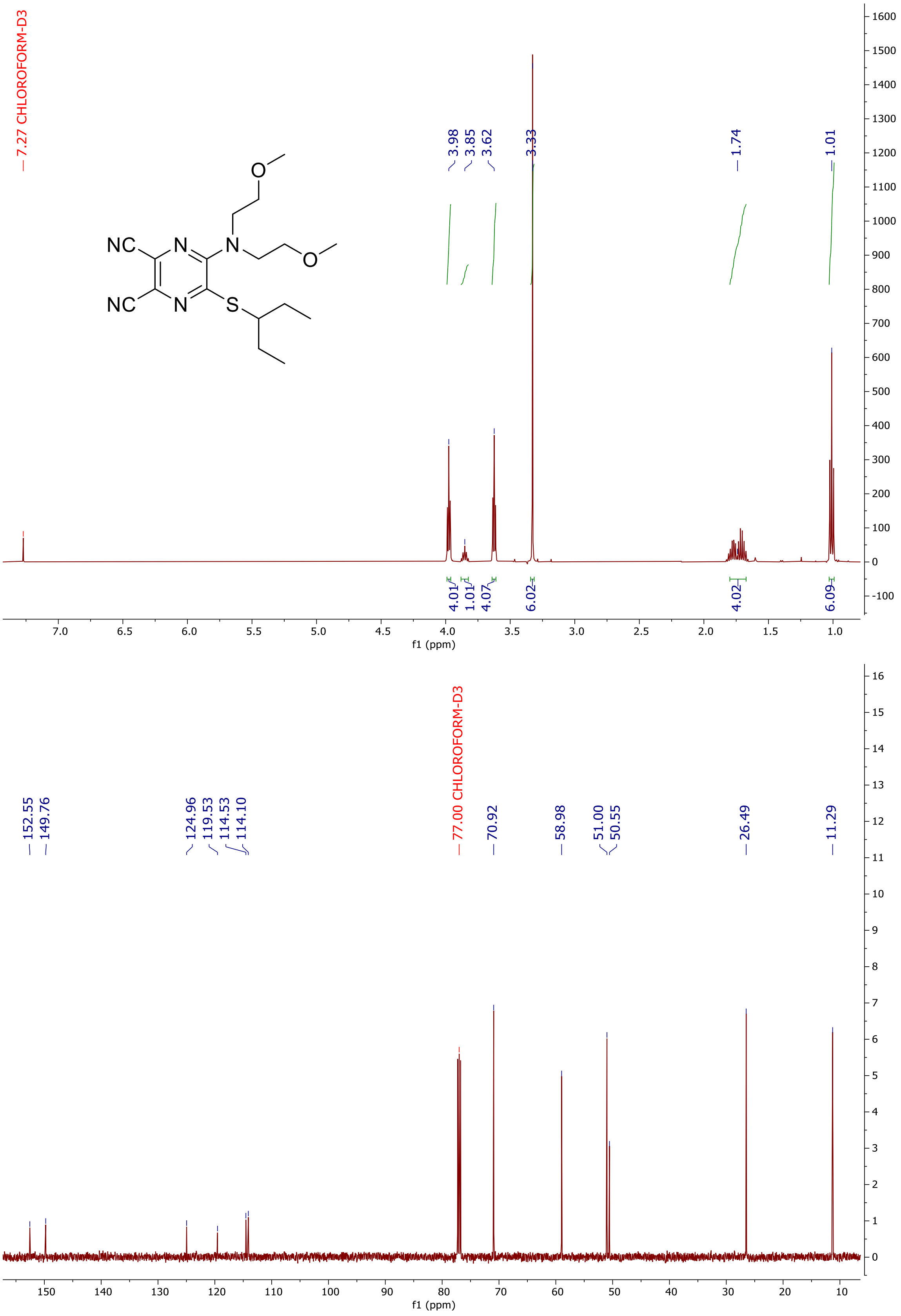

Figure S7: ^{1}H NMR (500 MHz, $CDCl_3$) and ^{13}C NMR (126 MHz, $CDCl_3$) of compound **7**.

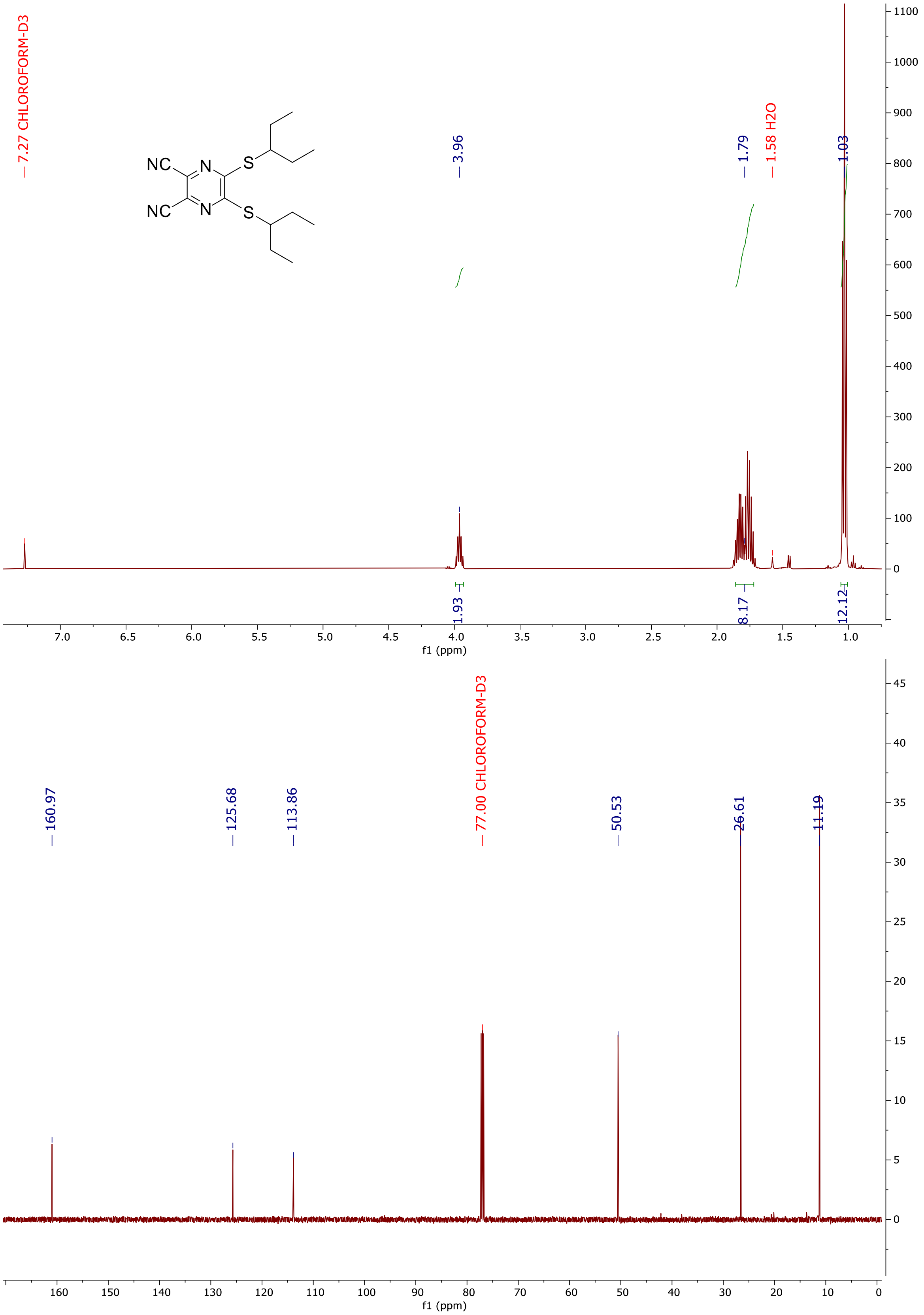

Figure S8: ^{1}H NMR (500 MHz, $CDCl_3$) and ^{13}C NMR (126 MHz, $CDCl_3$) of compound **8**.

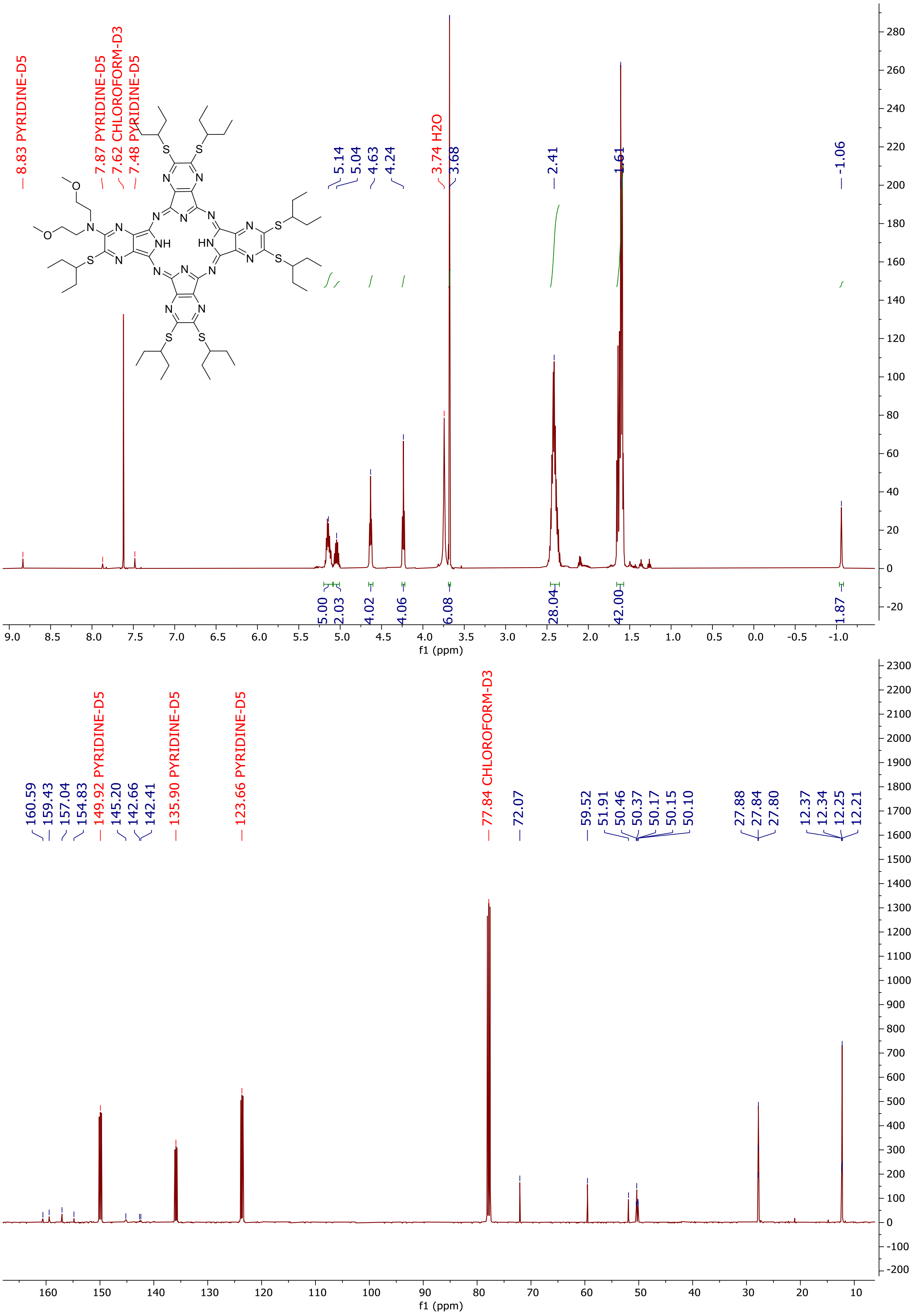

Figure S9: ^{1}H NMR (500 MHz, $CDCl_3$/pyridine-*d*$_5$ 3:1) and ^{13}C NMR (126 MHz, $CDCl_3$/ pyridine-*d*$_5$ 3:1) of **Pc1-H**.

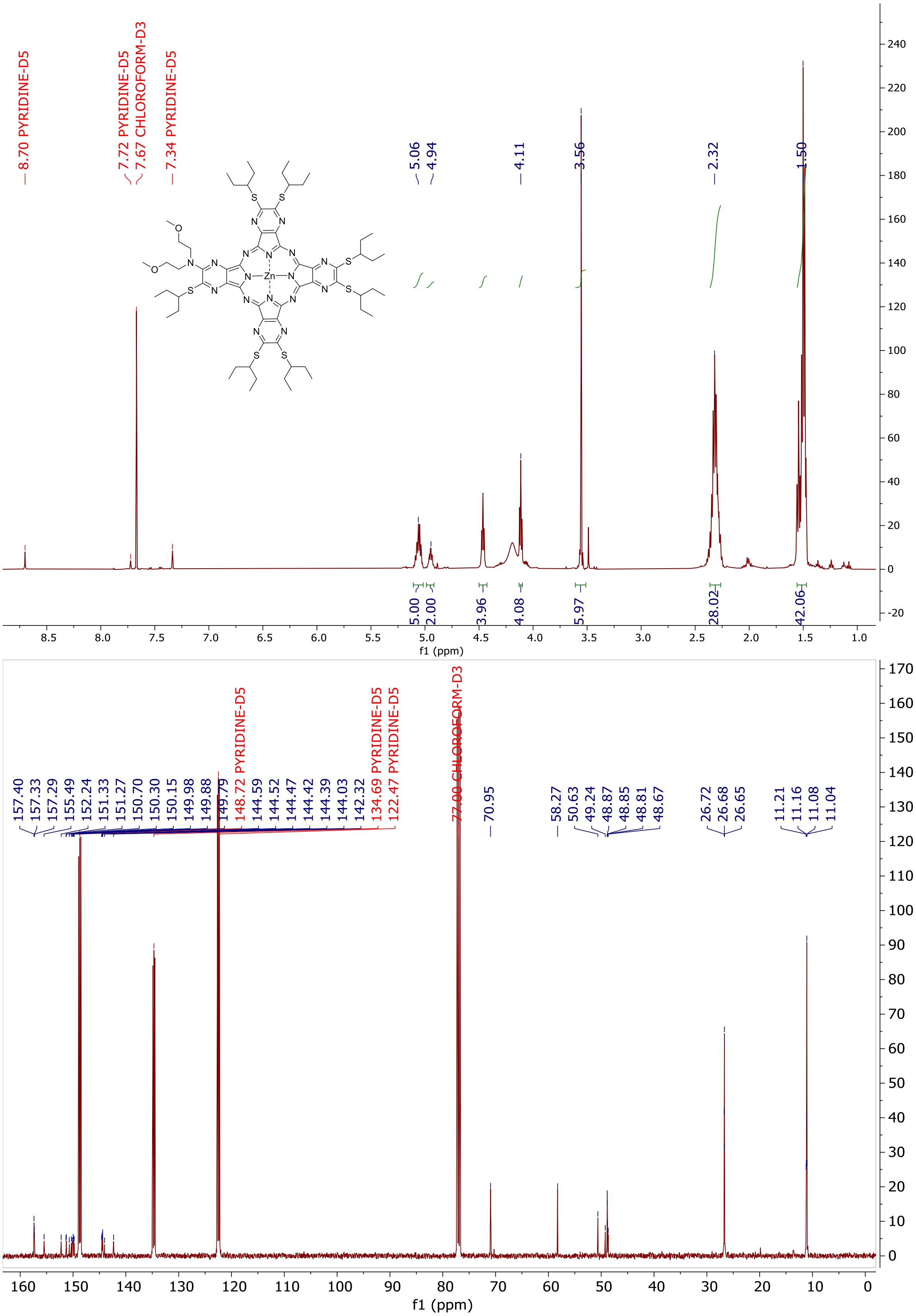

Figure S10: ^{1}H NMR (500 MHz, $CDCl_3$/pyridine-d_5 3:1) and ^{13}C NMR (126 MHz, $CDCl_3$/ pyridine-d_5 3:1) of **Pc1**.

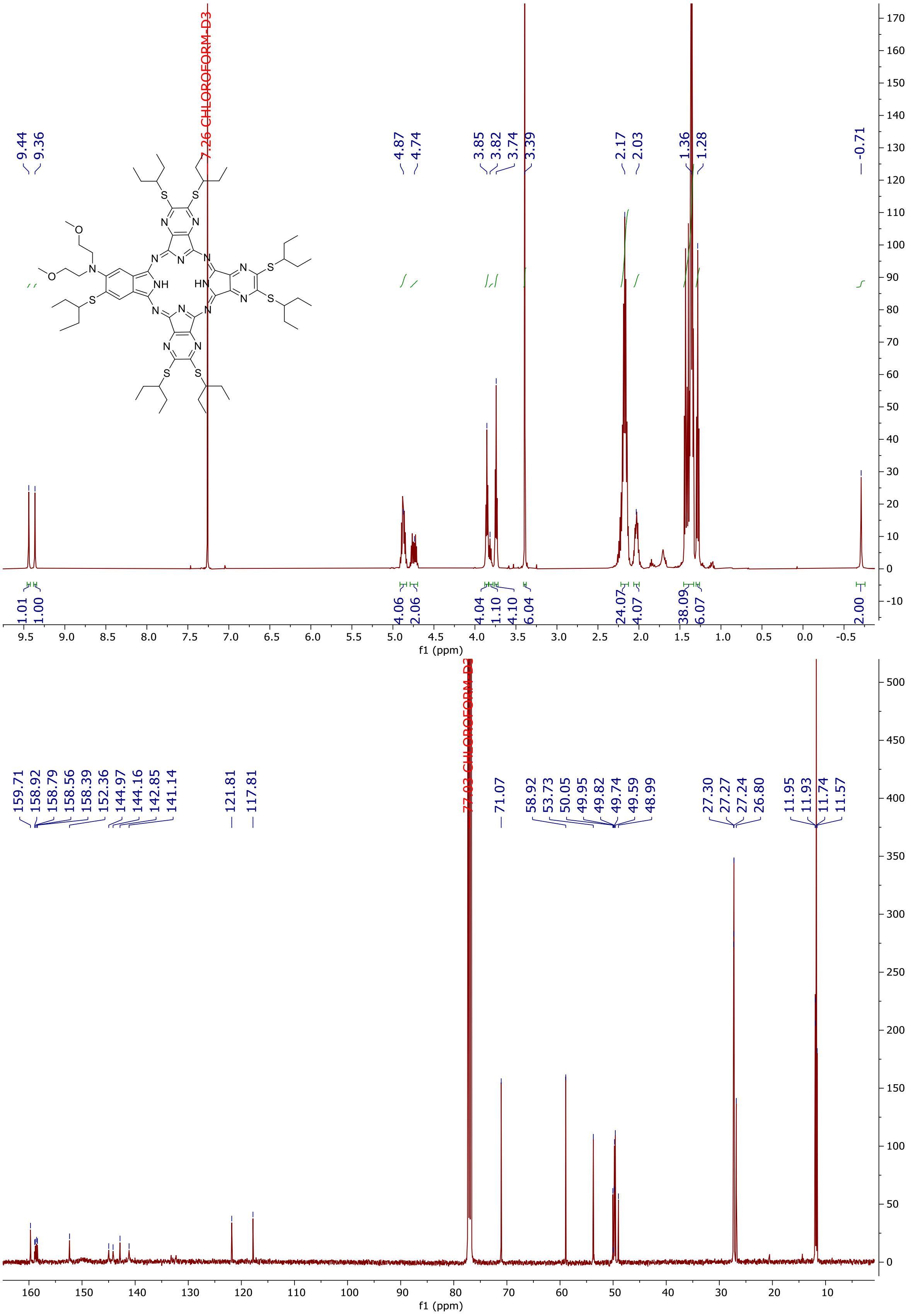

Figure S11: ^{1}H NMR (500 MHz, $CDCl_3$) and ^{13}C NMR (126 MHz, $CDCl_3$) of **Pc2-H**.

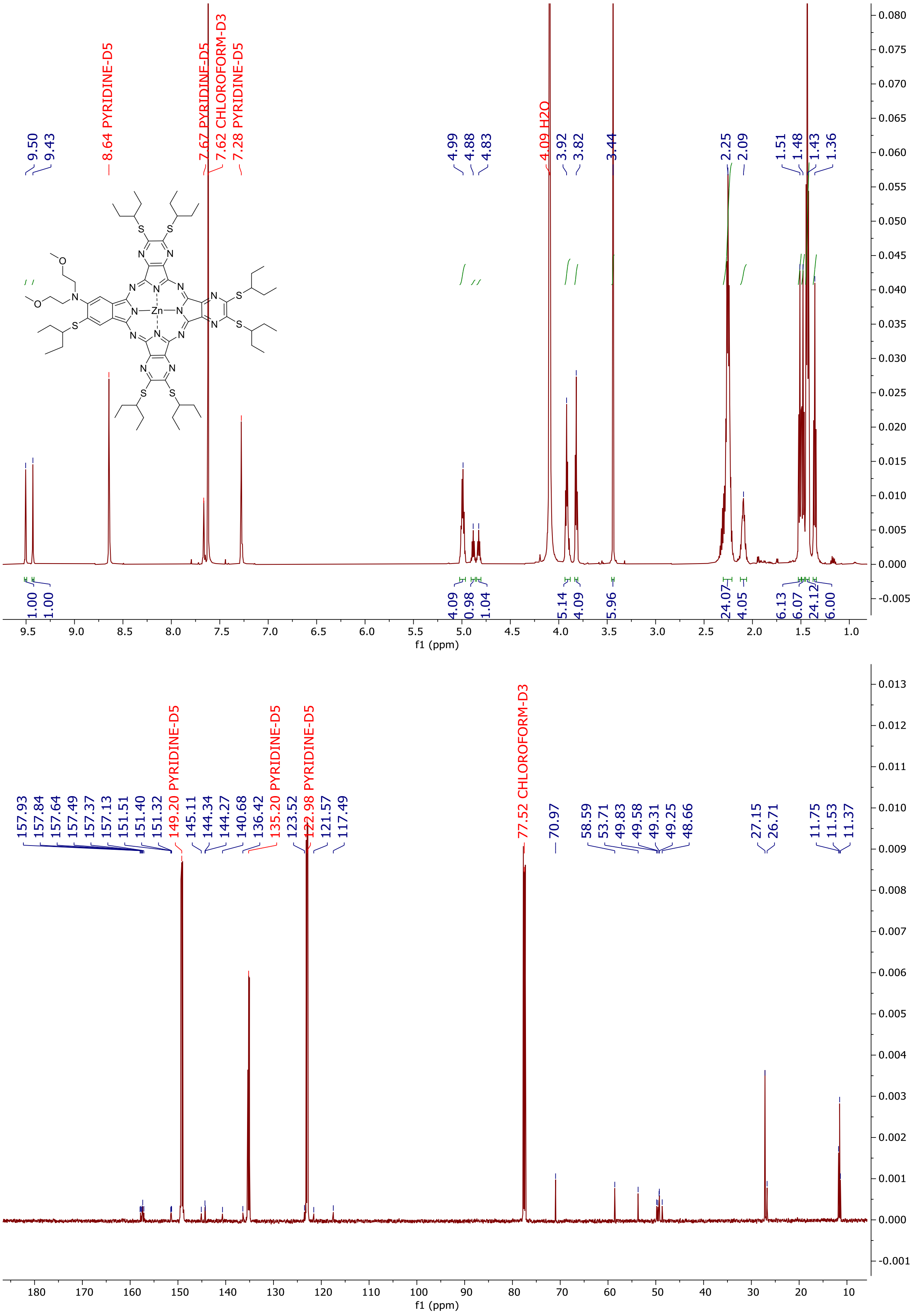

Figure S12: ^{1}H NMR (600 MHz, $CDCl_3$/pyridine-d_5 3:1) and ^{13}C NMR (151 MHz, $CDCl_3$/ pyridine-d_5 3:1) of **Pc2**.

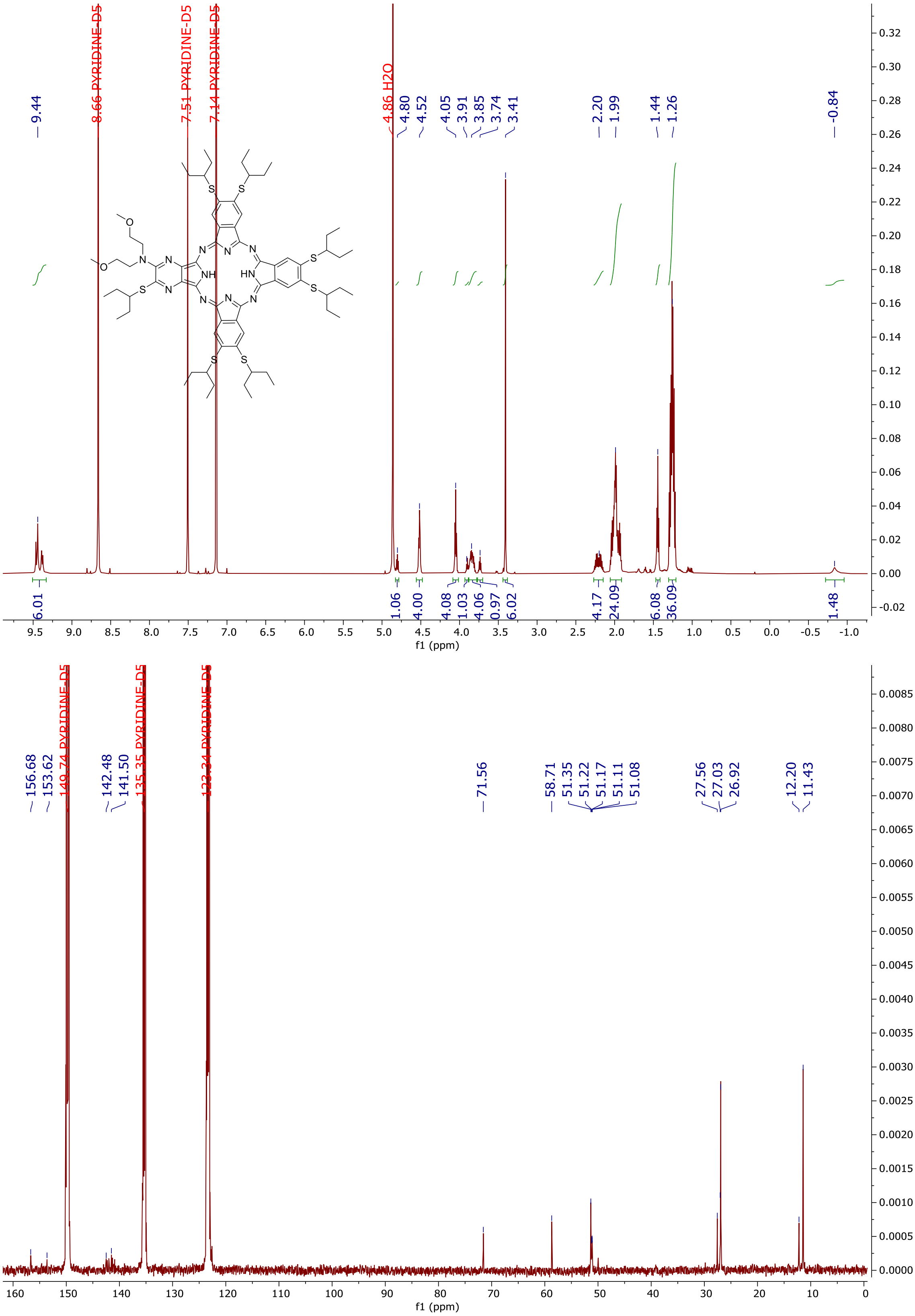

Figure S13: ^{1}H NMR (500 MHz, pyridine-d_5) and ^{13}C NMR (126 MHz, pyridine-d_5) of **Pc3-H**

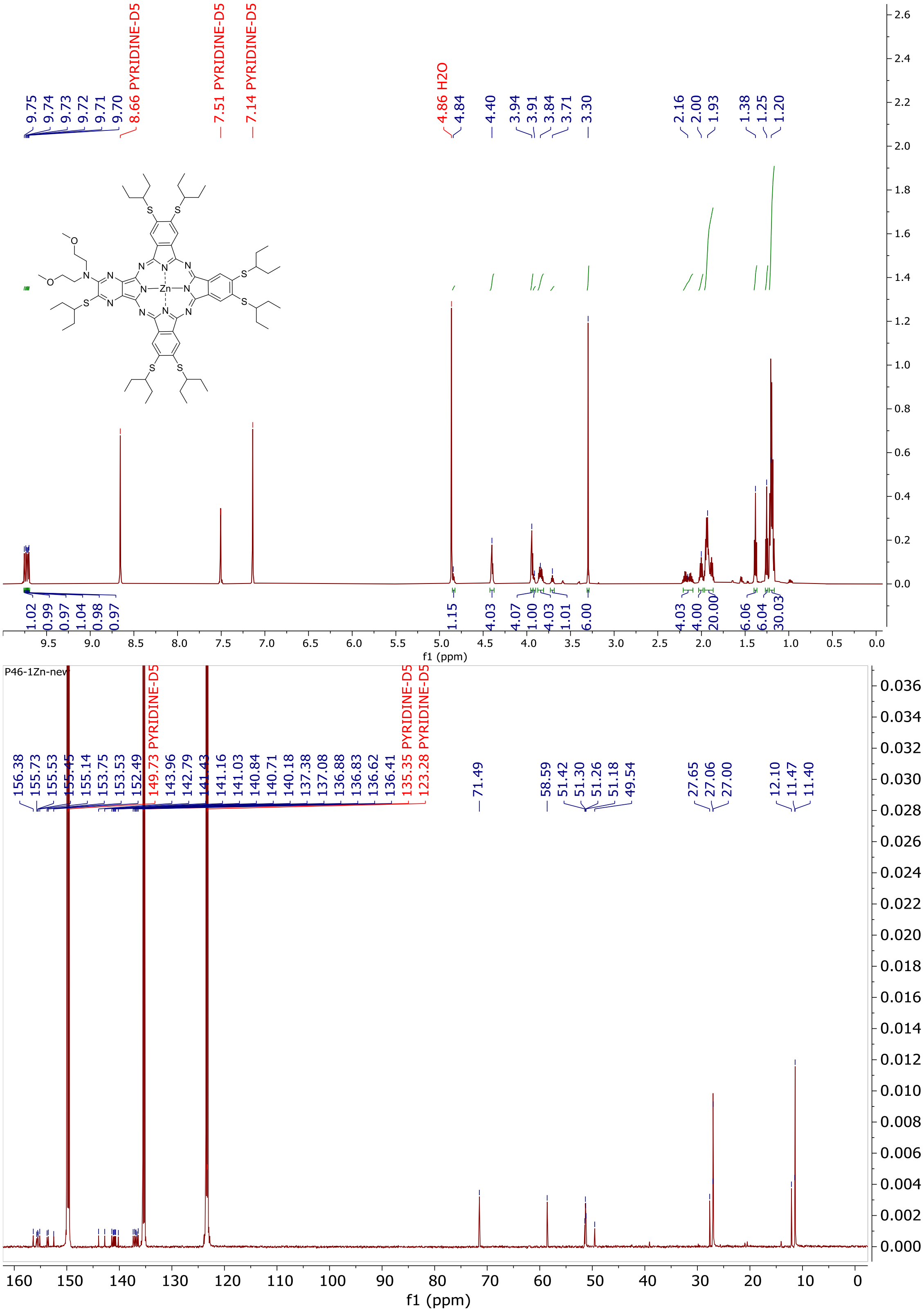

Figure S14: ^{1}H NMR (500 MHz, pyridine-d_5) and ^{13}C NMR (126 MHz, pyridine-d_5) of **Pc3**.

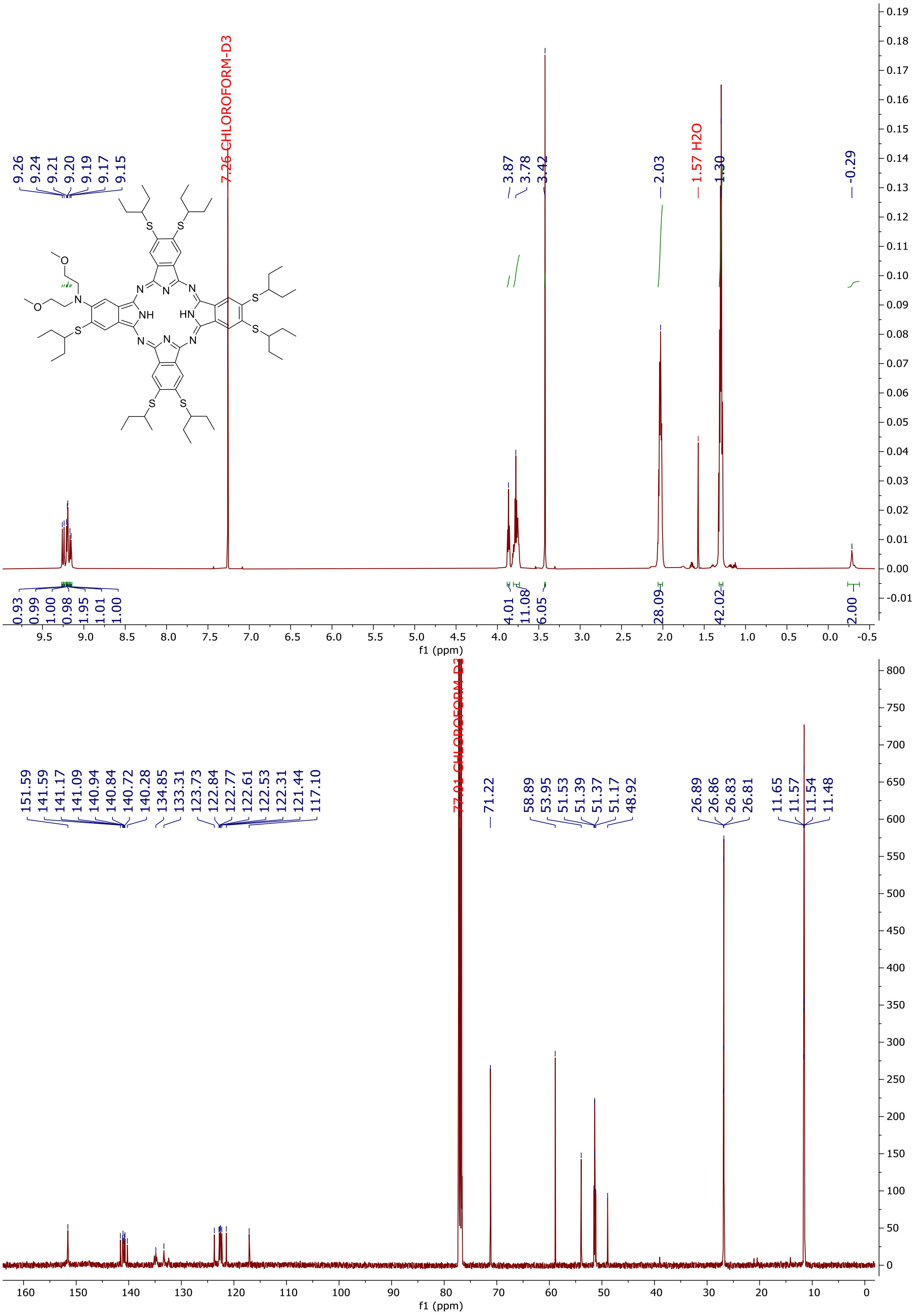

Figure S15: ^{1}H NMR (600 MHz, $CDCl_3$) and ^{13}C NMR (151 MHz, $CDCl_3$) of **Pc4-H**.

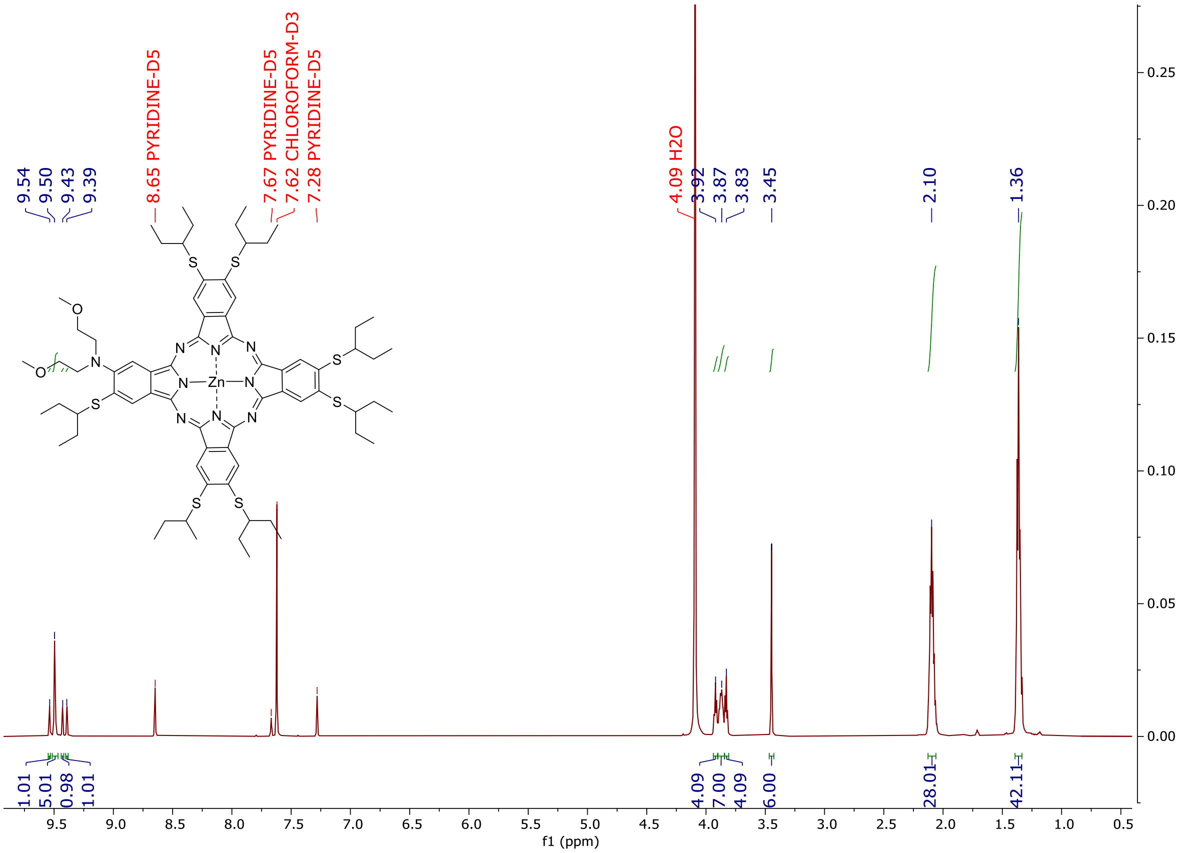

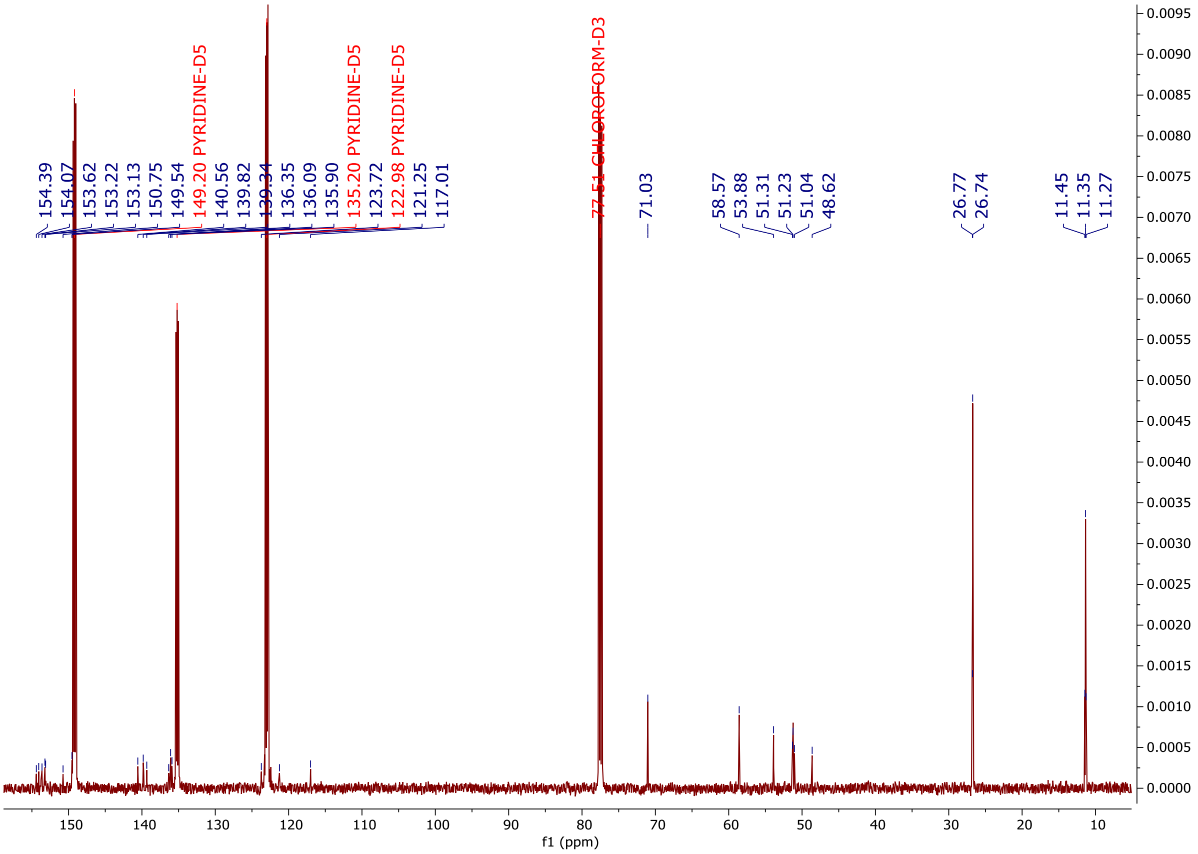

Figure S16: ^{1}H NMR (600 MHz, $CDCl_3$/pyridine-d_5 3:1) and ^{13}C NMR (151 MHz, $CDCl_3$/ pyridine-d_5 3:1) of **Pc4**.

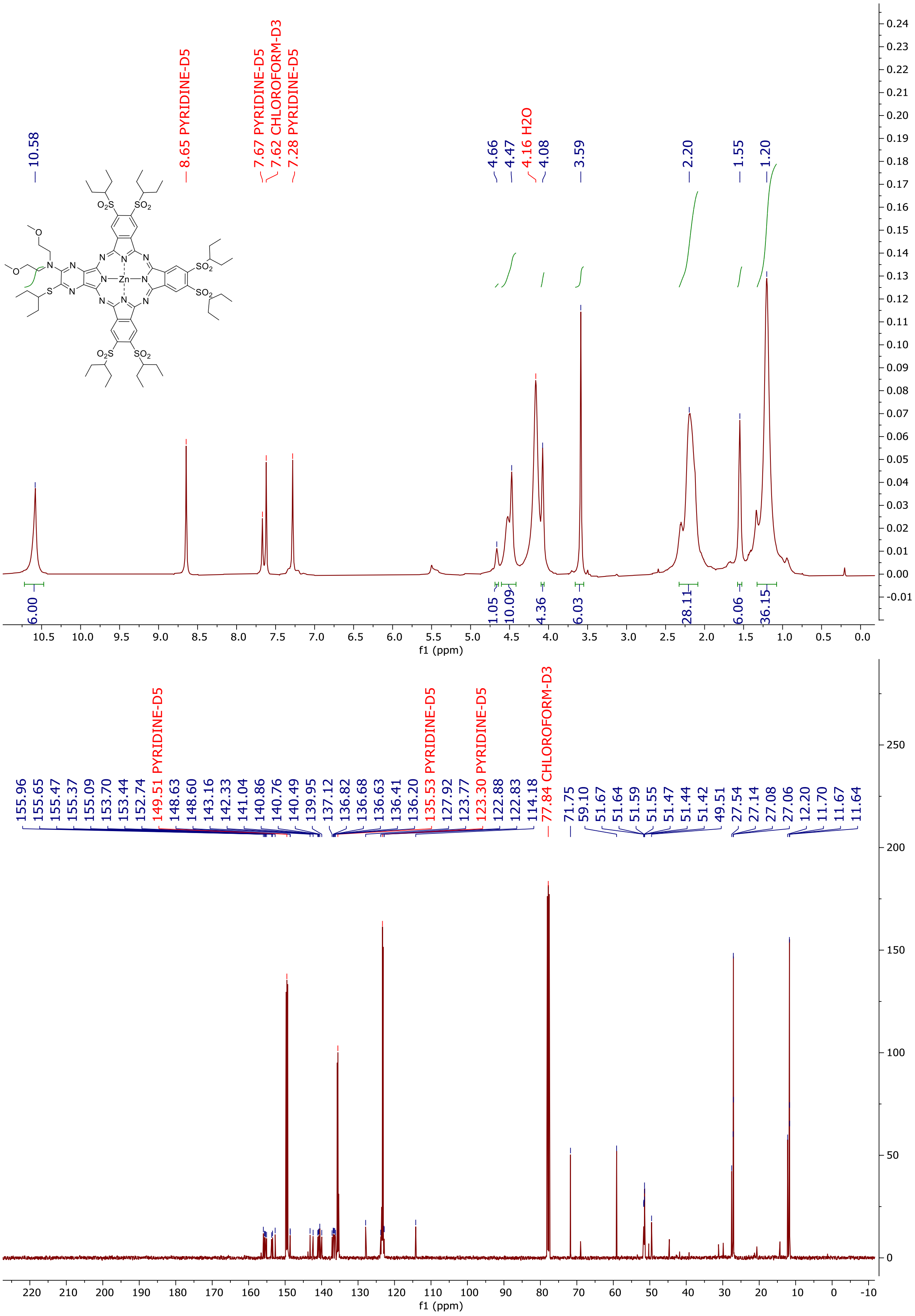

Figure S17: ^{1}H NMR (600 MHz, $CDCl_3$/pyridine-*d*5 3:1) and ^{13}C NMR (151 MHz, $CDCl_3$/ pyridine-*d*5 3:1) of **Pc5**.

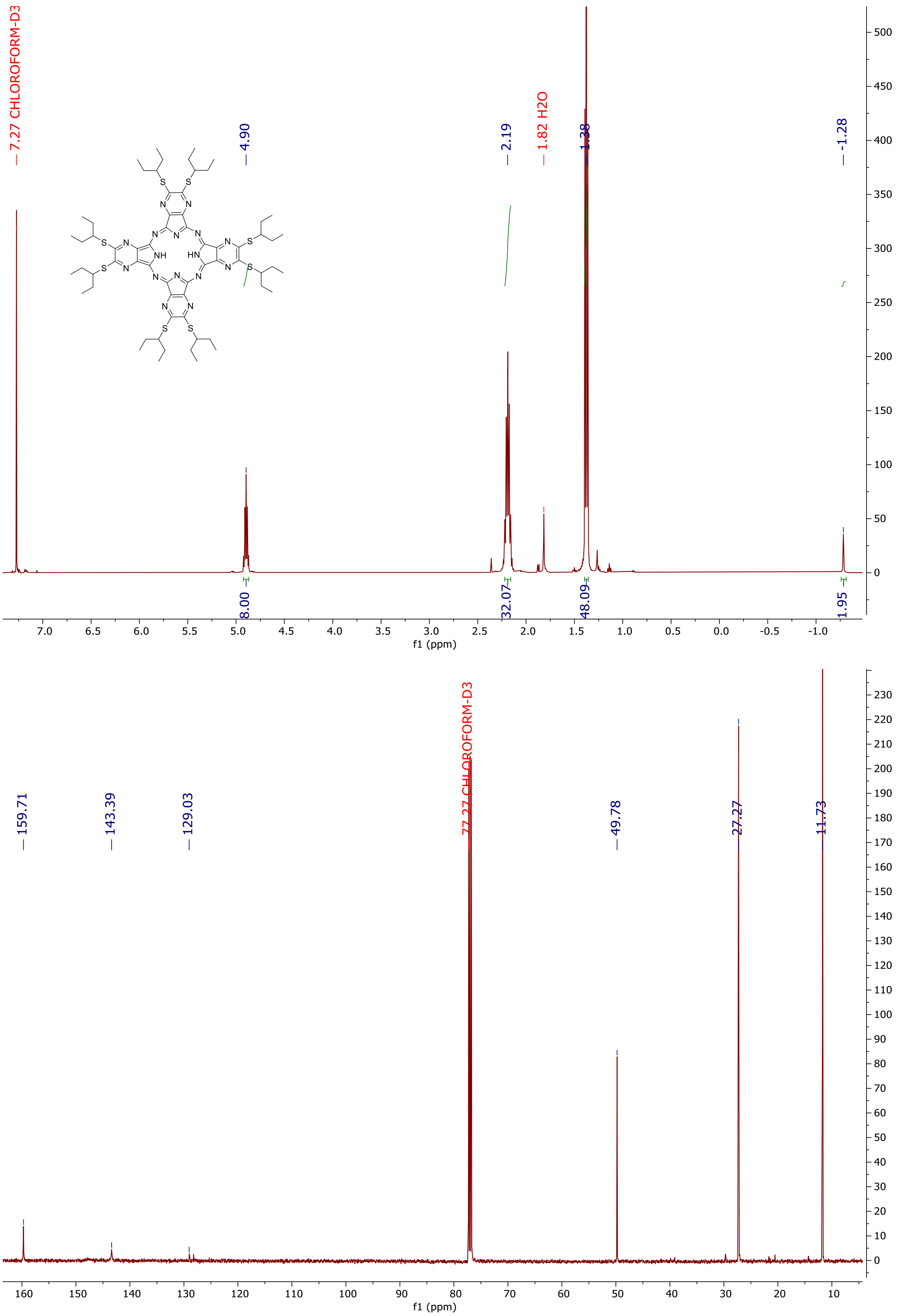

Figure S18: ^{1}H NMR (500 MHz, $CDCl_3$) and ^{13}C NMR (126 MHz, $CDCl_3$) of **Pc6-H**.

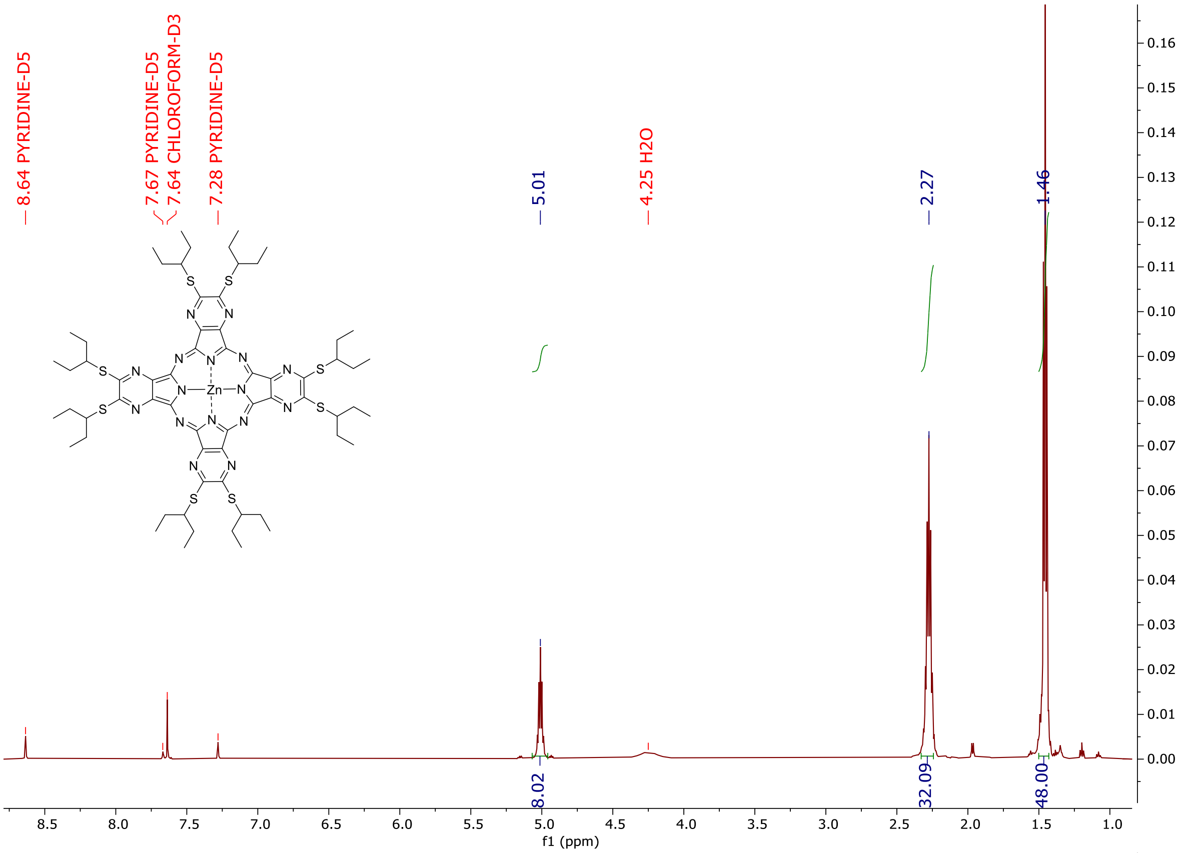

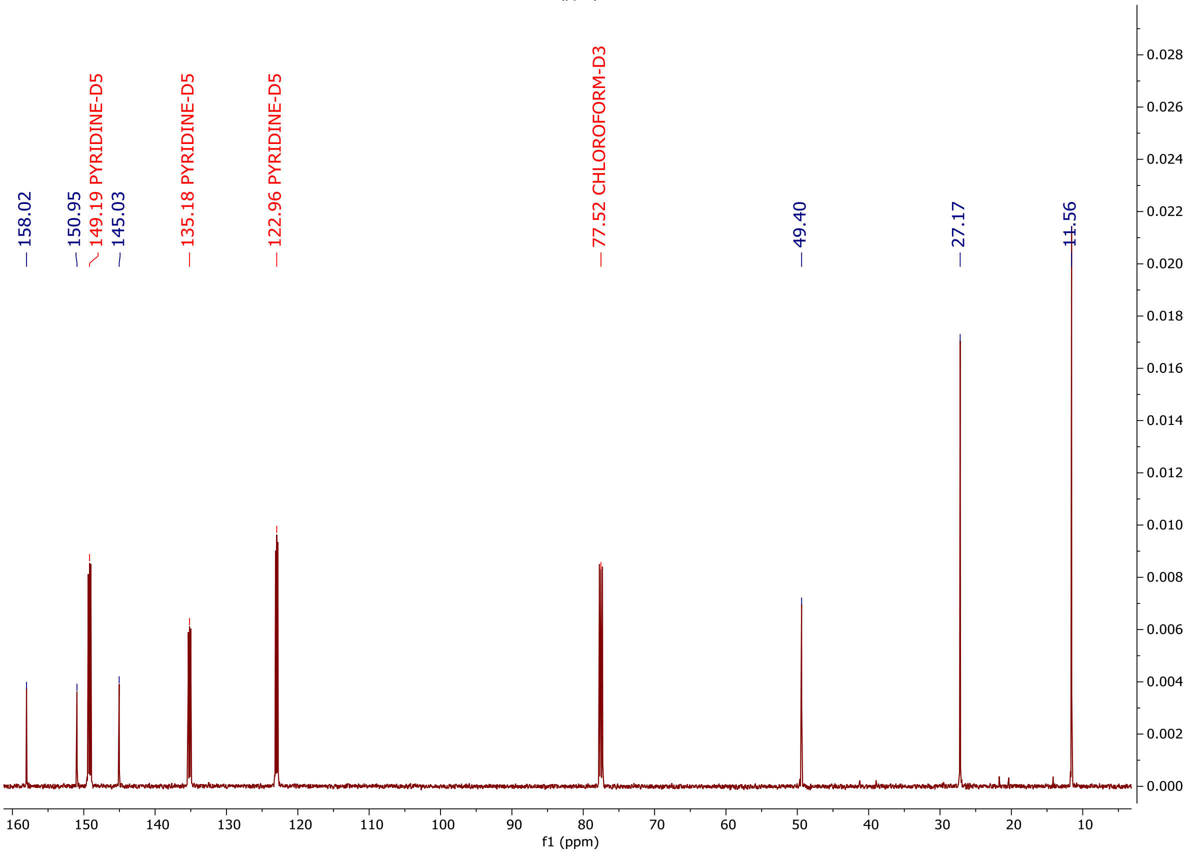

Figure S19: ^{1}H NMR (500 MHz, $CDCl_3$/pyridine-d_5 3:1) and ^{13}C NMR (126 MHz, $CDCl_3$/ pyridine-d_5 3:1) of **Pc6.**

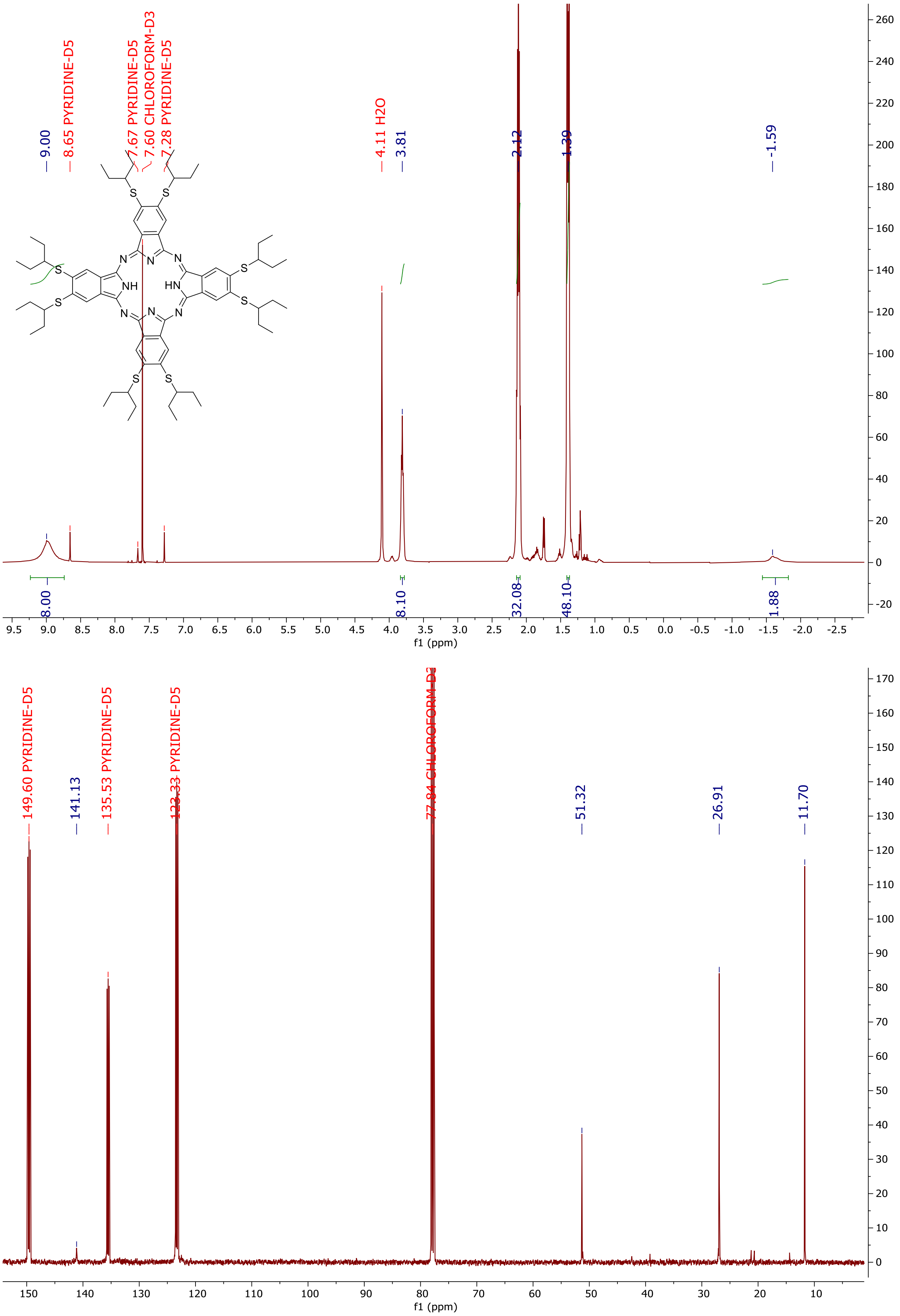

Figure S20: ^{1}H NMR (500 MHz, $CDCl_3$/pyridine-d_5 3:1) and ^{13}C NMR (126 MHz, $CDCl_3$/ pyridine-d_5 3:1) of **Pc7-H**

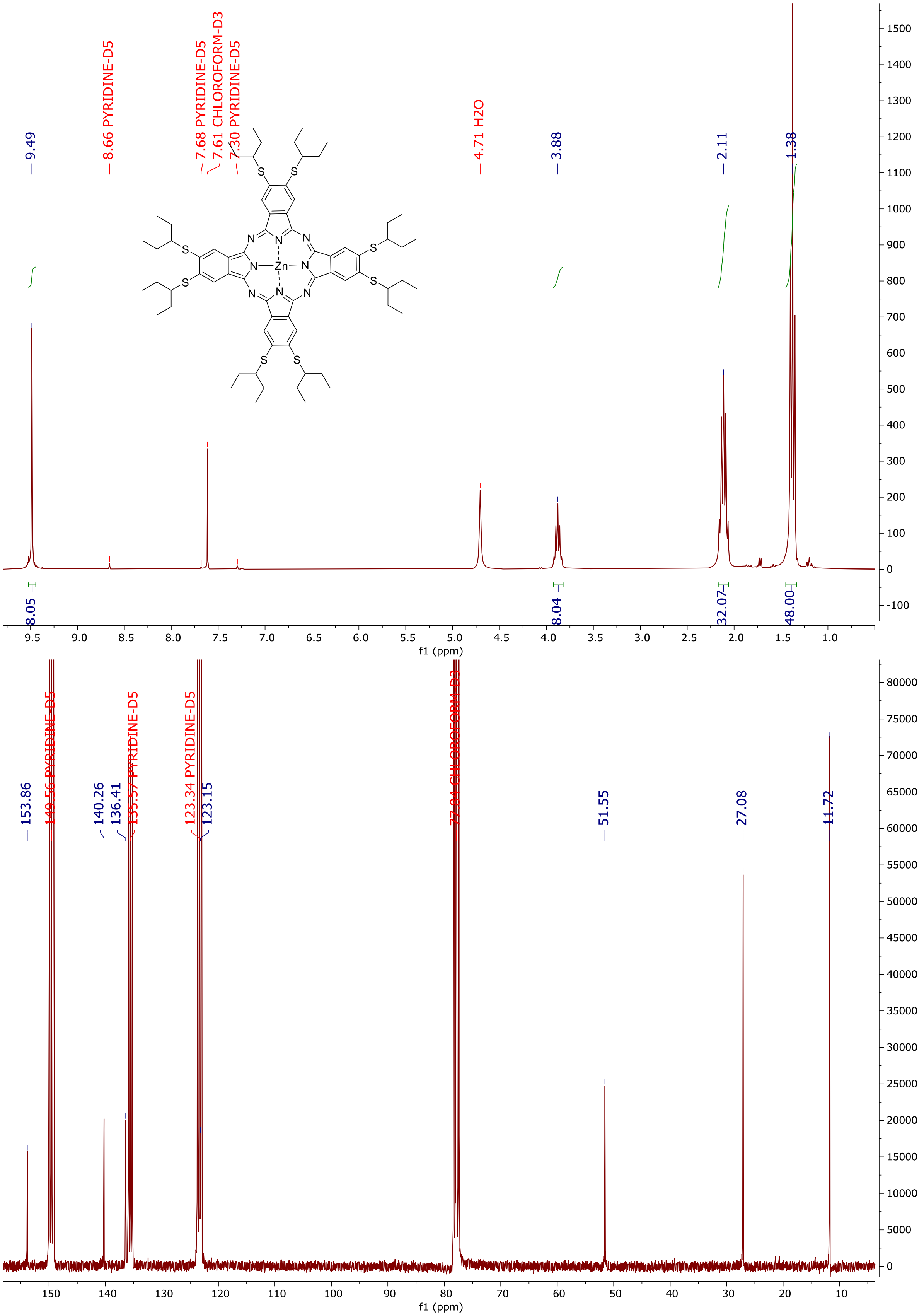

Figure S21: ^{1}H NMR (300 MHz, $CDCl_3$/pyridine-*d*5 3:1) and ^{13}C NMR (75 MHz, $CDCl_3$/ pyridine-*d*5 3:1) of **Pc7**.

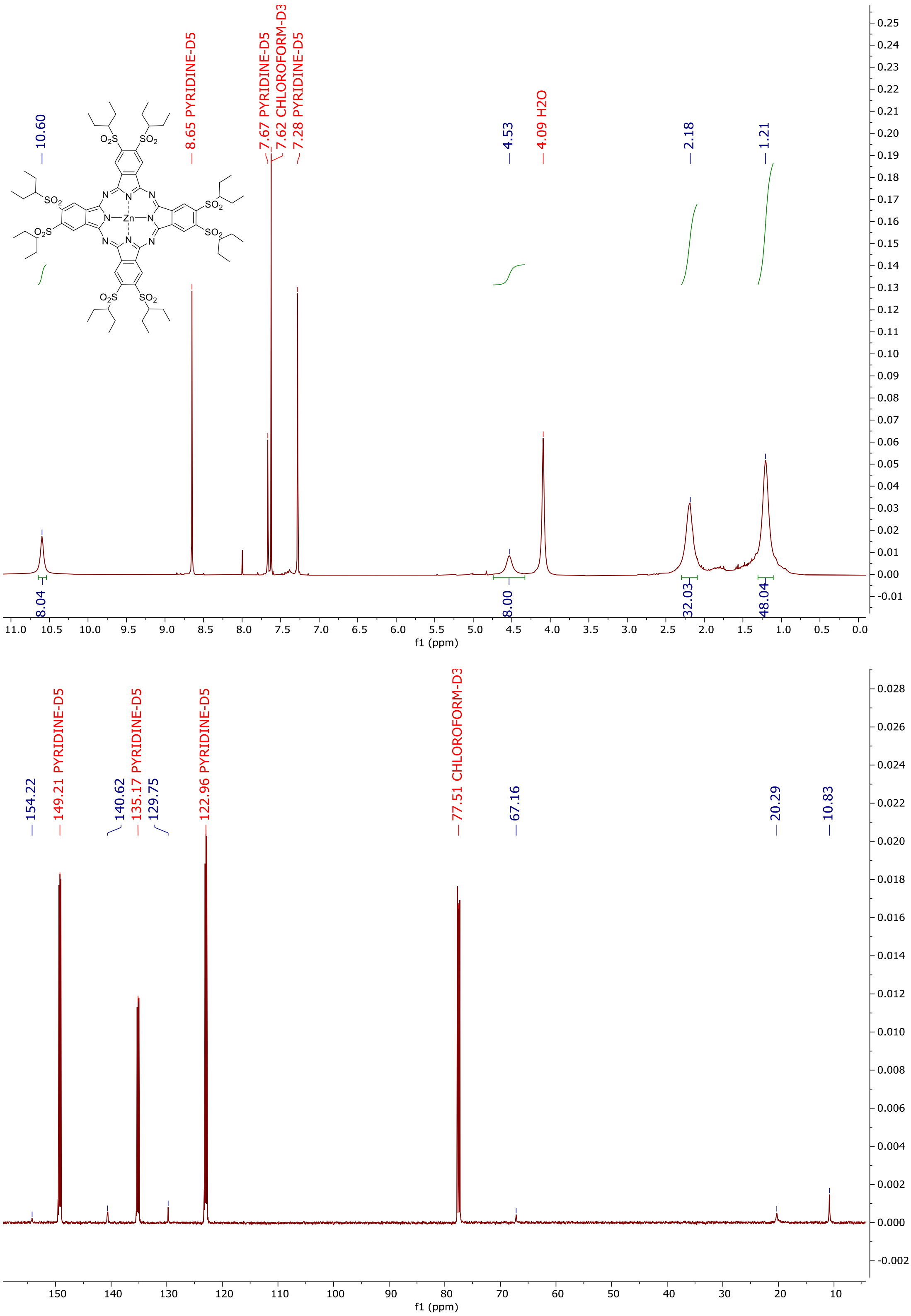

Figure S22: ^{1}H NMR (600 MHz, $CDCl_3$/pyridine-d_5 3:1) and ^{13}C NMR (151 MHz, $CDCl_3$/ pyridine-d_5 3:1) of **Pc8**.

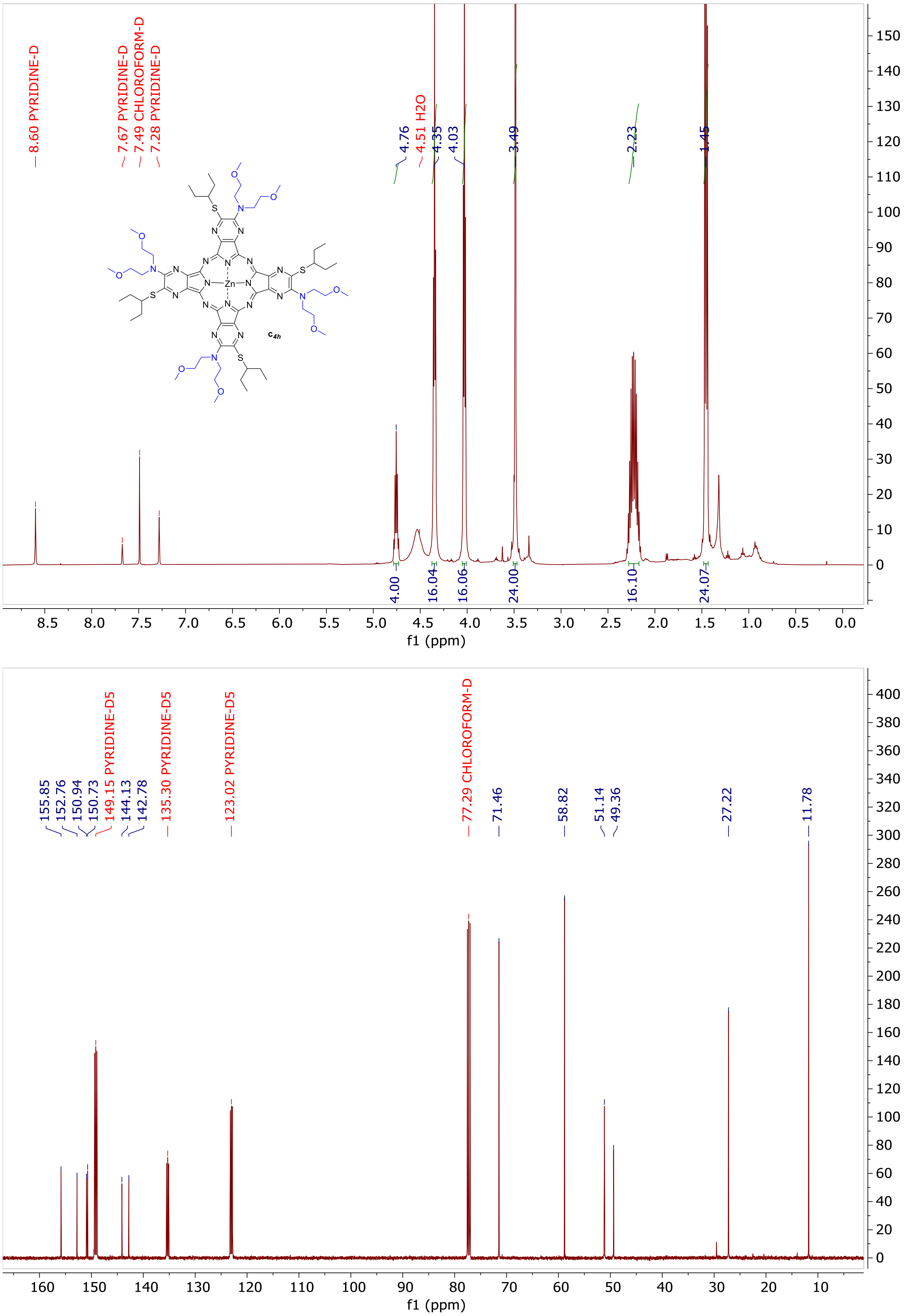

Figure S23: ^{1}H NMR (500 MHz, $CDCl_3$/pyridine-*d*$_5$ 3:1) and ^{13}C NMR (126 MHz, $CDCl_3$/ pyridine-*d*$_5$ 3:1) of **Pc9-C*4h***.

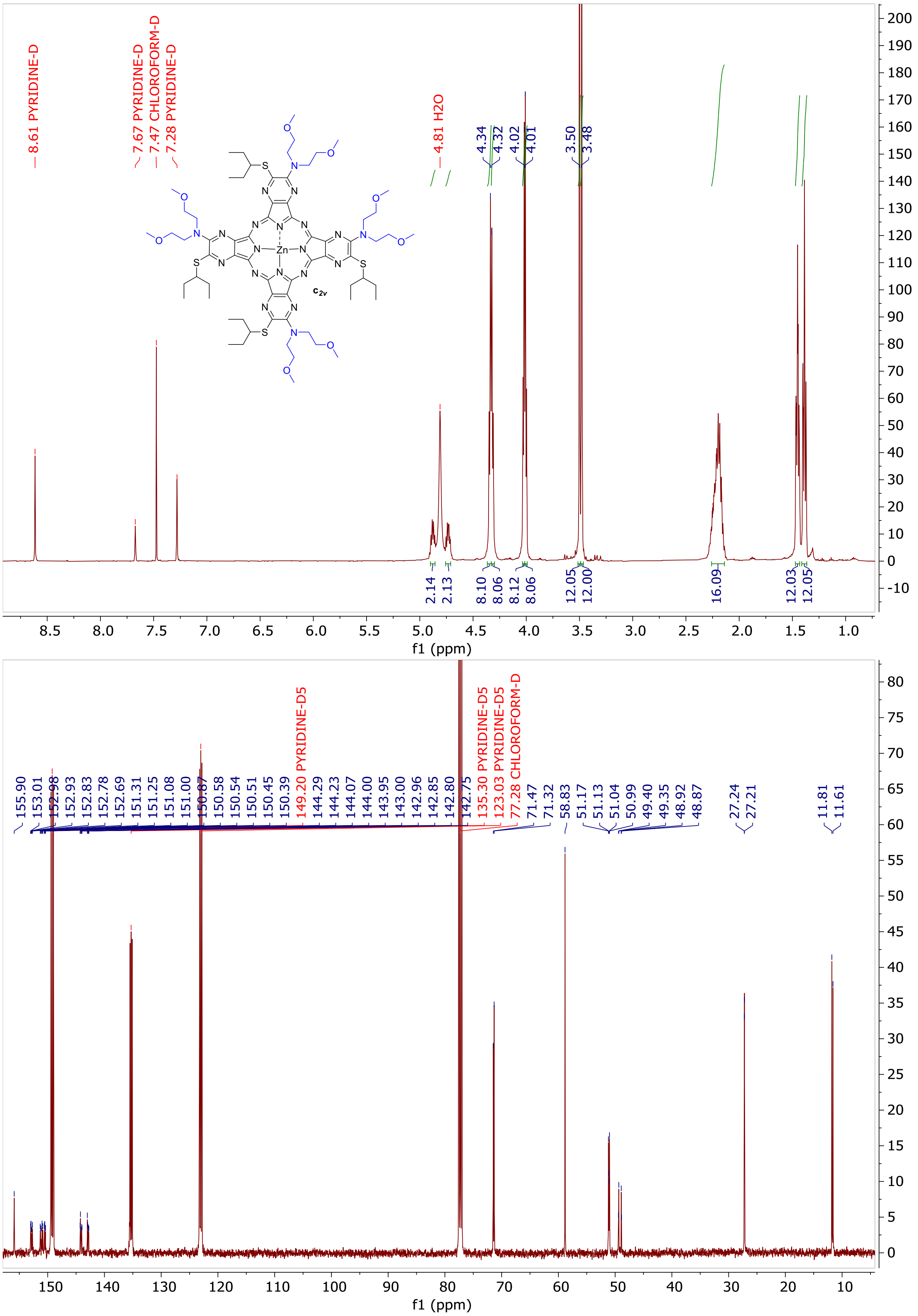

Figure S24: ^{1}H NMR (500 MHz, $CDCl_3$/pyridine-d_5 3:1) and ^{13}C NMR (126 MHz, $CDCl_3$/ pyridine-d_5 3:1) of **Pc9-C$_{2v}$**.

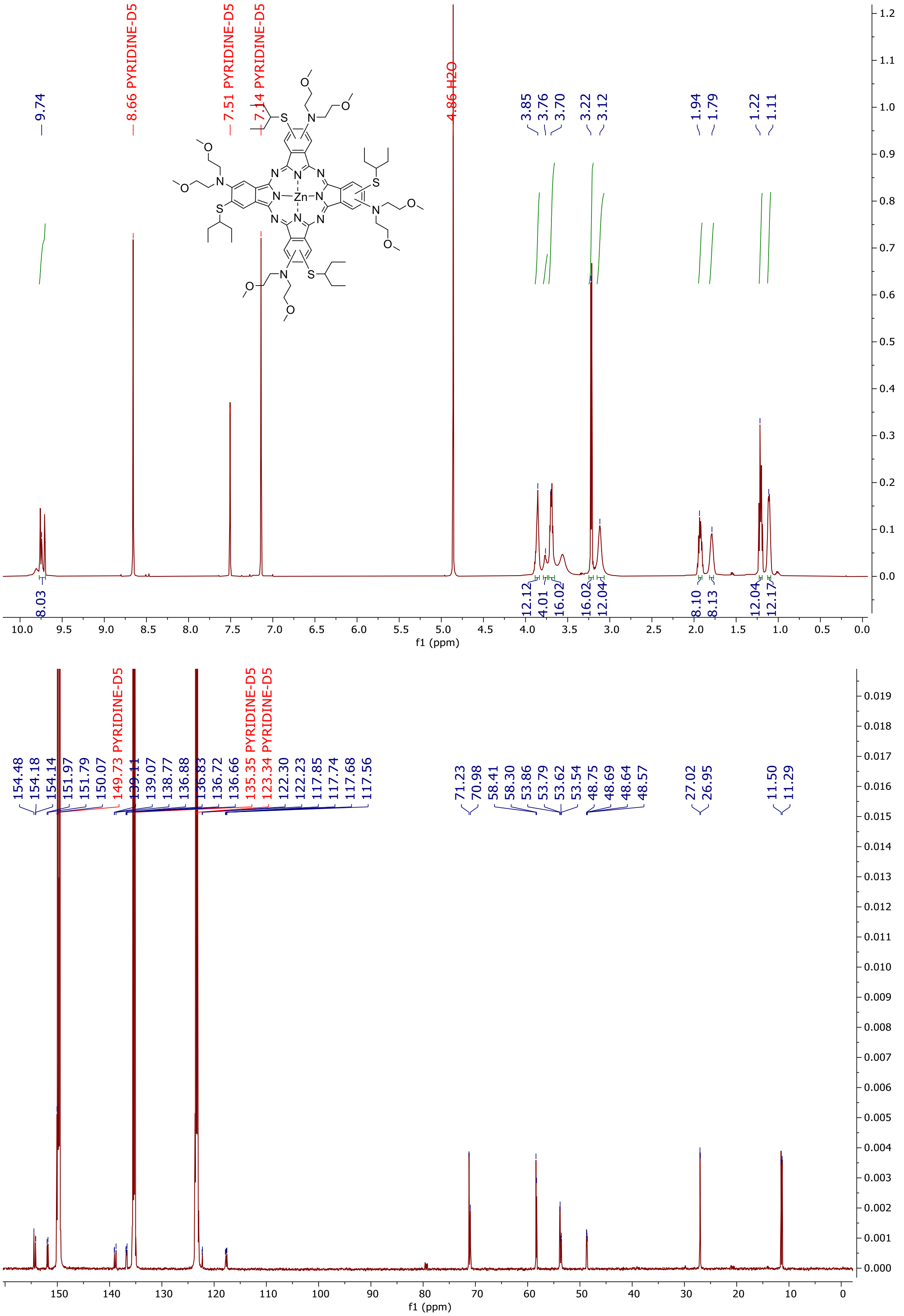

Figure S25: ^{1}H NMR (600 MHz, pyridine-*d*$_5$) and ^{13}C NMR (151 MHz, $CDCl_3$/ pyridine-*d*$_5$ 3:1) of **Pc10**.

Spectral properties of studied macrocycles

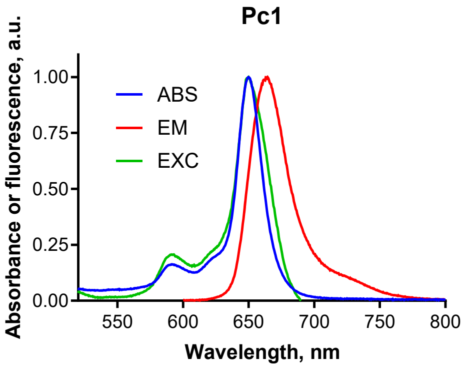

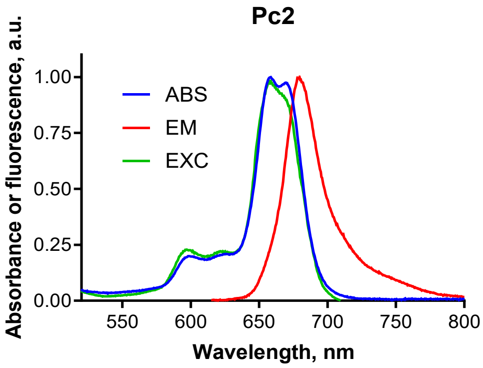

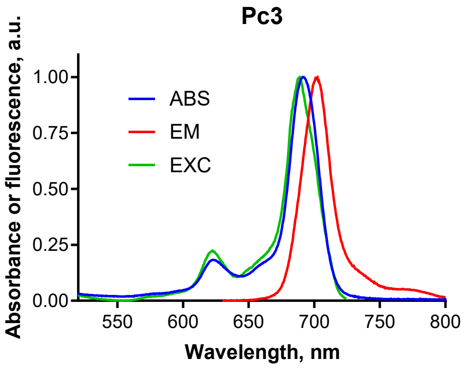

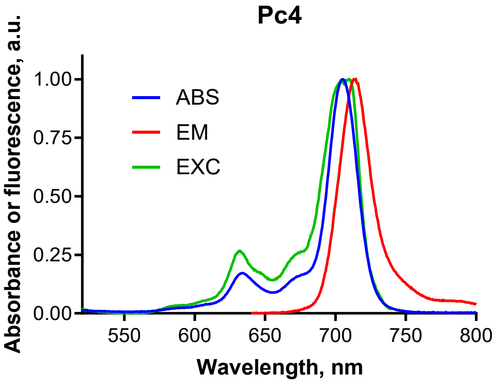

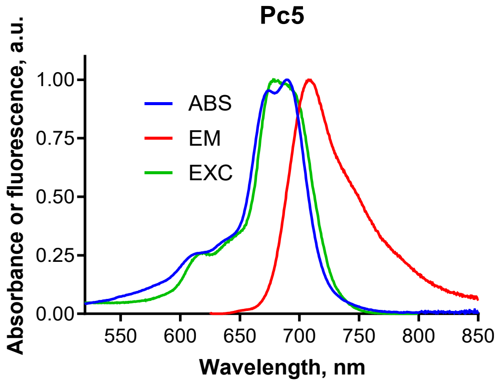

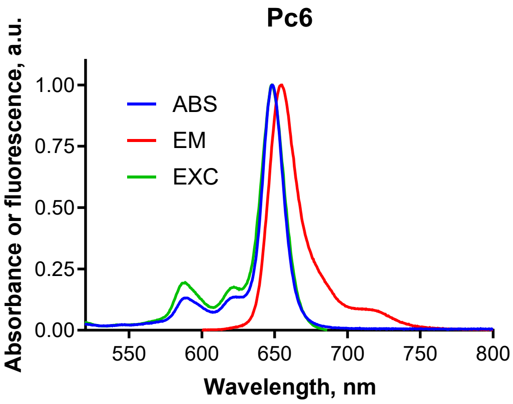

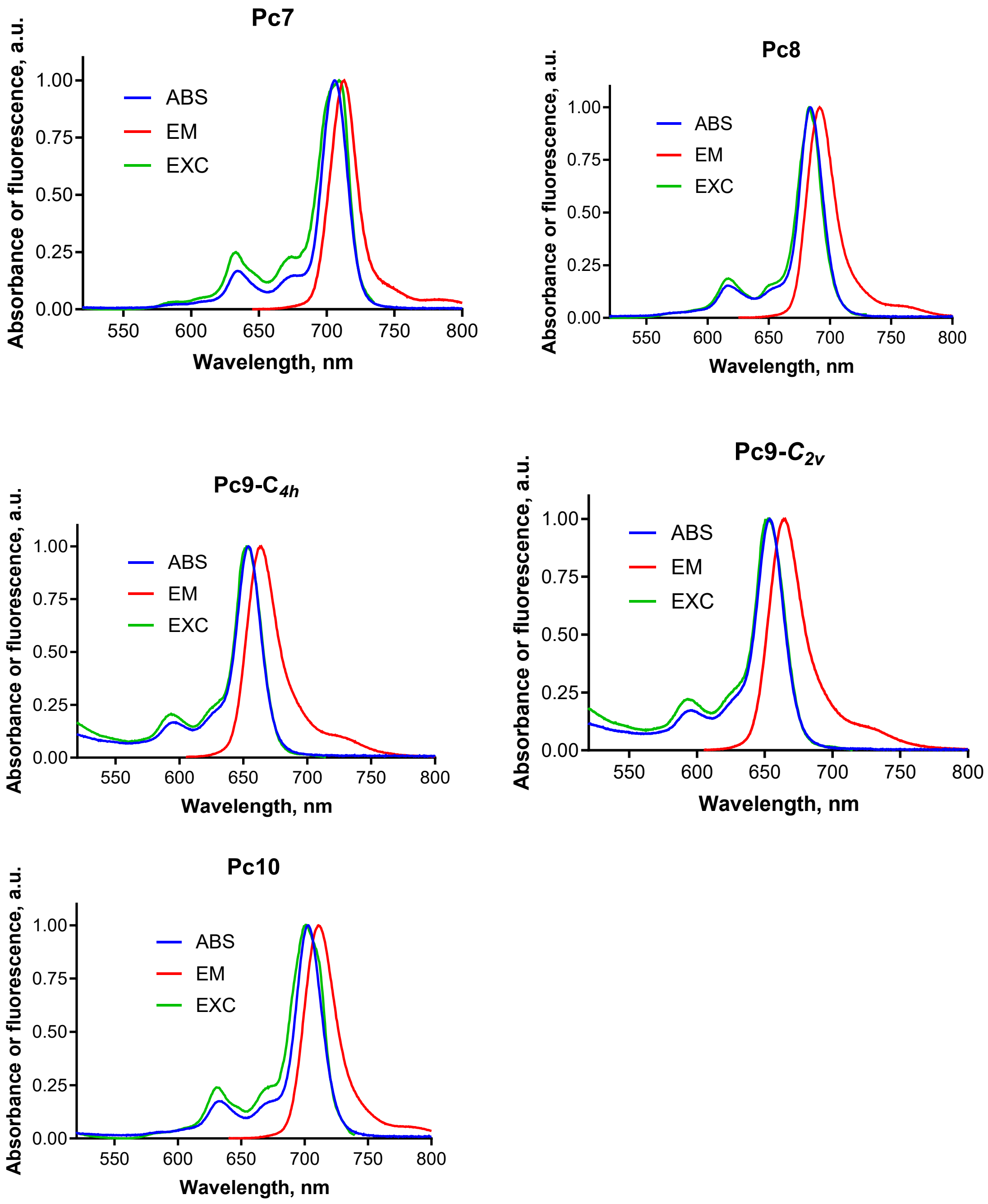

Figure S26: Normalized absorption (blue), fluorescence emission (red) and fluorescence excitation (green) spectra of studied macrocycles (c = 1μM) in THF.

Cyclic voltammograms and square wave voltammograms

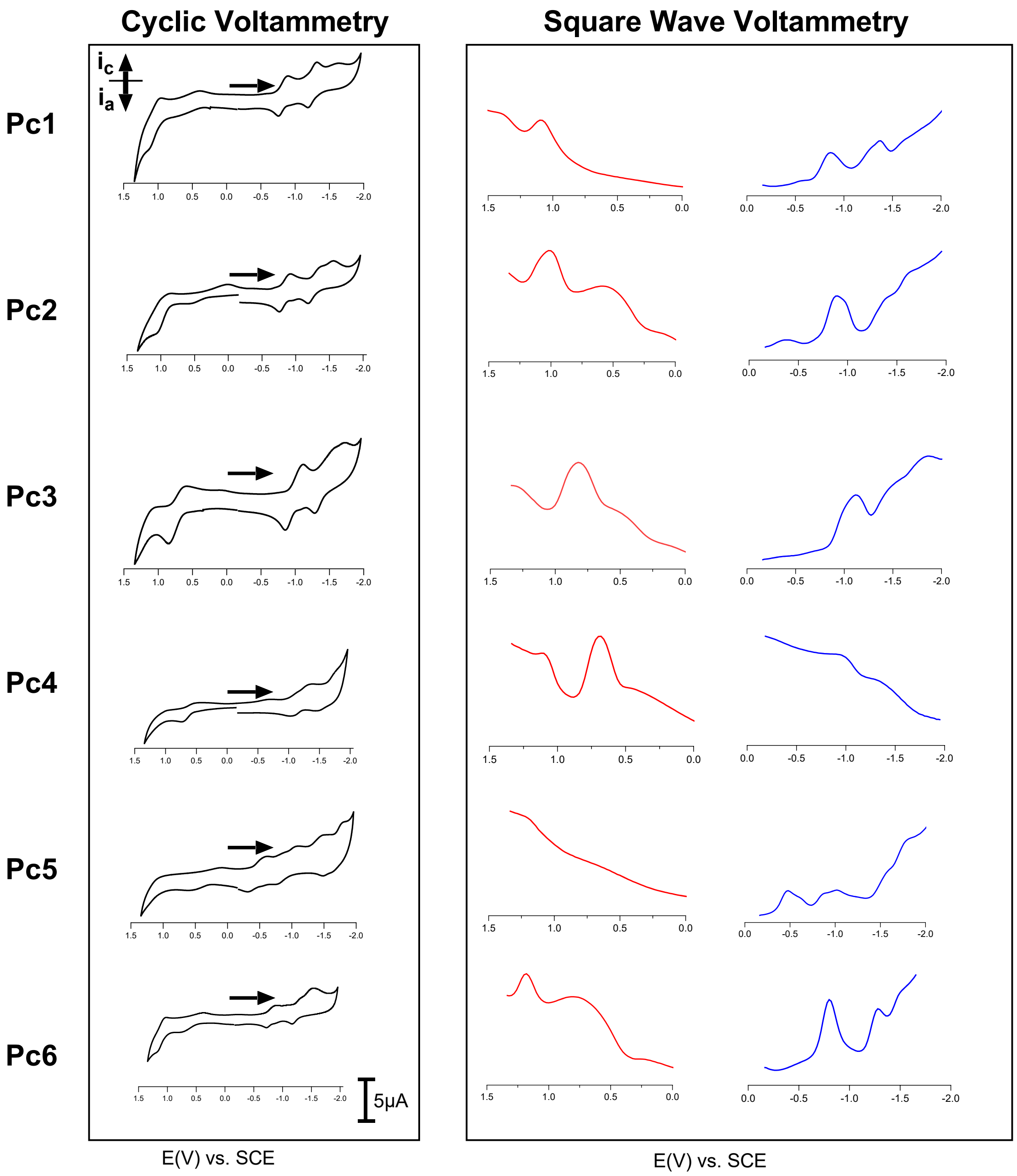

Fig. S27: Cyclic voltammograms and square wave voltammograms of **Pc1** – **Pc6** (in THF, rt, potential step 5 mV, scan rate 100mV/s, tetrabutylammonium hexafluorophosphate as supporting electrolyte, potential vs. SCE determined according to oxidation of ferrocene used as internal standard ($E_{(Fc/Fc+)}$ = 0.56 V vs. SCE[3]). Solutions of presented voltammograms do not contain ferrocene.

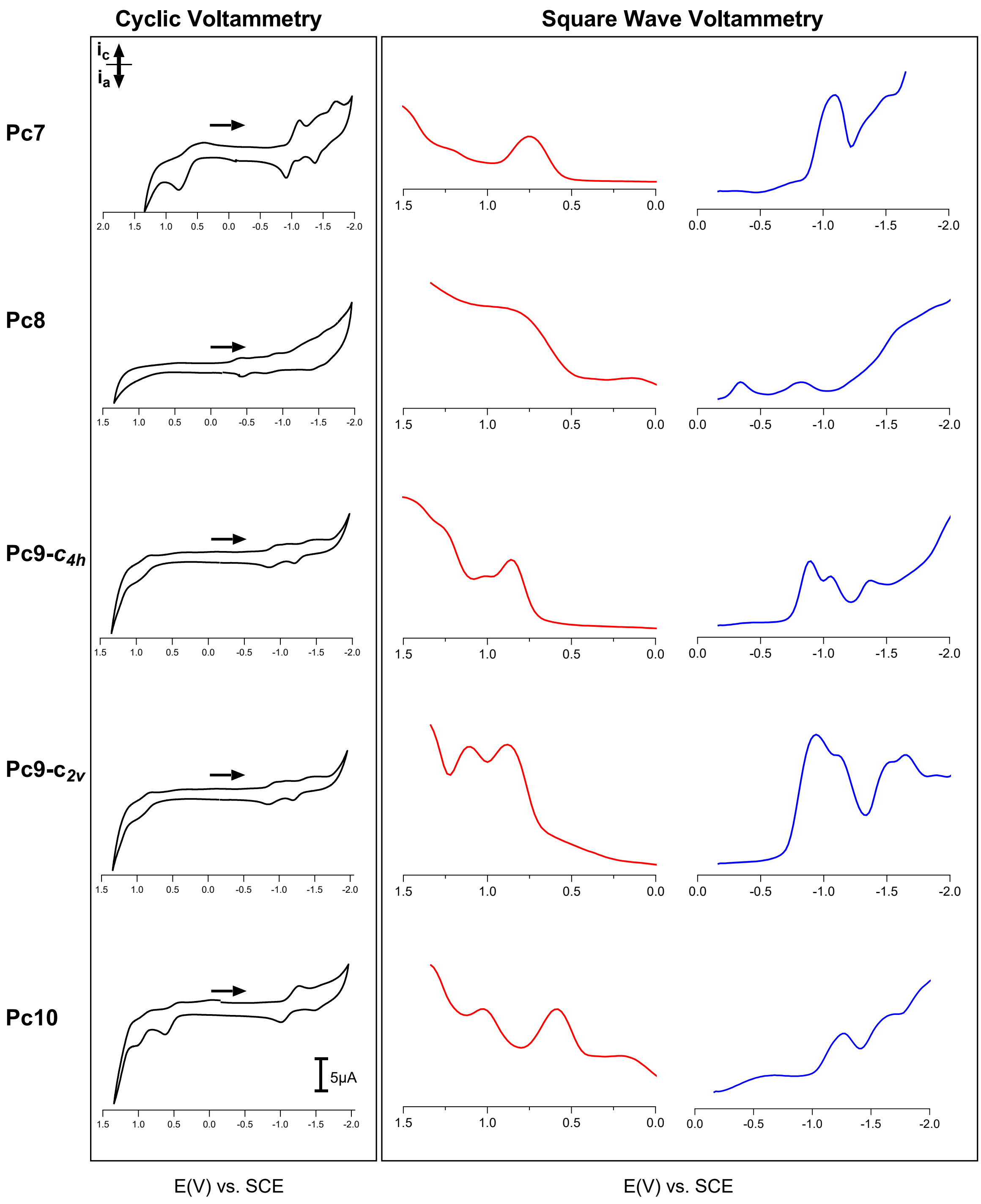

Fig. S28: Cyclic voltammograms and square wave voltammograms of **Pc7** – **Pc10** (in THF, rt, potential step 5 mV, scan rate 100mV/s, tetrabutylammonium hexafluorophosphate as supporting electrolyte, potential vs. SCE determined according to oxidation of ferrocene used as internal standard ($E_{(Fc/Fc+)}$ = 0.56 V vs. SCE[3]). Solutions of presented voltammograms do not contain ferrocene.

Fluorescence intensity decay curves of Pc1

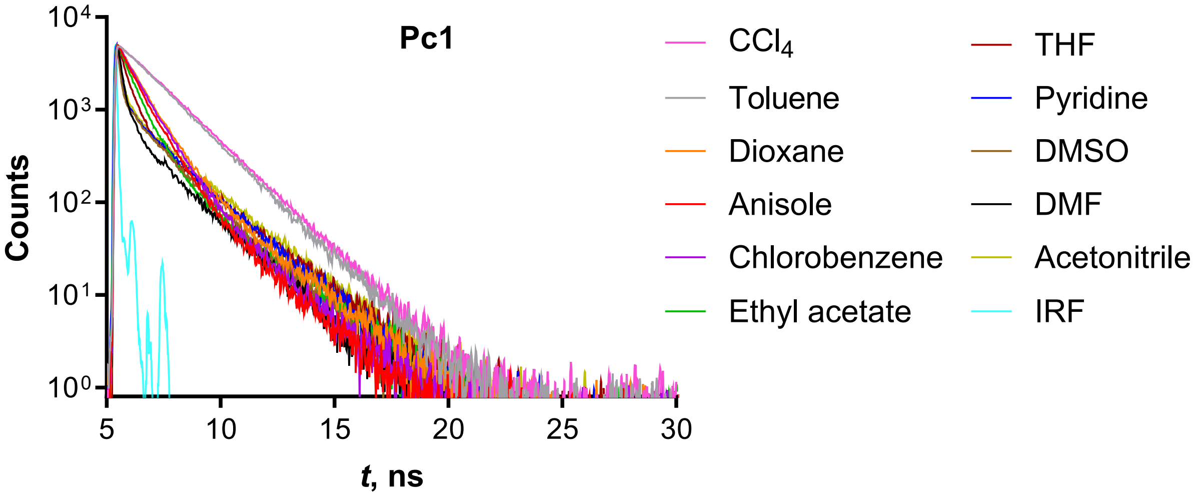

Fig. S29. Fluorescence intensity decay curves of compounds **Pc1** in different solvents. IRF = instrument response function.

References

1. Maya, E. M.; Vázquez, P.; Torres, T., Synthesis of Alkynyl-Linked Phthalocyanine Dyads: Push–Pull Homo- and Heterodimetallic Bisphthalocyaninato Complexes. *Chem. - Eur. J.* **1999,** 5, (7), 2004-2013.
2. Ragoussi, M.-E.; Katsukis, G.; Roth, A.; Malig, J.; de la Torre, G.; Guldi, D. M.; Torres, T., Electron-Donating Behavior of Few-Layer Graphene in Covalent Ensembles with Electron-Accepting Phthalocyanines. *J. Am. Chem. Soc.* **2014,** 136, (12), 4593-4598.
3. Connelly, N. G.; Geiger, W. E., Chemical redox agents for organometallic chemistry. *Chem. Rev.* **1996,** 96, (2), 877-910.
4. Zimcik, P.; Novakova, V.; Kopecky, K.; Miletin, M.; Uslu Kobak, R. Z.; Svandrlikova, E.; Váchová, L.; Lang, K., Magnesium Azaphthalocyanines: An Emerging Family of Excellent Red-Emitting Fluorophores. *Inorg. Chem.* **2012,** 51, (7), 4215-4223.
5. Musil, Z.; Zimcik, P.; Miletin, M.; Kopecky, K.; Link, M.; Petrik, P.; Schwarz, J., Synthesis and singlet oxygen production of azaphthalocyanines bearing functional derivatives of carboxylic acid. *J. Porphyrins Phthalocyanines* **2006,** 10, (2), 122-131.
6. Kaestner, L.; Cesson, M.; Kassab, K.; Christensen, T.; Edminson, P. D.; Cook, M. J.; Chambrier, I.; Jori, G., Zinc octa-n-alkyl phthalocyanines in photodynamic therapy: photophysical properties, accumulation and apoptosis in cell cultures, studies in erythrocytes and topical application to Balb/c mice skin. *Photochem. Photobiol. Sci.* **2003,** 2, (6), 660-667.
7. Michelsen, U.; Kliesch, H.; Schnurpfeil, G.; Sobbi, A. K.; Wohrle, D., Unsymmetrically substituted benzonaphthoporphyrazines: A new class of cationic photosensitizers for the photodynamic therapy of cancer. *Photochem. Photobiol.* **1996,** 64, (4), 694-701.
